# The effects of public health policies on health inequalities in high-income countries: an umbrella review

**DOI:** 10.1186/s12889-018-5677-1

**Published:** 2018-07-13

**Authors:** Katie Thomson, Frances Hillier-Brown, Adam Todd, Courtney McNamara, Tim Huijts, Clare Bambra

**Affiliations:** 1 Institute of Health and Society, Newcastle University, Richardson Road, Newcastle upon Tyne, NE2 4AX UK; 2 Fuse – UKCRC Centre for Translational Research in Public Health, Newcastle upon Tyne, UK; 3 School of Applied Social Sciences, Durham University, 32 Old Elvet, Durham, DH1 3HN UK; 4 School of Pharmacy, Faculty of Medical Sciences, Newcastle University, King George VI Building, Newcastle upon Tyne, NE1 7RU UK; 5 Department of Sociology and Political Science, Norwegian University of Science and Technology, Building 9, Level 5, 7491 Dragvoll, Trondheim, Norway; 6 Research Centre for Education and the Labour Market, Maastricht University, Tongersestraat 53, 6211 LM Maastricht, The Netherlands

**Keywords:** Social determinants of health, Equity, Regulation, Evaluation, Intervention

## Abstract

**Background:**

Socio-economic inequalities are associated with unequal exposure to social, economic and environmental risk factors, which in turn contribute to health inequalities. Understanding the impact of specific public health policy interventions will help to establish causality in terms of the effects on health inequalities.

**Methods:**

Systematic review methodology was used to identify systematic reviews from high-income countries that describe the health equity effects of upstream public health interventions. Twenty databases were searched from their start date until May 2017. The quality of the included articles was determined using the Assessment of Multiple Systematic Reviews tool (AMSTAR).

**Results:**

Twenty-nine systematic reviews were identified reporting 150 unique relevant primary studies. The reviews summarised evidence of all types of primary and secondary prevention policies (fiscal, regulation, education, preventative treatment and screening) across seven public health domains (tobacco, alcohol, food and nutrition, reproductive health services, the control of infectious diseases, the environment and workplace regulations). There were no systematic reviews of interventions targeting mental health. Results were mixed across the public health domains; some policy interventions were shown to reduce health inequalities (e.g. food subsidy programmes, immunisations), others have no effect and some interventions appear to increase inequalities (e.g. 20 mph and low emission zones). The quality of the included reviews (and their primary studies) were generally poor and clear gaps in the evidence base have been highlighted.

**Conclusions:**

The review does tentatively suggest interventions that policy makers might use to reduce health inequalities, although whether the programmes are transferable between high-income countries remains unclear.

**Trial registration:**

PROSPERO registration number: CRD42016025283

**Electronic supplementary material:**

The online version of this article (10.1186/s12889-018-5677-1) contains supplementary material, which is available to authorized users.

## Background

In high-income countries like the UK, USA and Sweden, the welfare state plays an integral role in influencing the social determinants of health. The *welfare state* relates to post-World War Two government measures for the provision of key services and social transfers including the state’s role in education, health, housing, poor relief, social insurance, and public health policy in high-income countries [1]. The most encompassing and extensive welfare states are found in Europe where countries are able to use a variety of policy mechanisms - namely public health policies, social policies (e.g. cash transfers, housing and education) and healthcare services - to improve the health of their citizens and mitigate the health effects of socio-economic inequalities [2]. Previous research conducted in this area has generally found that countries with welfare provision, such as Sweden or Norway, have better population health than those with less generous social safety nets [2]. However, socio-economic inequalities in health still remain widespread across Europe and elsewhere; for example, people with higher income, occupation or education have lower mortality and morbidity [2–4]. In addition, comparative research examining how differences in the magnitude of health inequalities vary by welfare state has not found consistent evidence of lower health inequalities in the more extensive welfare states – this observation has been termed the Nordic public health puzzle [5, 6]. The majority of this research, however, has examined general associations between welfare state regime *types* and health inequalities. There has been very little research examining the effects of specific welfare state *policies* on health inequalities – especially in respect to public health policies. Consequently, this review aims to examine the effects of public health policies on health inequalities in high-income welfare states.

## Methods

### Conceptual framework

**Fig. 1:**
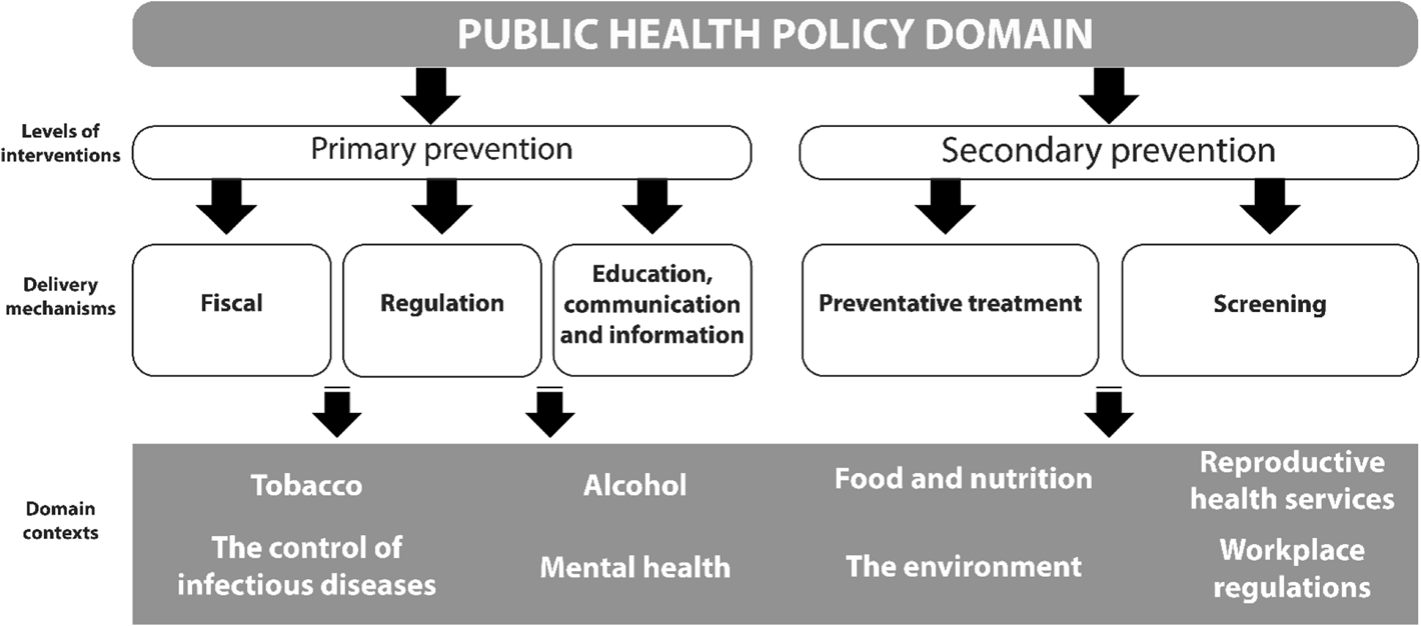
Conceptual framework of population-level preventative public health policies to reduce health inequalities

Hawe and Potvin [7] describe public health policy interventions as *‘policies or programs that shift the distribution of health risk by addressing the underlying social, economic and environmental conditions’* (pg. 8) acting at both primary and secondary prevention levels [8]. Further, Main et al. [9] define population-level interventions as those applied to populations, groups, areas, jurisdictions or institutions. Mackenbach and McKee [10] identify several key public health *policy domains*: tobacco, alcohol, food and nutrition, reproductive health services, the control of infectious diseases, mental health, the environment (road traffic injuries, air, land and water pollution) and workplace regulations. We further categorise public health policies by specific *mechanisms of delivery* into: fiscal policy, regulation, education, preventative treatment and screening. The interactions of these *levels of intervention*, the specific *policy domains*, and the *delivery mechanisms* are shown in Fig. 1 – the framework which informs our umbrella review.

### Design

Umbrella reviews - overviews of systematic reviews - build on the strengths of individual reviews and add scale by integrating the findings of multiple reviews together [11]. The full methodology has been previously described in the published protocol [12]; the review was registered with PROSPERO, the International Prospective Register of Systematic Reviews (registration number: CRD42016025283). A PRISMA statement is also included in Additional file 1: Appendix S1.

### Search strategy

The following 20 electronic databases were searched from start date to 04/05/2017 (host sites given in parentheses): Medline (Ovid), Embase (Ovid), Cumulative Index to Nursing and Allied Health Literature (CINAHL; EBSCOhost), PsycINFO (EBSCOhost), Social Science Citation Index (SSCI; Web of Science), Applied Social Sciences Index and Abstracts (ASSIA; ProQuest), International Bibliography of the Social Sciences (IBSS; ProQuest), Sociological Abstracts (ProQuest), Social Services Abstracts (ProQuest), Prospero (Centre for Reviews and Dissemination, University of York), Campbell Collaboration Library of Systematic Reviews (The Campbell Library), Cochrane Library (includes Cochrane Database of Systematic Reviews, Cochrane Central Register of Controlled Trials, Cochrane Methodology Register, Database of Abstracts of Reviews of Effects, Health Technology Assessment Database, NHS Economic Evaluation Database; Wiley), Database of Promoting Health Effectiveness Reviews (DoPHER; EPPI-Centre), Social Care Online (SCIE) and Health Systems Evidence.

A member of the research team (KT) with the help of a trained information scientist developed and implemented the electronic search strategies. All searches were tailored to the specific host site, and are detailed in Additional file 1: Appendix S2. Citation follow up was conducted on the bibliographies of all included articles and relevant previous umbrella reviews. We did not exclude papers on the basis of language, country or publication date. Searches were limited to peer-reviewed publications only. Authors were contacted to obtain any relevant information that was missing. However, if reviews did not have sufficient equity data, they were excluded from further analysis. In addition, experts were contacted to identify any additional relevant information on unpublished and in-progress research from key experts in the field.

### Inclusion and exclusion criteria

The inclusion criteria for the review were determined a priori in terms of PICOS (Population, Intervention, Comparison, Outcome and Setting; [13]):

- Population: Children and adults (all ages) in any high-income country (defined as OECD members), and additional EU-28 members.
- Intervention: Upstream, population-level (e.g. country, state/region/province, municipality), public health policies defined as primary and secondary *level interventions*, in eight *policy domains* (tobacco, alcohol, food and nutrition, reproductive health services, control of infectious diseases, mental health, the environment [road traffic injuries, air, land and water pollution] and workplace regulations) utilising fiscal policy, regulation, education, preventative treatment and screening *delivery mechanisms*.
- Comparison: We included systematic reviews that include studies with and without controls. Acceptable controls include randomised or matched designs.
- Outcomes: Primary outcome measures included inequalities by socio-economic status (SES, defined as: individual income, wealth, education, employment or occupational status, benefit receipt; area-level economic indicators and ethnicity given the strong relationship between ethnicity and lower SES particularly in the USA [14]) in morbidity, mortality, health behaviours, accidents, or injuries. When available, cost effectiveness data was also collected.
- Study design: Systematic reviews of quantitative intervention studies. Following the methods of previous umbrella reviews [15], publications had to meet the two mandatory criteria of Database of Abstracts of Reviews of Effects (DARE): (i) that there is a defined review question (with definition of at least two of, the participants, interventions, outcomes or study designs) and (ii) that the search strategy included at least one named database, in conjunction with either reference checking, hand-searching, citation searching or contact with authors in the field.

### Study selection and data extraction

**Fig. 2:**
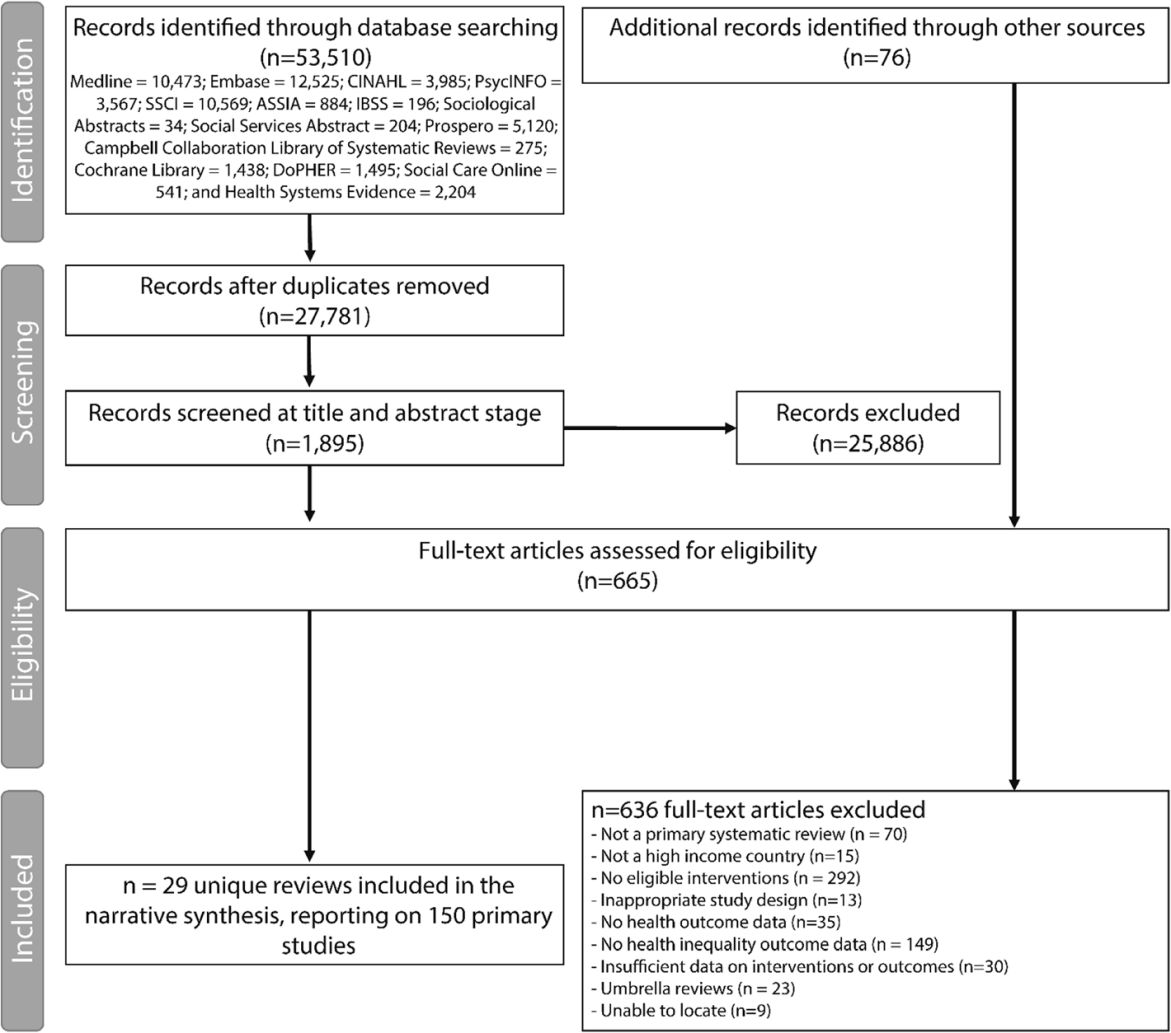
Flow chart of selection procedure

The initial screening of titles and abstracts was conducted by two reviewers (KT and FHB), with a random 10% of the sample checked by a third reviewer (CM). Agreement between the reviewers was excellent (*κ =* 0.77) [16]. The screening of the full papers was conducted by two reviewers (KT and FHB) with input from other members of the research team (AT, TH, CM). The methods and main findings were extracted using a bespoke data extraction form (detailed in Additional file 1: Appendix S3). Data extraction was conducted by four reviewers (CM: food and nutrition; AT: screening and infectious diseases; KT and FHB: all others). A full check of the data extraction was completed by FHB and KT. Any discrepancies on selection and extraction were resolved through discussion between the lead reviewers (KT and FHB) and the project lead (CB). Figure 2 details the process of inclusion and exclusion of studies from the review and the reasons for exclusion at the full paper stage (*n* = 636) are available in Additional file 1: Appendix S4.

### Quality appraisal and data synthesis

The Assessment of Multiple Systematic Reviews (AMSTAR) [17] tool was used to determine the methodological quality of the systematic reviews. We also used the revised version of AMSTAR (R-AMSTAR) [18] which assigns an overall quality score to the systematic review (Additional file 1: Appendix S5). The R-AMSTAR scores were converted to low (11–22), medium (23–33) and high (34–44) quality ratings to aid interpretation. Data extraction only utilised the information from the systematic review (and any relevant supplementary material); we did not extract data from the original primary studies. The systematic reviews were narratively synthesised in accordance with the conceptual framework shown in Fig. 1 [12]. In relation to each domain/intervention, we highlight three sets of public health policy interventions which frame our discussion section: (1) those that are effective in reducing health inequalities; (2) those that do not have any effect on health inequalities or where the systematic review level evidence is unclear; and (3) those interventions that appear to increase health inequalities. In the results and discussion sections that follows, *primary studies* refers to empirical research studies evaluating the impact of a particular intervention. We typically use systematic review (or simply *review*) to highlight the conclusions of a particular systematic review, that often summarise the evidence of primary studies for a particular domain/intervention.

## Results

A total of 53,510 citations were retrieved from the 20 databases searched and downloaded to Endnote. De-deduplication using a combination of Endnote, a web-based software package entitled the ‘Systematic Review Assistant-Deduplication Module’ by Rathbone and colleagues [19] and further manual extraction resulted in 27,781 unique citations. In total, 29 systematic reviews were included in our review (Fig. 2) – reporting on 150 unique primary studies. In terms of the *levels of intervention*, there were 26 reviews focusing on primary prevention [20–45] and three reviews focusing on secondary prevention [46–48]. In respect to the different *delivery mechanisms*, there were five reviews of fiscal policy [21–24, 44], eight of regulation [29, 34–40], six of education [27, 33, 41–43, 45], one of preventative treatment [47] and one of screening [48]. There were eight reviews that also assessed multiple delivery mechanisms [20, 25, 26, 28, 30–32, 46]. In terms of *policy domains*, there were four systematic reviews of tobacco interventions [20, 29, 30, 41] (53 unique relevant primary studies); one review reporting alcohol interventions [44] (one primary study), fourteen reviews of food and nutrition interventions [21–25, 27, 28, 31–36, 42] (63 unique relevant primary studies); three reviews of interventions controlling infectious diseases [26, 46, 47] (21 unique relevant primary studies); three reviews of interventions associated with reproductive health services [43, 45, 48] (8 unique primary references); three reviews of environmental interventions [37–39] (3 relevant unique primary studies) and one of workplace regulations [40] (one unique study). No relevant equality reviews were located for the mental health domain. Only six primary studies were included in more than one systematic review and care was taken not to duplicate their findings. Studies were located in a range of high-income countries, including across the EU-28 members, although as is common, the majority of studies were conducted in the USA. The earliest review was published in 2000 and the latest in 2017. Using the R-AMSTAR tool, nine reviews were considered low quality (score 11–22) [23, 33, 37, 39, 41, 43, 46–48], sixteen medium quality (score 23–33) [20–22, 24–26, 29–32, 34, 35, 40, 42, 44, 45] and four high quality (score 34–44) [27, 28, 36, 38].

The reviews were narratively synthesised below by (i) level of intervention - primary and secondary; (ii) delivery mechanisms - fiscal policy, regulation, education, preventative treatment and screening; and (iii) policy domains - tobacco, food and nutrition, control of infectious diseases, cancer control and prevention, the environment and workplace regulation. The results are also summarised in Tables 1, 2, 3, 4, 5 and 6.

**Table 1:** Summary of included reviews reporting studies of fiscal policy intervention

Study	No. of relevant studies (total studies)	Context (setting, country, search timeframe)	Intervention(s)	Summary of results	AMSTAR quality appraisal (derived from R-AMSTAR)
Brown et al. 2014 [20]	12 (117)	Studies based in a country at stage 4 of the tobacco epidemic or in the WHO European Region, 1995–2013	Tobacco/cigarette (including excise) tax increase, cigarette price increase; population-level cessation support initiatives.	Higher quality evidence suggests a neutral effect of prices increases. Some evidence to suggest that lower SES groups are most likely to change to price-minimising strategies.	28 (medium)
Jackson et al. 2010 [44]	1 (103)	Adults 15 and above, Finland, searches up to 2008	Large reduction in price of alcohol (average 33% decrease).	Alcohol-related deaths increased overall by 16% (95% CI 12.1–19.4). Amongst the 30–59 age group, mortality was highest for those individuals with lower levels of education, social class, or income.	26 (medium)
Alagiyawanna et al. 2015 [21]	2 (17)	No country restrictions, start date to 2013	USA Food Stamp Programme (effects in pregnant women). *Includes 1 study also identified in Black* et al. *(2012).*	Food stamp programs can have positive impacts on low-income populations (decreased probability of gaining insufficient weight during pregnancy) but only small increased infant survival rates in white, low-income women only.	29 (medium)
Black et al. 2012 [22]	9 (14)	High income countries, 1980–2010	USA food subsidy programmes (Special Supplementary Nutrition Program [SNAP] for Women, Infants and Children [WIC]; and Food Stamp Programme). *Includes 1 study also identified in Alagiyawanna* et al. *(2015).*	Authors found limited high quality evidence of the impacts of food subsidy programs on the health and nutrition of adults and children. Improvements in perinatal outcomes was generally limited and most evident in women who smoked during pregnancy.	27 (medium)
de Sa and Lock 2008 [23]	1 (30)	School children aged 4–6 years, UK; start date to August 2007	National School Fruit scheme.	Short-term and long-term increases in fruit and vegetable consumption resulted from the National School Fruit scheme pilot in low SES schools in the UK.	22 (low)
Hillier-Brown et al. 2014 [24]	2 (20)	Deprived homes/population; USA; start date to 2012	USA Food Stamp Programme.	No significant effect on weight change or BMI from Food Stamp intervention overall. Although evidence from the very poorest indicates, there is a significant increase in weight.	31 (medium)
Olsted et al. 2016 [25]	10 (36)	Hungary, Norway, UK, US, and the Netherlands; January 2004–August 2015	Taxes on unhealthy food and drink, free fruit and breakfast school schemes.	Some evidence of taxes on unhealthy food and drink having a positive equity effect on diet outcomes. Free fruit or breakfast schemes in schools had no effect on health inequalities.	29 (medium)
National Collaborating Centre for Women’s and Children’s Health 2009 [26]	2 (142)	Context limited to application to a UK setting; searches to March 2008, articles published before 1988 were excluded. English language only.	National-level approaches to increasing vaccination rates including parent and practitioner incentives.	There is evidence of positive effects on inequalities in vaccination rates for fiscal type interventions.	28 (medium)

**Table 2:** Summary of included reviews reporting studies of regulation policy interventions

Study	No. of relevant studies	Context (setting, country, search timeframe)	Intervention(s)	Summary of results	AMSTAR quality appraisal (derived from R-AMSTAR)
Brown et al. 2014 [20]	14 (117)	Studies based in a country at stage 4 of the tobacco epidemic or in the WHO European Region, 1995–2013	Smoking restrictions in workplaces and enclosed public places; controls on advertising, promotion and marketing of tobacco; neighbourhood improvement initiative. *Includes 3 studies also identified in Fraser* et al. *(2016).*	Higher quality evidence suggests smoking restrictions in workplaces and enclosed public places leads to a widening of inequalities, with a small amount of evidence of a negative effect of a general neighbourhood improvement initiative. Controls on advertising, promotion and marketing appear to have an equal effect across SES groups.	28 (medium)
Frazer et al. 2016 [29]	6 (77)	Cities/States/Countries in New Zealand, Italy, USA; search timeframe – inception to March 2015	Smoking ban in indoor places (e.g. workplaces/bars/ restaurants). *Includes 3 studies also identified in Brown* et al. *(2014).*	Mixed results although some evidence from New Zealand and Italy in particular which suggests a smoking ban may improve health outcomes particularly from those living in deprived areas.	32 (medium)
Thomas et al. 2008 [30]	3 (84)	Variety of settings; OECD countries; inception to January 2006	Smoking restrictions in workplaces and other public places; increased enforcement against underage sales in tobacco; and multifaceted interventions (e.g. combined effects of different anti-tobacco laws).	No evidence of differential effects for smoking restrictions in workplaces.	25 (medium)
Sumar and McLaren 2011 [31]	5 (10)	Women, no country restrictions, 1990-time of study	Introduction of mandatory fortification policy.	Some support is found for the hypothesis that mandatory fortification policy is less likely than information campaigns to lead to worsening inequalities in health by socioeconomic status or race/ethnicity; however, conclusions were complicated by different outcome variables and different economic and political regimes in which interventions took place.	26 (medium)
McGill et al. 2015 [32]	1 (2)	Any age or gender from any country, from 1980 onwards	National salt reduction strategy (whereby manufacturers, retailers, trade associations and the catering sector were committed to salt reduction).	Study based on reformulation of food products found no effect in terms of inequality.	27 (medium)
Hillier-Brown et al. 2017 [34]	3 (30)	City/States in the USA. January 1993 – October 2015	Regulation in a major city to control the trans and saturated fat content of fast-food purchases and mandatory menu labelling.	Mean trans-fat per purchase decreased but no difference by the poverty rate of the neighbourhood in which the restaurant was located. Mixed results for equity effects of menu labelling on calories purchased (negative and neutral).	31 (medium)
Hendry et al. 2015 [35]	1 (14)	New York City; 1980–2012	Trans-fatty acid ban for all licensed food establishments.	Mean trans-fatty acids decreased. Neighbourhood poverty was not associated with trans-fatty acid purchase.	30 (medium)
Olstad et al. 2016 [25]	19(24)	Range of settings including cities, countries and establishments; Korea, UK, USA, France, Finland and Australia, January 2004–August 2015	A range of nutrition policies (e.g. minimum standards, national diabetes prevention), and menu labelling law.	Most nutrition policy interventions showed negative effects on inequalities. Menu labelling had no effect on health inequalities.	28 (medium)
Iheozor-Ejiofor et al. 2015 [36]	3 (155)	Various settings in English areas, start date – 2015	Initiation of water fluoridation.	Although caries and decayed, missing and filled deciduous teeth/surfaces did show improvement following the initiation of water fluoridation, the authors concluded that due to problems with the study designs, results are inconclusive.	36 (high)
Ashton et al. 2009 [37]	1 (24)	More and less deprived cities; UK; 1990–2009	Traffic calming measures documented in two cities.	Significant drop in child pedestrian casualties in more deprived area.	22 (low)
Mulvaney et al. 2015 [38]	1 (21)	City roads, London (UK), various to 2015	The implementation of 20 mph zones.	Study results suggest that 20 mph zones have smaller effects on cycle casualties with increasing levels of social deprivation of the area in which the collision occurred (no evidence demonstrated on adjacent roads). Over the period, the decline in road casualties was greater in less deprived areas despite the 20 mph zones causing socioeconomic inequalities to widen over time.	37 (high)
Benmarhnia et al. 2014 [39]	1 (8)	Specific areas with vulnerable populations, 1980–2013. English language only.	Low emission zones.	Low-emission zones were more beneficial to the wealthiest residents.	21 (low)
Egan et al. 2007 [40]	1 (11)	Privatisation of water industry, UK, 1945–2003	Privatisation of UK’s water industry.	Worsening mental health for clerical and administrative staff (no significant change for manual workers or managers) post intervention. Little change in mean OSI scores for somatic symptoms among any occupation group.	27 (medium)
National Collaborating Centre for Women’s and Children’s Health 2009 [26]	2 (142)	Context limited to application to a UK setting; Searches to March 2008, articles published before 1988 were excluded. English language only.	Mandatory immunisations for school entry.	There is evidence of positive effects on inequalities in vaccination rates for regulatory-style interventions.	28 (medium)

**Table 3:** Summary of included reviews reporting studies of education policy interventions

Study	No. of relevant studies	Context (setting, country, search timeframe)	Intervention(s)	Summary of results	AMSTAR quality appraisal (derived from R-AMSTAR)
Brown et al. 2014 [20]	5 (117)	Studies based in a country at stage 4 of the tobacco epidemic or in the WHO European Region, 1995–2013	Mass media campaigns.	Mixed and inconclusive evidence of health equity effects of mass media campaigns to reduce smoking rates/tobacco use.	28 (medium)
Thomas et al. 2008 [30]	1 (84)	Variety of settings; OECD countries; start date to January 2006	Health warnings on cigarettes.	No evidence of differential effects for the use of health warnings on cigarettes.	25 (medium)
Niederdeppe et al. 2008 [41]	2 (50)	State anti-smoking campaign, USA, 1990 onwards	Anti-smoking campaign run in two USA states.	Unclear effects on smoking behaviour amongst SES groups.	18 (low)
Beauchamp et al. 2014 [42]	2 (14)	National intervention in Frances among children and adults, start date to 2012	Nutrition guidelines for the general population, mass media campaign and obesity screening tools for healthcare professionals.	No effect among low SEP groups and a beneficial effect among high SEP groups in adults, no effect in any SEP group in children.	30 (medium)
Sumar and McLaren 2011 [31]	4 (10)	Women, no country restrictions, 1990-time of study	Information campaigns to increase folate intake. Includes 2 studies also identified in Stockley and Lund (2008).	Information campaigns lead to worsening inequalities in health by socioeconomic status or race/ethnicity.	26 (medium)
Stockley and Lund 2008 [43]	3 (90)	Netherlands, 1989–2006	State initiated national campaign encompassing advertisements in newspapers and national magazines, commercials on television, and radio and posters in healthcare settings. An additional local campaign targeting women in lower socioeconomic groups was also used. *Includes 2 studies also identified in Sumar and McLaren (2011).*	Socioeconomic differences in pre-conception folic acid use widened in the national campaign, but remained similar where the additional local campaign was implemented.	17 (low)
McGill et al. 2015 [32]	1 (36)	Any age or gender from any country, from 1980 onwards	Education campaign to promote healthy eating.	Study based on an education campaign found an overall widening impact.	27 (medium)
Olsted et al. 2016 [25]	1 (36)	Healthy adults or children in any setting or country, January 2004–August 2015	Public information campaign (five a day).	The information campaign (‘5 a day’, UK) had a positive effect on inequalities.	29 (medium)
McLaren et al. 2016 [28]	2 (25)	Males and females, of any age, living, in any geographic region worldwide; database start date to 5 January 2015	Population-level interventions in government jurisdictions for dietary sodium reduction.	Overall, interventions, both education only and education combined with regulation, had little effect on health inequalities with SES inequalities in salt intake persisting over time.	37 (high)
de Silva et al. 2016 [27]	1 (38)	Nurseries, Scotland, 1996-April 2014	Daily supervised toothbrushing in nurseries of 5 year olds. Distribution of fluoridate toothpaste through nurseries to encourage home toothbrushing.	Dental caries dramatically declined during the duration of the national nursery toothbrushing programme. Absolute inequality between dental caries rates in the most deprived areas and those in the least deprived areas was also observed.	34 (high)
Ciliska et al. 2000 [33]	1 (60)	Community, USA, searches from start to August 1998.	Evaluation of the Expanded Food and Nutrition Education Program (EFNEP) comprising education in homes/communities on topics such as nutrition, selecting, buying, cooking and preserving food and safety.	Increase in fruit and vegetable consumption.	22 (low)
Black et al. 2000 [45]	2 (19)	Deprived communities, no restrictions on country, although the majority of the included studies were from the USA, 1989 to 1999.	Health promotion and education interventions to promote the update of cervical screening.	Improved rates of cervical screening amongst deprived communities; cancer incidence was not reported.	28 (medium)

**Table 4:** Summary of included reviews reporting studies of preventative treatment policy interventions

Study	No. of relevant studies	Context (setting, country, search timeframe)	Intervention(s)	Summary of results	AMSTAR quality appraisal (derived from R-AMSTAR)
Croker-Buque et al. 2016 [46]	5 (41)	Children and adolescents, OECD countries, April 2008 – November 2015	Reminder and recall systems.	There is some evidence of positive effects of reminder and recall systems when targeted at disadvantaged groups, but universal systems have no effect on health inequalities.	22 (low)
Menzies and McIntyre 2006 [47]	7 (17)	Indigenous children and adults; search timeframe unknown, Australia, United States, Canada	Funded vaccination (for Hepatitis A and B and pneumococcal disease) for indigenous children and adults, either targeted or part of the universal programs.	Immunisation programs reduce disease in indigenous populations and reduce racial disparities. Vaccinations for viral diseases (e.g. Hepatitis B) is most successful since strain variations are less important and herd immunity is high.	17 (low)

**Table 5:** Summary of included reviews reporting studies of screening policy interventions

Study	No. of relevant studies	Context (setting, country, search timeframe)	Intervention(s)	Summary of results	AMSTAR quality appraisal^a^
Spadea et al. 2010 [48]	5 (29)	Sweden, Italy and US	Screening/Screening.	Population female cancer screening programmes appear to increase screening rates in low SES groups; however the inequality gradient still persists (although does not increase) as screening rates are increased across the whole population.	19 (low)

^a^Derived from R-AMSTAR

**Table 6:** Summary of included reviews reporting studies of multiple policy interventions

Study	No. of relevant studies	Context (setting, country, search timeframe)	Intervention(s)	Summary of results	AMSTAR quality appraisal (derived from R-AMSTAR)
Brown et al. 2014 [20]	4 (117)	Studies based in a country at stage 4 of the tobacco epidemic or in the WHO European Region, 1995–2013	Multiple policies: Smokefree legislation, cigarette tax/price increase, mass media campaign, free NRT, cigarette text warning labels, tobacco advertising ban, youth access law.	Three studies found equal effects of multiple policies across SES groups. One study found that a combination of a smoking ban and two tax increases led to a widening of health inequality.	28 (medium)
McLaren et al. 2016 [28]	2 (25)	Males and females, of any age, living, in any geographic region worldwide; Searches from database start date to 5 January 2015	Population-level interventions in government jurisdictions for dietary sodium reduction.	Interventions combining education campaigns with regulation, had little effect on health inequalities and SES inequalities in salt intake remain.	37 (high)
Croker-Buque et al. 2016 [46]	4 (41)	Children and adolescents, OECD countries, April 2008 – November 2015	Complex interventions incorporating education and enhanced health services.	Complex interventions incorporating education and enhanced health services may be effective in younger children (≤2 years) and boys, when targeted at disadvantaged groups, but there is some evidence of widening health inequalities from universal complex interventions.	22 (low)

### Primary prevention delivery mechanism 1: fiscal

Fiscal strategies employed by the state use the tax system to change demand for products deemed healthy/unhealthy by increasing or decreasing price or rewarding/punishing particular behaviours. Eight reviews of the health inequality effects of fiscal policies in the domains of tobacco (*n* = 1), alcohol (n = 1), food and nutrition (*n* = 5) and the control of infectious diseases (n = 1) were included and the results are summarised below and in Table 1.

#### Tobacco

One large review by Brown et al. [20] explored the effects on health inequalities of population-level interventions and policies to reduce smoking in adults. Twelve studies in the review reported on fiscal measures. These found mixed success in terms of the effects on health inequalities of increasing cigarette or tobacco taxes. However, the higher quality primary studies generally found that smoking or tobacco use reduced in all groups with no strong trends associated with socio-economic status, although one study found particular decreases amongst the lowest educated.

#### Alcohol

A single review by Jackson et al. [44] investigated the effect of fiscal interventions on public health in the domain of alcohol. This systematic review included one primary study that investigated the role of a large reduction in alcohol price in Finland (taxes fell by an average of 33%). It noted an increase in alcohol-related deaths in men by 16% and in women by 31%, with deaths amongst the 30–59 age group greatest for those individuals with lower levels of education, social class and income.

#### Food and nutrition

Five reviews examined the effects on health inequalities of fiscal interventions in the domain of food and nutrition. A review by Alagiyawanna et al. [21] explored the effect of food taxes and subsidies on health behaviours and other health outcomes. Two primary studies included in this review examined effects of the USA Food Stamp Programme (subsidy), finding that it had a positive impacts on fetal survival and weight gain during pregnancy of low-income populations. Food subsidies were also assessed in another review by Black et al. [22] which included nine relevant studies. These predominantly evaluated the Special Supplemental Nutrition Program for Women, Infants and Children in the USA. The review found that participants of food subsidy programs had a 10–20% increased intake of targeted foods or nutrients. Two of the higher quality studies also found a small but significant increase in mean birthweight (23 g – 29 g). Another review by de Sa and Lock [23] examined school fruit and vegetable subsidy programmes. It included a single relevant study which found that the UK’s National School Fruit scheme pilot increased fruit and vegetable intake in the short- and long-term amongst children aged 4–6 years in low-income neighbourhoods who received one free piece of fruit each school day. A review exploring the effectiveness of interventions in reducing inequalities in obesity by Hillier-Brown et al. [24], and contained two relevant studies to our review. These studies from the USA assessed the impact of a nutrition prevention intervention (Food Stamp Programme) on body weight and BMI. The studies had no intervention effect. Another review by Olstad et al. [25] also explored the effects of policies on socioeconomic inequalities in obesity. It contained ten studies relevant to this umbrella review: four studies of taxes on unhealthy foods and drink showed positive equity effects on diet outcomes, but not weight outcomes, whilst six studies of school free fruit or breakfast schemes found no effects on inequalities.

#### Control of infectious diseases

A review by National Collaborating Centre for Women’s and Children’s [26] (commissioned by the National Institute of Health and Clinical Excellence, NICE) reviewed the effectiveness of interventions to reduce differences in the uptake of immunisations in children and young people. It included two studies of fiscal interventions targeting parents and providers. For example, parents were offered incentives in the form of maternity allowance or childcare benefits for ensuring their child immunisations were up to date. Therefore, there is some evidence that incentive schemes may decrease inequalities in vaccination rates.

### Primary prevention delivery mechanism 2: regulation

These interventions were concerned with making and enforcing regulation to encourage/discourage products and services deemed healthy/unhealthy. Fourteen reviews of the effects on health inequalities of regulation in the domains of tobacco (*n* = 3), food and nutrition (*n* = 6), environment (n = 3), workplace (*n* = 1) and the control of infectious diseases (n = 1) were included and the results are summarised below and are described in Table 2.

#### Tobacco

Three reviews investigated the impact of regulatory interventions on tobacco, particularly in relation to workplace, bars and public smoking bans, as well as restrictions on advertising. One review by Brown et al. [20] identified twenty-three relevant studies of regulation policies, while another review by Frazer et al. [29] identified three relevant studies (three studies were included in both reviews). The evidence base was mixed with the higher quality evidence tending to find negative effects on health inequalities of national and state-wide legislation and policies that restricted smoking in workplaces and enclosed public places – with disproportionate gains in the highest groups/areas. Some of the lower quality studies found that smoking bans can have positive effects on health inequalities (e.g. reductions in hospital admissions, particulate matter in bars and smoking prevalence in the most deprived areas and lowest educated groups). The four studies of restrictions on tobacco and cigarette advertising and promotion were more conclusive with equally positive effects on awareness, quit ratios, smoking behaviour, motivation to quit, compliance with legislation across SES levels. A further review by Thomas et al. [30] included two relevant studies and found no evidence of differential health effects (exposure to tobacco smoke, second-hand smoking, cessation) by education or ethnicity of restrictions on smoking in workplaces and public places.

#### Food and nutrition

In terms of food and nutrition, seven reviews investigated how regulatory interventions impacted on health inequalities. One review by Sumar and McLaren [31] assessed the role of mandatory fortification to increase folate intake, and included five relevant studies, which provided mixed support for the effects of mandatory fortification policies. Two studies found a reduction in health inequalities by socioeconomic status or race/ethnicity, while a further three found largely negative effects on absolute health inequalities. Another review by McGill et al. [32] included a study which found that the implementation of a national salt reduction strategy (whereby manufacturers, retailers, trade associations and the catering sector were committed to salt reduction) had no effect on inequalities by social class. Hillier-Brown et al. [34] investigated the role of regulatory interventions to control the fat content of fast food purchases. These studies centred on the municipal restrictions imposed by the New York City Board of Health on the trans and saturated fat content of fast-food purchases in restaurants. At a result of the legislation, food service establishments cannot store or serve food that contains partially hydrogenated vegetable oil and has a total of more than 0.5 g or more trans-fat per serving. In addition, the Board of Health approved a separate measure requiring some restaurants (mostly fast food outlets), to display clearly the caloric content of each menu item on menu boards or near cash registers. The authors found no difference in the mean trans-fat per purchase rate by neighbourhood poverty level as a result of the trans-fatty acid ban. Mixed results were reported for mandatory calorie labelling on menus: one study found greater decreases in calories purchased in areas with residents of higher income and education, while another study found no differential effect on calories purchased by ethnicity. Another review by Hendry et al. [35], which examined the effect of the New York City legislation included one study that explored the effect of restricting the consumption of trans fats between high- and low-poverty neighbourhoods. The study showed there was no difference in trans-fatty acids per purchase between high-and low poverty neighbourhoods. A further review by Olstad et al. [25] provided analysis of a range of nutrition policies, and included minimum standards, particularly in school environments and the menu labelling law in New York City. The results of the 19 studies collated in this review demonstrate negative effects on health inequalities. Finally, another review by Iheozor-Ejiofor [36] investigated the effects of water fluoridation on the prevention of dental caries, and examined the differences by area-level deprivation. It found that fluoridation improved the percentage of children caries-free and reduced the number of decayed, missing and filled deciduous teeth/surfaces in all areas.

#### The environment

Regulating the environment includes measures such as regulating traffic speeds and street design, controlling air, water and land pollution levels. Three reviews were included in our umbrella review; the reviews examined roads (*n* = 2) and air pollution (*n* = 1). Two reviews investigated the role of road and street design-based interventions on casualty numbers. The first review by Ashton et al. [37] assessed the effectiveness of traffic calming measures on reducing unintentional injuries in children. Only one study within this review reported on health inequalities and found there was significant drop in casualties in the more deprived areas, compared to the less deprived areas. The second review by Mulvaney et al. [38] explored how increasing the cycling infrastructure can reduce traffic injuries amongst cyclists: one study included in this review investigated the effects of implementing 20 mph zones on cycle causalities by ethnicity and social deprivation. The study found that although cycle accidents fell as a result of 20 mph zones, overall, the decline in road causalities were greater in more affluent areas compared to more deprived areas. A final systematic review by Benmarhnia et al. [39] exploring air pollution interventions included one relevant primary study that investigated the effects of low emission zones by neighbourhood socioeconomic status. The study showed that, overall, low emission zones improved air quality and had positive effects on standardised mortality rates and years of life gained for all residents, although the benefits of this effect were greater for the wealthiest residents.

#### Workplace

Workplace regulations include health and safety laws, employment rights, as well as legislation around ownership and organisational structures. One systematic review included in our umbrella review by Egan et al. [40] investigated the health effects of the mandatory privatisation of public utilities and industries. This review included just one primary study that reported on health inequality outcomes. This study, which related to the privatisation of the UK’s water industry, found a worsening in mental health among clerical and administrative staff, but no detrimental effects on manual workers or managers thereby having a negative intervention effect.

#### Control of infectious diseases

Infectious diseases are subject to regulation including, for example, mandatory immunisation. A review conducted by the National Collaborating Centre for Women’s and Children’s Health [26] included two relevant studies for our umbrella review, which assessed the effects of requiring proof of immunisation for school entry. One study showed some tentative evidence of improving vaccination rates in disadvantaged groups, while the other study showed much clearer evidence of reducing inequalities in terms of vaccination rates by ethnicity.

### Primary prevention delivery mechanism 3: education

Education, communication and mass media are other policy *delivery mechanisms* available to governments to encourage/discourage products and services deemed healthy/unhealthy. Twelve reviews were included relating to the tobacco (*n* = 3), food and nutrition (*n* = 8) and reproductive health services (*n* = 1) domains. The results are summarised below and are described in Table 3.

#### Tobacco

Three reviews included mass media public health education interventions. One review by Brown et al. [20], exploring the effects of national mass-media (educational) campaigns, included five studies relevant to our umbrella review. These studies examined the impacts of state, or national smoking cessation campaigns in the USA and The Netherlands, and focused on interventions through the television, the radio, and the internet. The results were inconclusive: there was some evidence of widening inequalities, and some evidence of reducing inequalities in terms of smoking quit rates. Another systematic review by Thomas et al. [30] identified one study, which evaluated an education approach to reducing smoking rates (health warnings on packaging). Although the intervention was effective in reducing smoking behaviour overall, there were no consistent differential effects on smoking behaviour by education. The final systematic review by Niederdeppe et al. [41] examined the effects of media campaigns to promote smoking cessation amongst socioeconomically disadvantaged populations. The two key studies in this review related to government campaigns of smoking cessation: one of the studies showed a positive intervention effect amongst low educated women, while the other study had no effect on smoking quit rates or health inequalities.

#### Food and nutrition

Eight systematic reviews investigated the role of mass media and education campaigns in relation to food and nutrition. One review by Beauchamp et al. [42] included two studies relevant to this umbrella review: both studies evaluated the effects of education campaigns on inequalities in obesity prevention. One study showed no intervention effect on inequalities in abdominal adiposity amongst children, whilst another study showed no effect of the intervention on the prevalence of overweight/obesity rates amongst low SES adults, although there was a beneficial effect among higher SES groups. Two studies reported the impact on educational campaigns to increase folate intake (two studies were included in both reviews). One review by Sumar and McLaren [31] included four relevant studies of government-sponsored public information campaigns to increase folate intake. Another review by Stockley and Lund [43] focused on the role of a state-initiated education campaign concerning folic acid for the prevention of birth defects. Three studies describing one intervention) comprised advertisements, commercials and posters, with an additional local campaign targeting low SES women. The authors documented a widening of socio-economic inequalities in pre-conception folic acid use from the national campaign (which persisted for 3 years), however socioeconomic differences were unaffected by the local campaign. The studies showed worsening health inequality effects in terms of folate uptake by education level, and the prevalence of neural tube defects by ethnicity. A review by McGill et al. [32] assessed the effects of public information campaigns to promote healthy eating: one study detailed the French ‘five a day’ health information campaign, which the review authors concluded widened inequalities by education. The systematic review by Olstad et al. [25] identified one relevant study of the UK’s ‘five a day’ campaign that showed positive effects health inequalities in terms of diet outcomes (self-reported fruit and vegetable purchases). A further review by McLaren et al. [28] contained two studies that investigated the effects of education campaigns on dietary sodium reduction: one study – based in Canada – showed that the campaign increased salt intake overall; men with higher incomes were less likely to use table salt compared to men with lower incomes. Another study, based in the USA, showed non-significant changes in salt intake overall, which was reported amongst different ethnic populations. A review by de Silva et al. [27] included one study that reported on the impact of a mandatory national tooth brushing education programme whereby 5-year-old children were supervised daily to brush their teeth (fluoride toothpaste was also distributed for home use). The results of the programme reduced absolute inequalities in terms of dental caries between the most affluent and least affluent areas. Another systematic review, conducted by Ciliska et al. [33], detailed the effectiveness of community interventions to increase fruit and vegetable consumption in children aged 4 and over. Only one state-level intervention was included, this provided an evaluation of the Expanded Food and Nutrition Education Program (EFNEP) – a federal community outreach program. The intervention, which was targeted at low-income families, was shown to increase fruit and vegetable consumption, and therefore had a positive effect on health inequalities.

#### Reproductive health services

A systematic review by Black et al. [45] highlighted how educational campaigns can be used to reduce inequalities in reproductive cancer screening amongst women. Two primary studies in this review found that interventions targeted toward disadvantaged groups increased screening rates – particularly amongst lower socio-economic groups.

### Secondary prevention delivery mechanism 1: preventative treatment

These interventions were concerned with increasing the uptake of preventative health care services. Two reviews of the effects of preventative treatment on health inequalities in the domain of infectious disease control were included in our umbrella review; the results are summarised below and are described in Table 4.

#### Infectious disease control

A review conducted by Crocker-Buque et al. [46] examined the effectiveness of interventions to reduce inequalities in vaccine uptake in children and adolescents. Four studies included in this review provided some evidence of positive effects of ‘reminder and recall’ systems when targeted at disadvantaged groups, but universal systems had no effect on reducing inequalities in vaccine uptake rates. An additional review by Menzies and McIntyre [47] examined vaccine preventable diseases and vaccination policy for indigenous populations. Their review on the impact of vaccination policies for indigenous adults and children comprising seven studies. The review showed that a combination of targeted and universal immunisations improved health outcomes for indigenous populations; improvements using this approach were particularly evident for viral diseases (e.g. Hepatitis B). However, the systematic review concluded that universal interventions for children appear to widen inequalities between indigenous and nonindigenous populations for pneumococcal diseases.

### Secondary prevention delivery mechanism 2: screening

Screening involves offering age-appropriate population-level testing for certain diseases. One review showed the impact of interventions on cancer control and prevention (*n* = 1); this is summarised below, and are described in Table 5.

#### Reproductive health services

A review of interventions to improve attendance in sexual cancer screening among lower socioeconomic women by Spadea et al. [48] included five primary studies that explored the effectiveness of screening programmes. The studies all showed that population wide programmes increased screening rates across all socio-economic groups; there was no differential effects by SES.

### Primary and secondary prevention: multiple intervention studies

Three systematic reviews also included studies that involved multiple types of policy mechanisms simultaneously. These results are summarised below and are described in Table 6. The smoking review by Brown et al. [20] included four studies on the effects of multiple policies, including a combination of fiscal, regulation and education approaches. The effects of these approaches were generally equal across socio-economic groups, with the exception of one primary study that found a combination of a smoking ban and two tax increases reduced smoking in those in paid employment, but had no effect amongst those without paid work. The food and nutrition review by McLaren et al. [28] included two primary studies investigating the effects of education campaigns and nutritional labelling in combination with an intervention directed towards lowering the salt content in food products. No statistically significant changes were noted between socio-economic groups so the policy had a neutral health inequalities effect. The control of infectious diseases systematic review by Crocker-Burque et al. [46] included five primary studies detailing a complex intervention, which incorporated both education and preventative health services. The study showed some mixed evidence by age group, however there is evidence that targeted interventions were effective in encouraging childhood vaccination when specifically targeted at lower SES groups of younger children (≤2 years) and especially boys.

## Discussion

### Effects of public health policies on health inequalities

Twenty-nine systemic reviews were included in this umbrella review, comprising 150 unique primary studies. This is a large increase on the seven studies located by Lorenc and colleagues [49] in a previous umbrella review published in 2013. Following our framework for how the welfare state can influence health inequalities through public health policies, we grouped interventions in terms of primary and secondary prevention levels; working through five policy mechanisms (fiscal policy, regulation, education, preventative treatment and screening) and within eight policy domains (tobacco, alcohol, food and nutrition, reproductive health services, the control of infectious diseases, mental health, the environment and workplace regulations). The distribution of systematic reviews across our framework is shown in Table 7. There was considerable heterogeneity in terms of volume of evidence available on the different types of interventions, between the nature of the interventions, and between the primary studies within each of the systematic reviews examined. As a result of these issues, and the paucity of repeated evaluations of the same interventions, it is only possible to make a cautious assessment of the overall effectiveness of public health policies in reducing health inequalities. However, when examining the review as a whole, a tentative pattern emerges and it is possible to identify (1) public health policy interventions that are effective in reducing health inequalities; (2) those that do not have any effect on health inequalities or where the systematic review level evidence is unclear; and (3) those interventions that appear to increase health inequalities.

**Table 7:**
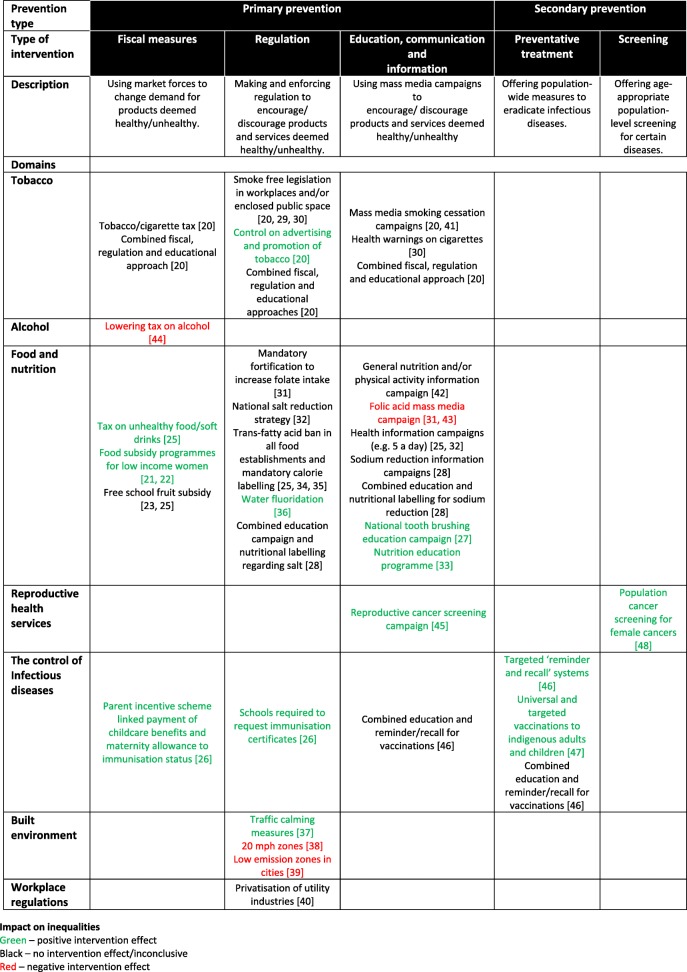
Matrix of population-level preventative public health interventions

In terms of public health policy interventions for reducing health inequalities, there is evidence of effectiveness of this welfare state approach from across all five of our policy mechanisms: fiscal, regulatory, education, preventative treatment and screening. In terms of fiscal approaches, this umbrella review has identified systematic review level evidence to suggest that taxes on unhealthy food and drinks, food subsidy programmes for low-SES women, and fiscal incentive schemes for childhood vaccinations are effective in reducing health inequalities – largely by improving the health or health behaviours of the most vulnerable. In terms of regulatory interventions, we have identified review level evidence of the effectiveness of controlling tobacco advertising, water fluoridisation, requiring proof of immunisation for school entry (to increase vaccination rates amongst the lowest SES groups), and regulating traffic speeds to reduce SES inequalities in child accidents (but not cycling accidents). In terms of mass education interventions, a national tooth brushing education programme was found to be effective in improving dental hygiene amongst children from lower SES backgrounds and a nutrition programme, targeted at low-income families, was shown to increase fruit and vegetable consumption. Reproductive cancer screening information campaigns were also demonstrated to decrease health inequalities. Concerning preventative treatment, universal and targeted vaccinations to indigenous and disadvantaged youth were effective in improving vaccination uptake. In terms of screening, there was evidence that population-wide programmes increased screening rates for reproductive cancers across all socio-economic groups.

There is also evidence though of public health policies, whilst effective in terms of improving overall population health have no effect on health inequalities - either from a positive or negative viewpoint. The systematic review level evidence synthesised in this review suggests fiscal interventions that have no overall effect on health inequalities include tobacco taxes and free fruit provision in schools. Regulatory interventions with no effect on health inequalities include mandatory fortification to increase folate intake; legislative salt reduction; and trans-fat ban and calorie labelling in restaurants. Educational interventions found to have limited effect on health inequalities included smoking cessation campaigns, health information campaigns and the promotion of childhood vaccinations through the media. For some policy areas, the evidence base was small or uncertain. This included the role of some regulation interventions – specifically smoking bans, and the effects of the privatisation of industries on occupational health inequalities as well as some education campaigns – specifically in regards to campaigns to reduce smoking. These interventions need further investigation to ascertain why they are positive for overall population health, but yet are ineffective in terms of reducing inequalities in health.

Thirdly, this umbrella review has identified a smaller set of public health policies that systematic review level evidence suggests could increase health inequalities – potentially leading to so-called ‘intervention generated inequalities’ [49]. Lowering alcohol tax by 33% was shown to increase inequalities in mortality amongst low SES groups in Finland. In terms of regulation policy mechanisms, some of the environmental interventions (20 mph zones and low emission zones) increased inequalities in cycle accidents and mortality between more and less deprived neighbourhoods. Some education interventions also had mixed results, specifically health eating programmes campaigns to increase folate intake.

### State of the evidence base

The review aimed to identify systematic review literature addressing health inequalities in population-based public health interventions. The study is robust, and comprehensive and the systematic reviews presented above demonstrate the state of the research literature to date in this field. Compared to the volume of material investigating overall health outcomes across all public health domains, very few relevant reviews have been conducted which examine health inequalities, with the majority of included studies coming from reviews that looked at general health effects and only had health inequality effects as a secondary health outcome. Whilst this may be the result of the systematic reviews failing to report all relevant published subgroup outcomes, it is more likely to reflect the primary study evidence base, particularly the failing to report by PROGRESS-PLUS. In the protocol, we detailed that cost-effectiveness data would be collected, if available. Unfortunately, none of the systematic reviews included in this umbrella review included data about cost-effectiveness. Therefore, whilst we have been able to identify some potentially effective interventions, the cost-effectiveness of these interventions remains unclear.

Many of the primary studies were conducted in the USA – this is in keeping with other reviews and results from the higher number of evaluations conducted in the USA. However, the wider welfare system in the United States is different than other liberal regimes (e.g. the UK, Ireland, Canada and Australia) – with lower levels of benefits, public health regulation and health care access [50, 51] - and also fundamentally different than the conservative, social democratic and Southern welfare systems operational in the rest of Europe [5]. Recognising that the welfare system is an important contextual determinant of health, we must acknowledge that the effective interventions found in this umbrella review may not easily be transferable from one country/system context to another [52]. For example, in the US, the Supplemental Nutrition Assistance Program (SNAP; formally known as the Food Stamp Program) is a federal aid program for low-income households providing food assistance. However, in the UK and other European countries, targeted food assistance is not provided, as instead income protection in the form of cash benefits (such as Income Support or more recently Universal Credit) is used to maintain overall income. Interventions are not conducted in a policy vacuum but form part of a wider, complex system [53]. Similarly, interventions from the UK or other European countries might not be effective if transferred in a standalone way to the US context.

There are two aspects to the quality of evidence in this umbrella review - the quality of the included systematic reviews and of the primary studies included therein. Many of the systematic reviews failed to adequately describe their methods. The use of R-AMSTAR highlighted that only four of the reviews were classed as ‘high’ quality. A third of the reviews were deemed ‘low’ in quality having not provided sufficient detail of their methods and/or the studies with the remaining reviews rated as ‘medium’. Future reviews should more consistently and transparently describe their methodologies using a standardised approach, such as PRISMA [54]. In terms of assessing the quality of the primary studies included in the reviews, a variety of tools were utilised: some used validated measures of quality, while others reviews did not; further, some reviews did not report on the quality of included studies. Many of the underpinning primary studies were considered to be weak in quality, which appeared to be largely due to the reliance on observational study designs. Given the nature of state-led population-level interventions that this review sought to identify, the ‘gold standard’ randomised control trials were absent, and typically repeat cross-sectional studies, or interrupted time series were the most robust study design which were available. The systematic review authors used a variety of different criteria or toolkits to assess the quality of the underlying primary studies. Whilst some were graded ‘high’ and ‘moderate’, the majority of studies were identified as ‘low’ in quality.

There are also some significant evidence gaps that this umbrella review has highlighted. The majority of evidence that this review presents is drawn from tobacco and food/nutrition domains, followed by control of infectious diseases. In terms of intervention types that were the dominant, most evidence is for regulation and education policy mechanisms. In addition, there is a small amount of evidence for the environment, reproductive health and workplace interventions. No reviews met our criteria for mental health, and a single review investigated the impact on health inequalities in alcohol interventions. There was also no data presented on the cost effectiveness of interventions. So, there are some clear evidence gaps within the health inequality literature regarding the effects of public health policy interventions that could be explored by future research. For example, pressing policy questions such as the effectiveness of alcohol-related policy changes on inequalities in alcohol consumption and alcohol-related harm [55].

Further, many of the effective interventions identified in this umbrella review were targeted ones – focusing resource on those most at risk (e.g. the lowest SES groups). This is one way of conceptualising health inequalities – as the health of the most disadvantaged (targeted approach). However, health inequalities can also be conceptualised in terms of the gap between the least and most disadvantaged (gap approach) or as the entire social gradient in health – which reaches from the bottom to the top of society (gradient approach [56]). However, only a few studies in this review took a ‘gap’ approach and none took a ‘gradient’ approach. The wider health inequalities literature suggests that a gradient approach (using universal interventions – perhaps proportionately applied to the most in need) are likely to be the most effective in terms of reducing health inequalities across the whole population [57]. Targeted interventions, by their very nature, are only able to raise the health of those targeted, and so do little for those in the middle of the health inequalities spectrum.

### Implications for research and practice

There is ongoing interest in public health practice and policy making environments to reduce health inequalities through action on the social determinants of health. This umbrella review has provided a synthesis of evidence relating to the effectiveness of a specific welfare state policy arena – public health policy - where policymakers can intervene. It has therefore overcome problems with the existing research on the effects of welfare states on health inequalities as it goes beyond the ‘black box’ of associational studies (that examine the social determinants of health and health inequalities by welfare state type) by looking at the effects of specific interventions that could in turn be implemented into practice [6]. This review however, also highlights the complexities involved in developing and evaluating population-level public health policy interventions to reduce health inequalities – not least in regards to the transferability of interventions across different contexts and the fact that interventions which might improve overall population health are not necessarily always successful in simultaneously reducing health inequalities [58].

Nonetheless, some potentially promising interventions have been identified that policymakers could consider implementing – in the context of simultaneous evaluation. This includes food subsidy programmes; taxes on unhealthy food and drinks; fiscal incentive schemes; proof of immunisation; control on tobacco advertising; targeted vaccinations; regulating traffic speeds; oral health and some nutritional education programmes; and some population and targeted screening interventions. However, there is also some evidence from this review and elsewhere [59, 60], that some strategies may actually worsen inequalities: leading to so-called ‘intervention-generated inequalities’ [49]. Our review highlights some of these such as lowering the tax on alcohol; 20 mph zones and low emission zones; as well as some education interventions specifically in regards to increasing folate intakes. Policy makers and commissioners should be cautious in implementing such approaches until further evaluations are conducted and the effects on health inequalities are more fully understood. Likewise, there are some policies where our review suggests that the evidence base is mixed, conflicting, absent or unclear. Further research is needed in these areas in order to ensure effective evidence based policy. The priority areas for such future research include alcohol related policies; mental health policies; smoking bans; calorie labelling; and workplace regulation. Future reviews and primary evaluations of the effects of public health policies on health inequalities should also include cost effectiveness data and apply suitable quality appraisal techniques. More widely, the public health research community should start to more thoroughly apply an equity lens to evaluations – looking at differences in intervention effects by, for example, SES [61].

The focus for this review of systematic reviews has been upstream, population level public health policies. We considered all state-level interventions whether they focused on policies instigated by central government (e.g. raising the price of tobacco) or devolved to municipalities (e.g. trans-fat ban in New York City). In reality, the distinction between policies organised centrally or regionally has changed over time and is specific to individual countries. For example, in the Nordic States from the mid-1990s, municipalities have had a dual role of both implementing national policy goals and deciding how to prioritise their funding according to local opinion [62]. In the UK however, the shift in power to local governments came later in response to the Health and Social Care Act 2012 which reconfigured the structure of public health (see [63] for further details). Consequently, the primary reviews that underpin the systematic reviews are often time and context specific. Due to the low numbers of reviews for each of the separate domains/interventions (and the large proportion of reviews that reported mixed effects), it was not possible to ascertain whether the time of publication influenced the success of an intervention. The transferability of successful interventions to other welfare states with differing modes of delivery is complex also. Implementation and enforcement would be a driver of success or otherwise, and often the level of detail provided by the systematic review was inadequate to ascertain the specifics of how an intervention was executed. Going forward, future primary studies and subsequent systematic reviews should provide clear evidence of the precise nature of interventions, including details of how the intervention was delivered and whether additional administration was required to ensure compliance [64].

### Strengths and limitations

There are several strengths of this umbrella review presented. We believe our methodological approach to this umbrella review was robust: our search was broad and wide-ranging, which included an inclusive database and grey literature search with no details of specific interventions, or health inequality terminology, which would otherwise limit the search. In addition, no language or date restrictions were applied to our search strategy. Consequently, the reviews presented here, and the list of primary studies which they report detail the health inequality effects for the majority of the relevant studies available at the time of our search. This review of existing systematic reviews pooled the best available review level evidence of high-level (national and state-wide) policy interventions (no small scale ‘policy’ interventions e.g. single school, workplace). These are likely to represent the most effective strategies that governments have employed to address health inequalities, and could be used as template to improve health amongst some of the poorest and deprived people in high-income countries.

However, there are also several limitations to our umbrella review. We deviated from our published protocol in two areas. Due to the larger than expected number of hits, the review focuses only on socio-economic inequalities in health not population health in general. In addition, we used the Assessment of Multiple Systematic Reviews (AMSTAR) [17] to conduct quality appraisal and not GRADE [65] as described in the protocol in keeping with best practice. Furthermore, a major limitation of the final included reviews was their design, as many did not assess the quality of the included primary studies. Further, a high number of reviews of potentially relevant policy interventions were excluded because they failed to report outcomes by SES or ethnicity. We would recommend that future primary studies should follow guidance by PROGRESS-PLUS and report how health outcomes for specific interventions differ by subgroup. Many potentially relevant studies to this work were excluded on the basis of insufficient health or SES outcome data. Like all umbrella reviews, we have only synthesised the results of systematic reviews and the relevant primary studies included within them. It is very likely that in a number of policy domains, additional primary evaluations have been conducted either after the systematic reviews have been completed, or perhaps they did not fit the criteria for inclusion in the systematic reviews. Furthermore, it is possible that there is publication bias (that negative results are less likely to be published) with regards to the primary studies. Positive intervention effects in primary studies are compounded in systematic reviews and umbrella reviews as the primary study evidence base may be skewed. This umbrella review is, therefore, a synthesis of the results of systematic reviews not a synthesis of all primary evaluations of such interventions.

## Conclusion

Understanding the role of the welfare state in reducing health inequalities is a longstanding theme within comparative public health research. However, the majority of work has examined general associations between welfare state types and health inequalities. There has been very little research examining the effects of specific welfare state policies on health inequalities. This umbrella review has sought to fill this gap by investigating how welfare states influence the social determinants of health inequalities institutionally through specific public health policies – examining both primary prevention (fiscal, regulation and education) and secondary prevention (preventative treatment and screening) interventions. The evidence from this review though suggests that some public health policies – using fiscal, regulatory, education, preventative treatment or screening mechanisms - may be effective in improving health inequalities. Key examples include: taxes on unhealthy food and drinks; food subsidy programmes for low-income families; fiscal incentive schemes liked to immunisation status; proof of immunisation for school admission; tobacco advertising control measures; traffic calming measures; oral health (water fluoridation and tooth brushing campaign); some nutritional and cancer education programmes; universal and targeted vaccinations for indigenous populations; and targeted and population screening interventions. However, we also found evidence of policies that were ineffective and some that actually increased health inequalities, such as lowering tax on alcohol; 20 mph zones and low emission zones; as well as some education interventions specifically campaigns aimed at increasing folate intakes. Therefore, our umbrella review has found evidence that suggested thirteen interventions can reduce health inequalities but also that in some cases they might increase them. As the evidence base reviewed here is on the small side and of varying quality, there is a need for further evaluations - particularly evaluations that undertake sub-group analysis by SES and which assess cost-effectiveness. There is also a need for systematic reviews in public health to assess the effects of interventions on health inequalities.

## Declarations

*AMSTAR:* A MeaSurement Tool to Assess systematic Reviews. Used to evaluate the quality of systematic reviews.

*R-AMSTAR*: R(evised)-AMSTAR. Used to quantify the quality by assigning a quality score to each systematic review.

*DARE:* Database of Abstracts of Reviews of Effects.

*EU-28:* European Union member countries.

*GRADE*: Grading of Recommendations, Assessment, Development and Evaluations. GRADE is used as a formal process to rate the quality of the scientific evidence in a systematic review.

*PICOS*: Criteria for inclusion and exclusion of studies.

*PRISMA:* Preferred Reporting Items of Systematic Reviews and Meta-Analyses.

PROGRESS-Plus: Acronym to identify population and individual characteristics across which health inequities may exist.

*PROSPERO*: International prospective register of systematic reviews.

*SES*: Socioeconomic status is the social standing or class of an individual or group. It is often measured by determining education, income, occupation, or a composite of these dimensions.

## Additional file

Additional file 1: **Appendix S1.** PRISMA checklist. **Appendix S2.** Search strategy. **Appendix S3.** Example data extraction form. **Appendix S4.** AMSTAR rating for all included studies. **Appendix S5.** Reasons for exclusion of full-text articles. (PDF 1014 kb)
