# Supplementary material for: The effects of public health policies on health inequalities in high-income countries: an umbrella review
Source: BMC Public Health. 2018 Jul 13;18:869. doi: 10.1186/s12889-018-5677-1 (PMC6044092; doi:10.1186/s12889-018-5677-1)
Supplement: Supplementary file 1 — Appendix S1. PRISMA checklist. Appendix S2. Search strategy. Appendix S3. Example data extraction form. Appendix S4. AMSTAR rating for all included studies. Appendix S5. Reasons for exclusion of full-text articles. (PDF 1014 kb) [file 12889_2018_5677_MOESM1_ESM.pdf]

Title:

The effects of public health policies on health inequalities in high-income countries: an umbrella review

Appendices:

Appendix S1: PRISMA checklist

Appendix S2: Search strategy

Appendix S3: Example data extraction form

Appendix S4: AMSTAR rating for all included studies

Appendix S5: Reasons for exclusion of full-text articles

Appendix S1: PRISMA 2009 checklist

Section/topic	#	Checklist item	Reported on line #
**TITLE:**<br>The effects of public health policies on health inequalities in high-income countries: an umbrella review			
Title	1	Identify the report as a systematic review, meta-analysis, or both.	31-32
**ABSTRACT**			
Structured summary	2	Provide a structured summary including, as applicable: background; objectives; data sources; study eligibility criteria, participants, and interventions; study appraisal and synthesis methods; results; limitations; conclusions and implications of key findings; systematic review registration number.	24-45
**INTRODUCTION**			
Rationale	3	Describe the rationale for the review in the context of what is already known.	62-79
Objectives	4	Provide an explicit statement of questions being addressed with reference to participants, interventions, comparisons, outcomes, and study design (PICOS).	128-151
**METHODS**			
Protocol and registration	5	Indicate if a review protocol exists, if and where it can be accessed (e.g., Web address), and, if available, provide registration information including registration number.	97-99
Eligibility criteria	6	Specify study characteristics (e.g., PICOS, length of follow-up) and report characteristics (e.g., years considered, language, publication status) used as criteria for eligibility, giving rationale.	103-151
Information sources	7	Describe all information sources (e.g., databases with dates of coverage, contact with study authors to identify additional studies) in the search and date last searched.	103-113
Search	8	Present full electronic search strategy for at least one database, including any limits used, such that it could be repeated.	Appendix S2
Study selection	9	State the process for selecting studies (i.e., screening, eligibility, included in systematic review, and, if applicable, included in the meta-analysis).	154-163<br>Figure 2
Data collection process	10	Describe method of data extraction from reports (e.g., piloted forms, independently, in duplicate) and any processes for obtaining and confirming data from investigators.	156-163

Data items	11	List and define all variables for which data were sought (e.g., PICOS, funding sources) and any assumptions and simplifications made.	Appendix S3
Risk of bias in individual studies	12	Describe methods used for assessing risk of bias of individual studies (including specification of whether this was done at the study or outcome level), and how this information is to be used in any data synthesis.	168-181
Summary measures	13	State the principal summary measures (e.g., risk ratio, difference in means).	140-144
Synthesis of results	14	Describe the methods of handling data and combining results of studies, if done, including measures of consistency (e.g., I^2) for each meta-analysis.	NA
Risk of bias across studies	15	Specify any assessment of risk of bias that may affect the cumulative evidence (e.g., publication bias, selective reporting within studies).	681-684
Additional analyses	16	Describe methods of additional analyses (e.g., sensitivity or subgroup analyses, meta-regression), if done, indicating which were pre-specified.	NA
**RESULTS**			
Study selection	17	Give numbers of studies screened, assessed for eligibility, and included in the review, with reasons for exclusions at each stage, ideally with a flow diagram.	Figure 1
Study characteristics	18	For each study, present characteristics for which data were extracted (e.g., study size, PICOS, follow-up period) and provide the citations.	Tables 1-6
Risk of bias within studies	19	Present data on risk of bias of each study and, if available, any outcome level assessment (see item 12).	Appendix S4
Results of individual studies	20	For all outcomes considered (benefits or harms), present, for each study: (a) simple summary data for each intervention group (b) effect estimates and confidence intervals, ideally with a forest plot.	Tables 1-6
Synthesis of results	21	Present results of each meta-analysis done, including confidence intervals and measures of consistency.	NA
Risk of bias across studies	22	Present results of any assessment of risk of bias across studies (see Item 15).	NA
Additional analysis	23	Give results of additional analyses, if done (e.g., sensitivity or subgroup analyses, meta-regression [see Item 16]).	NA
**DISCUSSION**			
Summary of evidence	24	Summarize the main findings including the strength of evidence for each main outcome; consider their relevance to key groups (e.g., healthcare providers, users, and policy makers).	475-651
Limitations	25	Discuss limitations at study and outcome level (e.g., risk of bias), and at review-level (e.g., incomplete retrieval of identified research, reporting bias).	655-685

Conclusions	26	Provide a general interpretation of the results in the context of other evidence, and implications for future research.	689-709
**FUNDING**			
Funding	27	Describe sources of funding for the systematic review and other support (e.g., supply of data); role of funders for the systematic review.	745-747

From: Moher D, Liberati A, Tetzlaff J, Altman DG, The PRISMA Group (2009). Preferred Reporting Items for Systematic Reviews and Meta-Analyses: The PRISMA Statement. PLoS Med 6(7): e1000097. doi:10.1371/journal.pmed1000097

Appendix S2: Search strategy

Medline

1 search*.tw.
2 (systematic adj2 (review* or overview*)).mp. or Systematic Review/
3 (meta analy* or metaanaly*).mp. or Meta Analysis/
4 (umbrella adj2 review).tw.
5 "review of reviews".tw.
6 1 or 2 or 3 or 4 or 5
7 (("population level" or "population based" or "population orientated" or "population oriented" or "community level" or "community based" or "community orientated" or "community oriented") adj8 (intervention$ or prevention or policy or policies or program$ or project$)).tw.
8 (health adj8 (intervention$ or prevention or policy or policies or program$ or project$)).tw.
9 7 or 8
10 6 and 9
11 animals/
12 humans/
13 11 not (11 and 12)
14 10 not 13

Embase

1 search*.tw.
2 (systematic adj2 (review* or overview*)).mp. or Systematic Review/
3 (meta analy* or metaanaly*).mp. or Meta Analysis/
4 (umbrella adj2 review).tw.
5 "review of reviews".tw.
6 1 or 2 or 3 or 4 or 5
7 (("population level" or "population based" or "population orientated" or "population oriented" or "community level" or "community based" or "community orientated" or "community oriented") adj8 (intervention$ or prevention or policy or policies or program$ or project$)).tw.
8 (health adj8 (intervention$ or prevention or policy or policies or program$ or project$)).tw.
9 7 or 8
10 6 and 9
11 animals/
12 humans/
13 11 not (11 and 12)
14 10 not 13

Cumulative Index to Nursing and Allied Health Literature (CINAHL; EBSCOhost)

1 AB search* OR TI search*
2 AB(systematic N2 (review* or overview*)) OR TI(systematic N2 (review* or overview*)) OR PT(Systematic Review)
3 AB(meta analy*) OR TI(meta analy*) OR AB(metaanaly*) OR TI(metaanaly*) OR PT(Meta Analysis)
4 AB(umbrella N2 review) OR TI(umbrella N2 review)
5 AB("review of reviews") OR TI("review of reviews")
6 S1 or S2 or S3 or S4 or S5
7 AB(("population level" or "population based" or "population orientated" or "population oriented" or "community level" or "community based" or "community orientated" or "community oriented") N8 (intervention* or prevention or policy or policies or program* or project*)) OR TI(("population level" or "population based" or "population orientated" or "population oriented" or "community level" or "community based" or "community orientated" or "community oriented") N8 (intervention* or prevention or policy or policies or program* or project*))

8 AB(health N8 (intervention* or prevention or policy or policies or program* or project*)) OR TI(health N8 (intervention* or prevention or policy or policies or program* or project*))
9 S7 OR S8
10 S6 AND S9

PsycINFO (EBSCOhost)

1 AB search* OR TI search*
2 AB (systematic N2 (review* or overview*)) OR TI (systematic N2 (review* or overview*)) OR SU (Systematic Review)
3 AB(meta analy*) OR TI (meta analy*) OR AB(metaanaly*) OR TI (metaanaly*) OR DE (Meta Analysis)
4 AB (umbrella N2 review) OR TI(umbrella N2 review)
5 AB("review of reviews") OR TI("review of reviews")
6 S1 or S2 or S3 or S4 or S5
7 AB(("population level" or "population based" or "population orientated" or "population oriented" or "community level" or "community based" or "community orientated" or "community oriented") N8 (intervention* or prevention or policy or policies or program* or project*)) OR TI(("population level" or "population based" or "population orientated" or "population oriented" or "community level" or "community based" or "community orientated" or "community oriented") N8 (intervention* or prevention or policy or policies or program* or project*))
8 AB(health N8 (intervention* or prevention or policy or policies or program* or project*)) OR TI(health N8 (intervention* or prevention or policy or policies or program* or project*))
9 S7 OR S8
10 S6 AND S9

Applied Social Sciences Index and Abstracts (ASSIA; ProQuest)

1 ab,ti(search*)
2 ab,ti(systematic NEAR/2 (review* or overview*)) OR SU.EXACT("Systematic reviews")
3 ab,ti(meta analy*) OR ab,ti(metaanaly*) OR SU.EXACT("Meta-analysis")
4 ab,ti(umbrella NEAR/2 review)
5 ab,ti("review of reviews")
6 1 OR 2 OR 3 OR 4 OR 5
7 ab,ti(("population level" or "population based" or "population orientated" or "population oriented" or "community level" or "community based" or "community orientated" or "community oriented") NEAR/8 (intervention* or prevention or policy or policies or program* or project*))
8 ab,ti(health NEAR/8 (intervention* or prevention or policy or policies or program* or project*))
9 7 OR 8
10 6 AND 9

International Bibliography of the Social Sciences (IBSS; ProQuest)

1 ab,ti(search*)
2 ab,ti(systematic NEAR/2 (review* or overview*))
3 ab,ti(meta analy*) OR ab,ti(metaanaly*)
4 ab,ti(umbrella NEAR/2 review)
5 ab,ti("review of reviews")
6 1 OR 2 OR 3 OR 4 OR 5
7 ab,ti(("population level" or "population based" or "population orientated" or "population oriented" or "community level" or "community based" or "community orientated" or "community oriented") NEAR/8 (intervention* or prevention or policy or policies or program* or project*))
8 ab,ti(health NEAR/8 (intervention* or prevention or policy or policies or program* or project*))
9 7 OR 8
10 6 AND 9

Sociological abstracts

1 ab,ti(search*)
2 ab,ti(systematic NEAR/2 (review* or overview*))
3 ab,ti(meta analy*) OR ab,ti(metaanaly*)
4 ab,ti(umbrella NEAR/2 review)
5 ab,ti("review of reviews")
6 1 OR 2 OR 3 OR 4 OR 5
7 ab,ti(("population level" or "population based" or "population orientated" or "population oriented" or "community level" or "community based" or "community orientated" or "community oriented") NEAR/8 (intervention* or prevention or policy or policies or program* or project*))
8 ab,ti(health NEAR/8 (intervention* or prevention or policy or policies or program* or project*))
9 7 OR 8
10 6 AND 9

Social Services Abstracts (ProQuest)

1 ab,ti(search*)
2 ab,ti(systematic NEAR/2 (review* or overview*))
3 ab,ti(meta analy*) OR ab,ti(metaanaly*)
4 ab,ti(umbrella NEAR/2 review)
5 ab,ti("review of reviews")
6 1 OR 2 OR 3 OR 4 OR 5
7 ab,ti(("population level" or "population based" or "population orientated" or "population oriented" or "community level" or "community based" or "community orientated" or "community oriented") NEAR/8 (intervention* or prevention or policy or policies or program* or project*))
8 ab,ti(health NEAR/8 (intervention* or prevention or policy or policies or program* or project*))
9 7 OR 8
10 6 AND 9

Prospero

1 population – **ALL FIELDS**
2 intervention – **ALL FIELDS**
3 health – **ALL FIELDS**
3 1 AND 2 AND 3

The Campbell Library

1 (population level or population based or population orientated or population oriented or community level or community based or community orientated or community oriented) AND (intervention* or prevention or policy or policies or program* or project*) – ALL TEXT
2 health AND (intervention* or prevention or policy or policies or program* or project*) – ALL TEXT
3 1 OR 2

Cochrane Library

1 (search*):ti,ab
2 (systematic near/2 (review* or overview*)):ti,ab
3 meta next analy* or metaanaly*:ti,ab
4 MeSH descriptor: [Meta-Analysis as Topic] explode all trees
5 MeSH descriptor: [Meta-Analysis] explode all trees
6 (umbrella near/2 review):ti,ab
7 ("review of reviews"):ti,ab
8 {or #1-#7}
9 (("population level" or "population based" or "population orientated" or "population oriented" or

"community level" or "community based" or "community orientated" or "community oriented") near/8 (intervention* or prevention or policy or policies or program* or project*)):ti,ab
10 (health near/8 (intervention* or prevention or policy or policies or program* or project*)):ti,ab
11 {or #9-#10}
12 #8 and #11

Social Science Citation Index (Web of Science)
1 TS=search*
2 TS=(meta analy*) OR TS=metaanaly*
3 TS=(systematic NEAR/2 (review* or overview*))
4 TS=(umbrella NEAR/2 review)
5 TS=("review of reviews")
6 #1 OR #2 OR #3 OR #4 OR #5
7 TS=((("population level") or ("population based") or ("population orientated") or ("population oriented") or ("community level") or ("community based") or ("community orientated") or ("community oriented")) NEAR/8 ((intervention*) or (prevention) or (policy) or (policies) or (program*) or (project*)))
8 TS=(health NEAR/8 (intervention* or prevention or policy or policies or program* or project*))
9 #7 OR #8
10 #6 AND #9

Database of Promoting Health Effectiveness Reviews (DoPHER; EPPI-Centre)
1 Freetext (All but Authors): search*
2 Freetext (All but Authors): "systematic" NEAR "review*"
3 Freetext (All but Authors): "systematic" NEAR "overview*"
4 Freetext (All but Authors): "meta analy*"
5 Freetext (All but Authors): metaanaly*
6 Freetext (All but Authors): "umbrella NEAR review"
7 Freetext (All but Authors): "review of reviews"
8 1 OR 2 OR 3 OR 4 OR 5 OR 6 OR 7
9 Freetext (All but Authors): population
10 Freetext (All but Authors): community
11 9 OR 10
12 Freetext (All but Authors): intervention*
13 Freetext (All but Authors): prevention
14 Freetext (All but Authors): policy
15 Freetext (All but Authors): policies
16 Freetext (All but Authors): program*
17 Freetext (All but Authors): project*
18 12 OR 13 OR 14 OR 15 OR 16 OR 17
19 11 AND 18
20 Freetext (All but Authors): health
21 18 AND 20
22 19 OR 21
23 8 AND 22

Social Care Online (SCIE)
1 search* OR systematic review* OR systematic overview OR meta analy* OR metaanaly* OR umbrella review OR "review of reviews"
- ALL FIELDS
2 "population level" OR "population based" OR "population orientated" OR "population oriented" OR "community level" OR "community based" OR "community orientated" OR "community oriented"

- ALL FIELDS

3 intervention* OR prevention OR policy OR policies OR program$ OR project* - **ALL FIELDS**

4 1 AND 2 AND 3

<u>Health Systems Evidence</u>

1 ("population level" OR "population based" OR "population orientated" OR "population oriented" OR "community level" OR "community based" OR "community orientated" OR "community oriented") AND (intervention* OR prevention OR policy OR policies OR program$ OR project*)

Appendix S3: Example extraction record

Bibliographical details	
**Author**	
**Year**	
**Title**	
**Link**	
**Review details**	
**Systematic review?**	
**Domain** (see matrix) **and Interventions**	
**Population**<br>(any age/gender/location etc. restrictions)	
**Health outcome**<br>(morbidity/mortality only)	
**SES measure**<br>(e.g. gap or gradient, individual income, wealth, education, employment or occupational status, benefit receipt; as well as area-level economic indicators)	
**Number of relevant studies in review (total)**	
**Number of databases searched**	
**Time/language/country restrictions**	
**Study design of included studies** (e.g. RCTS, controlled prospective cohort, repeat cross sections)	
**Method of synthesis** (meta-analysis or narrative)	
**Quality** (as measured by systematic review authors)	
**Main findings**	
For relevant studies in the review summaries:	

Study No.	Author(s) of original publication[1]<br><br>Domain/ intervention[2]<br><br>Relevant page numbers	Study design	Setting and participants[3]	Intervention	Inequality[4]	Health outcome	Summary results<br><br>Description and summary results<br>↑ = increase/ improved<br>↓ = decrease/ deteriorated<br>↔ = no change	Quality appraisal[5]	Impact on inequalities[6]

Summary table

Study	No. of relevant studies	Context (setting, country, search timeframe)	Intervention(s)	Summary of results	AMSTAR quality appraisal[7]

Summary paragraph

AMSTAR tool for assessing methodological quality of systematic reviews

	YES/NO/ Can't answer/NA	R-AMSTAR checklist[8]	R-AMSTAR points
**1. Was an 'a priori' design provided?**<br>The research question and inclusion criteria should be established before the conduct of the review.<br>*Note: Need to refer to a protocol, ethics approval, or pre-determined/a priori published research objectives to score a "yes."*		A: 'a priori' design<br>B: statement of inclusion criteria<br>C: PICO/PIPO research question (population, intervention, comparison, prediction, outcome)<br><br>If it satisfies 3 of the criteria →4<br>If it satisfies 2 of the criteria →3<br>If it satisfies 1 of the criteria →2<br>If it satisfies 0 of the criteria →1	
**2. Was there duplicate study selection and data extraction?**<br>There should be at least two independent data extractors and a consensus procedure for disagreements should be in place.<br>*Note: 2 people do study selection, 2 people do data extraction, consensus process or one person checks the other's work.*		A: There should be at least two independent data extractors as stated or implied<br>B: Statement of recognition or awareness of consensus procedure for disagreements<br>C: Disagreements among extractors resolved properly as stated or implied<br><br>If it satisfies 3 of the criteria →4<br>If it satisfies 2 of the criteria →3<br>If it satisfies 1 of the criteria →2<br>If it satisfies 0 of the criteria →1	

[1] If there fewer than five studies that are relevant, these can be included in full here. However if there are more than five, studies should be grouped if interventions are similar.

[2] As detailed in protocol matrix table.

[3] Include only high-income countries and/or additional EU-28 countries (Australia, Austria, Belgium, Canada, Chile, Czech Republic, Denmark, Estonia, Finland, France, Germany, Greece, Hungary, Iceland, Ireland, Italy, Israel, Japan, Korea Republic, Luxembourg, Netherlands, New Zealand, Norway, Poland, Portugal, Slovak Republic, Slovenia, Spain, Sweden, Switzerland, United Kingdom and United States and Bulgaria, Croatia, Cyprus, Latvia, Lithuania, Malta and Romania).

[4] Disadvantage/gradient approach to inequality.

[5] As measured by systematic review.

[6] +, positive intervention effect so it reduces health-related outcomes in low-SES groups or reduces the SES gradient in health-related outcomes; 0, no intervention effect or no effect on SES gradient in health-related outcomes.

[7] Derived from R-AMSTAR.

[8] Please underline which criteria are achieved by the systematic review.

**3. Was a comprehensive literature search performed?**<br>At least two electronic sources should be searched. The report must include years and databases used (e.g., Central, EMBASE, and MEDLINE). Key words and/or MESH terms must be stated and where feasible the search strategy should be provided. All searches should be supplemented by consulting current contents, reviews, textbooks, specialized registers, or experts in the particular field of study, and by reviewing the references in the studies found.<br>*Note: If at least 2 sources + one supplementary strategy used, select "yes" (Cochrane register/Central counts as 2 sources; a grey literature search counts as supplementary).*		A: At least two electronic sources should be searched.<br>B: The report must include years and databases used (e.g. Central, EMBASE, and MEDLINE).<br>C: Key words and/or MESH terms must be stated **AND** where feasible the search strategy outline should be provided such that one can trace the filtering process of the included articles.<br>D: In addition to the electronic databases (PubMed, EMBASE, Medline), all searches should be supplemented by consulting current contents, reviews, textbooks, specialized registers, or experts in the particular field of study, and by reviewing the references in the studies found.<br>E: Journals were "hand-searched" or "manual searched" (i.e. identifying highly relevant journals and conducting a manual, page-by-page search of their entire contents looking for potentially eligible studies)<br><br>If it satisfies 4 or 5 of the criteria → 4<br>If it satisfies 3 of the criteria → 3<br>If it satisfies 2 of the criteria →2<br>If it satisfies 1 or 0 of the criteria → 1	
**4. Was the status of publication (i.e. grey literature) used as an inclusion criterion?**<br>The authors should state that they searched for reports regardless of their publication type. The authors should state whether or not they excluded any reports (from the systematic review), based on their publication status, language etc.<br>*Note: If review indicates that there was a search for "grey literature" or "unpublished literature," indicate "yes." SINGLE database, dissertations, conference proceedings, and trial registries are all considered grey for this purpose. If searching a source that contains both grey and non-grey, must specify that they were searching for grey/unpublished lit.*		A: The authors should state that they searched for reports regardless of their publication type.<br>B: The authors should state whether or not they excluded any reports (from the systematic review), based on their publication status, language etc.<br>C: "Non-English papers were translated" or readers sufficiently trained in foreign language<br>D: No language restriction or recognition of non-English articles<br><br>If it satisfies 3 or 4 of the criteria →4<br>If it satisfies 2 of the criteria →3<br>If it satisfies 1 of the criteria →2<br>If it satisfies 0 of the criteria →1	
**5. Was a list of studies (included and excluded) provided?**<br>A list of included and excluded studies should be provided.<br>*Note: Acceptable if the excluded studies are referenced. If there is an electronic link to the list but the link is dead, select "no."*		A: Table/list/or figure of included studies, a reference list does not suffice.<br>B: Table/list/figure of excluded studies[1] either in the article or in a supplemental source (i.e. online). (Excluded studies refers to those studies seriously considered on the basis of title and/or abstract, but rejected after reading the body of the text)<br>C: Author satisfactorily/sufficiently stated the reason for exclusion of the seriously considered studies.<br>D: Reader is able to retrace the included and the excluded studies anywhere in the article bibliography, reference, or supplemental source<br><br>If it satisfies 4 of the criteria →4<br>If it satisfies 3 of the criteria →3<br>If it satisfies 2 of the criteria →2<br>If it satisfies 1 or 0 of the criteria → 1	
**6. Were the characteristics of the included studies provided?**<br>In an aggregated form such as a table, data from the original studies should be provided on the participants, interventions and outcomes. The ranges of characteristics in all the studies analyzed e.g., age, race, sex, relevant socioeconomic data, disease status, duration, severity, or other diseases should be reported.<br>*Note: Acceptable if not in table format as long as they are described as above.*		A: In an aggregated form such as a table, data from the original studies should be provided on the participants, interventions **AND** outcomes.<br>B: Provide the ranges of **relevant** characteristics in the studies analyzed (e.g. age, race, sex, relevant socioeconomic data, disease status, duration, severity, or other diseases should be reported.)<br>C: The information provided appears to be complete and accurate (i.e. there is a tolerable range of subjectivity here. Is the reader left wondering? If so, state the needed information and the reasoning).<br><br>If it satisfies 3 of the criteria →4<br>If it satisfies 2 of the criteria →3<br>If it satisfies 1 of the criteria →2<br>If it satisfies 0 criteria → 1	
**7. Was the scientific quality of the included studies assessed and documented?**<br>'A priori' methods of assessment should be provided (e.g., for effectiveness studies if the author(s) chose to include only randomized, double-blind, placebo controlled studies, or allocation concealment as inclusion criteria); for other types of studies alternative items will be relevant.<br>*Note: Can include use of a quality scoring tool or checklist, e.g., Jadad scale, risk of bias, sensitivity analysis, etc., or a description of quality items, with some kind of result for EACH study ("low" or "high" is fine, as long as it is clear which studies scored "low" and which scored "high"; a summary score/range for all studies is not acceptable).*		A: 'A priori' methods of assessment should be provided (e.g., for effectiveness studies if the author(s) chose to include only randomized, double-blind, placebo controlled studies, or allocation concealment as inclusion criteria); for other types of studies alternative items will be relevant.<br>B: The scientific quality of the included studies appears to be meaningful.<br>C: Discussion/recognition/awareness of level of evidence<br>D: Quality of evidence should be rated/ranked based on characterized instruments. (Characterized instrument is a created instrument that ranks the level of evidence, e.g. GRADE [Grading of Recommendations Assessment, Development and Evaluation.])	

		If it satisfies 4 of the criteria →4<br>If it satisfies 3 of the criteria →3<br>If it satisfies 2 of the criteria →2<br>If it satisfies 1 or 0 of the criteria →1	
**8. Was the scientific quality of the included studies used appropriately in formulating conclusions?**<br>The results of the methodological rigor and scientific quality should be considered in the analysis and the conclusions of the review, and explicitly stated in formulating recommendations.<br>*Note: Might say something such as "the results should be interpreted with caution due to poor quality of included studies." Cannot score "yes" for this question if scored "no" for question 7.*		A: The results of the methodological rigor and scientific quality should be considered in the analysis and the conclusions of the review<br>B: The results of the methodological rigor and scientific quality are **explicitly stated** in formulating recommendations.<br>C: To have conclusions integrated/drives towards a clinical consensus statement<br>D: This clinical consensus statement drives toward revision or confirmation of clinical practice guidelines<br><br>If it satisfies 4 of the criteria →4<br>If it satisfies 3 of the criteria →3<br>If it satisfies 2 of the criteria →2<br>If it satisfies 1 or 0 of the criteria → 1	
**9. Were the methods used to combine the findings of studies appropriate?**<br>For the pooled results, a test should be done to ensure the studies were combinable, to assess their homogeneity (i.e., Chi-squared test for homogeneity, I2). If heterogeneity exists a random effects model should be used and/or the clinical appropriateness of combining should be taken into consideration (i.e., is it sensible to combine?).<br>*Note: Indicate "yes" if they mention or describe heterogeneity, i.e., if they explain that they cannot pool because of heterogeneity/variability between interventions.*		A: Statement of criteria that were used to decide that the studies analyzed were similar enough to be pooled?<br>B: For the pooled results, a test should be done to ensure the studies were combinable, to assess their homogeneity (i.e. Chi-squared test for homogeneity, I^2).<br>C: Is there a recognition of heterogeneity or lack of thereof<br>D: If heterogeneity exists a "random effects model" should be used and/or the rationale (i.e. clinical appropriateness) of combining should be taken into consideration (i.e. is it sensible to combine?), or stated explicitly<br>E: If homogeneity exists, author should state a rationale or a statistical test<br><br>If it satisfy 4 or 5 of the criteria → 4<br>If it satisfy 3 of the criteria → 3<br>If it satisfy 2 of the criteria →2<br>If it satisfy 1 or 0 of the following criteria → 1	
**10. Was the likelihood of publication bias assessed?**<br>An assessment of publication bias should include a combination of graphical aids (e.g., funnel plot, other available tests) and/or statistical tests (e.g., Egger regression test, Hedges-Olken).<br>*Note: If no test values or funnel plot included, score "no". Score "yes" if mentions that publication bias could not be assessed because there were fewer than 10 included studies.*		A Recognition of publication bias or file-drawer effect<br>B: An assessment of publication bias should include graphical aids (e.g., funnel plot, other available tests)<br>C: Statistical tests (e.g. Egger regression test)<br><br>If it satisfies 3 of the criteria →4<br>If it satisfies 2 of the criteria →3<br>If it satisfies 1 of the criteria →2<br>If it satisfies 0 of the criteria →1	
**11. Was the conflict of interest included?**<br>Potential sources of support should be clearly acknowledged in both the systematic review and the included studies.<br>*Note: To get a "yes," must indicate source of funding or support for the systematic review AND for each of the included studies.*		A: Statement of sources of support<br>B: No conflict of interest. This is subjective and may require some deduction or searching<br>C: An awareness/statement of support or conflict of interest in the **primary** inclusion studies<br><br>If it satisfies 3 of the criteria →4<br>If it satisfies 2 of the criteria →3<br>If it satisfies 1 of the criteria →2<br>If it satisfies 0 of the criteria →1	
	**TOTAL:**	Maximum 44	
		Quality appraisal:<br>Low = 11 -22<br>Medium = 23-33<br>High = 34 - 44	

Data extractor record

Name of data extractor	Date	Comments

Appendix S4: AMSTAR rating for all included studies

#	Authors	Year	1. Was an 'a priori' design provided?	2. Was there duplicate study selection and data extraction?	3. Was a comprehensive literature search performed?	4. Was the status of publication (i.e. grey literature) used as an inclusion criterion?	5. Was a list of studies (included and excluded) provided?	6. Were the characteristics of the included studies provided?	7. Was the scientific quality of the included studies assessed and documented?	8. Was the scientific quality of the included studies used appropriately in formulating conclusions?	9. Were the methods used to combine the findings of studies appropriate?	10. Was the likelihood of publication bias assessed?	11. Was the conflict of interest included?	R-AMSTAR score	R-AMSTAR grade
1	Alagiyawanna et al.	2015	No 2	Yes 4	Yes 4	Yes 3	No 2	Yes 4	Yes 4	Yes 1	Yes 1	No 1	No 3	29	Medium
2	Ashton et al.	2009	No 2	Can't tell 1	Yes 3	Yes 1	Yes 4	Yes 4	No 1	Yes 2	Yes 2	No 1	No 1	22	Low
3	Beauchamp et al.	2014	No 2	Yes 3	Yes 4	Yes 3	No 2	Yes 4	Yes 4	Yes 2	Yes 1	No 2	No 3	30	Medium
4	Benmarhnia et al.	2014	No 2	Yes 2	Yes 3	No 2	No 1	Yes 3	No 1	No 1	Yes 1	No 2	Yes 3	21	Low
5	Black et al.	2012	No 3	Yes 2	Yes 3	Yes 1	Yes 2	Yes 4	Yes 4	Yes 2	Yes 2	Yes 1	No 3	27	Medium
6	Black et al.	2000	No 3	Yes 3	Yes 4	Yes 3	No 1	Yes 4	Yes 4	Yes 3	No 1	No 1	No 1	28	Medium
7	Brown et al.	2014	Yes 4	Yes 2	Yes 4	No 2	No 2	Yes 4	Yes 3	Yes 2	No 1	No 1	No 3	28	Medium
8	Ciliska et al.	2000	No 1	Yes 2	Yes 4	No 2	No 1	Yes 4	Yes 2	Yes 1	No 1	No 1	No 3	22	Low
9	Crocker-Buque et al.	2016	Yes 3	No 1	Yes 4	No 2	No 1	Yes 4	No 1	No 1	Can't tell 1	No 1	Yes 3	22	Low
10	da Sa and Lock	2008	No 3	No 1	Yes 4	Yes 4	No 1	Yes 3	Yes 1	No 1	Yes 1	No 1	No 2	22	Low
11	da Silva et al.	2016	Yes 4	No 1	Yes 4	No 4	Yes 4	Yes 4	Yes 3	Yes 3	Yes 2	Yes 2	No 3	34	High
12	Egan et al.	2007	No 2	Can't tell 2	Yes 4	Yes 4	No 1	Yes 4	Yes 3	Yes 2	Yes 1	No 1	Yes 3	27	Medium
13	Frazer et al.	2016	Yes 3	Yes 4	Yes 4	No 2	Yes 4	Yes 4	Yes 4	Yes 2	No 1	No 1	Yes 3	32	Medium
14	Hendry et al.	2015	Can't tell 3	Yes 4	Yes 4	Yes 4	No 1	Yes 3	Yes 4	Yes 2	Yes 1	No 1	No 3	30	Medium
15	Hillier-Brown et al.	2014	Yes 4	Yes 4	Yes 4	Yes 3	Yes 1	Yes 4	Yes 3	Yes 2	Yes 2	No 1	No 3	31	Medium
16	Hillier-Brown et al.	2017	Yes 4	Yes 4	Yes 3	No 2	Yes 4	Yes 4	Yes 4	Yes 2	Yes 1	No 0	Yes 3	31	Medium
17	Iheozor-Ejiofor et al.	2015	Yes 4	Yes 4	Yes 4	No 3	Yes 4	Yes 4	Yes 4	Yes 2	Yes 2	No 3	No 2	36	High
18	Jackson et al.	2010	Yes 4	No 2	Yes 4	Can't tell 2	No 1	Yes 3	Yes 3	Yes 2	Yes 1	No 1	No 3	26	Medium
19	McGill et al.	2015	Yes 3	Yes 4	Yes 4	Yes 2	No 2	Yes 4	Yes 2	Yes 1	Yes 1	No 1	No 3	27	Medium
20	McLaren et al.	2016	Yes 4	Yes 4	Yes 4	Yes 4	Yes 4	No 2	Yes 4	Yes 2	Yes 4	Yes 3	No 2	37	High
21	Menzies and McIntyre	2006	No 1	No 1	No 2	No 2	No 1	Yes 3	No 1	No 1	No 1	No 1	Yes 3	17	Low
22	Mulvaney et al.	2015	Yes 4	Yes 4	Yes 4	No 3	Yes 4	Yes 3	No 3	Yes 3	No 2	Yes 4	No 3	37	High
23	National Collaborating Cent	2009	No 3	No 1	Yes 4	Yes 3	Yes 4	No 3	Yes 4	No 2	No 1	No 2	No 1	28	Medium
24	Niedderdeppe et al.	2008	Yes 2	Can't tell 1	Yes 3	No 1	No 1	Yes 4	No 1	Yes 1	No 1	No 1	Yes 2	18	Low
25	Olstad et al.	2016	Yes 4	Yes 4	Yes 4	Can't tell 1	No 2	Yes 3	Yes 3	Yes 2	Yes 2	No 1	No 3	29	Medium
26	Spadea et al.	2010	No 1	Yes 3	Yes 4	No 1	No 1	No 1	No 2	No 1	Can't tell 1	No 2	Yes 2	19	Low
27	Stockley and Lund	2008	No 1	No 1	Yes 2	Yes 4	No 1	No 1	No 1	No 1	No 1	No 1	No 3	17	Low
28	Sumar and McLaren	2011	No 1	Yes 4	Yes 3	Yes 2	No 1	No 2	Yes 4	Yes 2	Yes 3	No 2	No 2	26	Medium
29	Thomas et al.	2008	Can't tell 2	Yes 2	Yes 4	Yes 4	No 1	No 3	Can't tell 3	No 1	No 1	No 1	Yes 3	25	Medium

KEY Yes (green) No (red) Can't tell (yellow)

Appendix S5: Excluded articles

Detailed below are full references for all excluded articles. Reasons for exclusion are given at the start of each section. Articles are excluded for one of the following reasons (in order of hierarchical importance as set out below):

1. The paper is not a systematic review (in accordance with the DARE criteria).
2. The paper does not focus on high-income countries.
3. No state-led population health intervention is described.
4. No health outcomes of relevance are described.
5. Inappropriate study design used.
6. No health inequality data is presented.
7. Insufficient detail is given regarding interventions/outcomes/health inequality data.
8. Not a systematic review of primary studies.
9. Unable to locate

The paper is not a systematic review

Anderson P: **Policy implications of the WHO strategy to reduce the harmful use of alcohol**. *Sucht* 2011, **57**(2):85-98.

Anderson P, Chisholm D, Fuhr DC: **Alcohol and global health 2: effectiveness and cost-effectiveness of policies and programmes to reduce the harm caused by alcohol**. *Lancet* 2009, **373**(9682):2234-2246.

Assuncao MCF, Santos IS: **Efeito da fortificação de alimentos com ferro sobre anemia em crianças: um estudo de revisão [Portuguese]. Effect of food fortification with iron on childhood anemia: a review study**. *Cadernos De Saude Publica* 2007, **23**(2):269-281.

Attwood S, van Sluijs E, Sutton S: **Exploring equity in primary-care-based physical activity interventions using PROGRESS-Plus: a systematic review and evidence synthesis**. *International Journal of Behavioral Nutrition and Physical Activity* 2016, **13**:60.

Blake SM, Amaro H, Schwartz PM, Flinchbaugh LJ: **A review of substance abuse prevention interventions for young adolescent girls**. *Journal of Early Adolescence* 2001, **21**(3):294-324.

Blinkhorn AS, Downer MC, Drugan CS: **Policies for improving oral health in Europe**. *Health Education Journal* 2005, **64**(3):197-217.

Botler J, Bastos Camacho LA, da Cruz MM, George P: **Neonatal screening - the challenge of a universal and effective coverage**. *Ciencia & Saude Coletiva* 2010, **15**(2):493-508.

Browne G, Gafni A, Roberts J, Byrne C, Majumdar B: **Effective/efficient mental health programs for school-age children: a synthesis of reviews**. *Social Science & Medicine* 2004, **58**(7):1367-1384.

Browne J, Hayes R, Gleeson D: **Aboriginal health policy: is nutrition the 'gap' in 'Closing the Gap'?** *Australian and New Zealand Journal of Public Health* 2014, **38**(4):362-369.

Brugts JJ, Deckers JW: **Statin prescription in men and women at cardiovascular risk: to whom and when?** *Current Opinion in Cardiology* 2010, **25**(5):484-489.

Cobiac LJ, Veerman L, Vos T: **The role of cost-effectiveness analysis in developing nutrition policy**. *Annual Review of Nutrition* 2013, **33**:373-393.

Cohn E, Kakar S, Farrington D: **Red light camera interventions for reducing traffic violations and accidents: a systematic review**. In: Campbell Collaboration; 2015.

Davoren SL: **Legal interventions to reduce alcohol-related cancers**. *Public Health* 2011, **125**(12):882-888.

Diez E, Peiro R: **Interventions to reduce health inequalities. [Spanish] Intervenciones para disminuir las desigualdades en salud**. *Gaceta Sanitaria* 2004, **18 Suppl 1**:158-167.

Dillner J: **Primary human papillomavirus testing in organized cervical screening**. *Current Opinion in Obstetrics and Gynecology* 2013, **25**(1):11-16.

Duffy SW, Myles JP, Maroni R, Mohammad A: **Rapid review of evaluation of interventions to improve participation in cancer screening services**. *Journal of Medical Screening* 2017, **24**(3):127-145.

Durkin S, Brennan E, Wakefield M: **Mass media campaigns to promote smoking cessation among adults: an integrative review**. *Tobacco Control* 2012, **21**(2):127.

Durlak JA: **Primary prevention programs in schools**. In: *Advances in Clinical Child Psychology. Volume 19*, 1997: 283-318.

Dyreborg J, Lipscomb HJ, Olsen O, Törner M, Nielsen K, Lund J, Kines P, Guldenmund FW, Rasmussen K, Bengtsen E *et al*: **Safety interventions for the prevention of accidents in the work place: a systematic review**. In: Campbell Collaboration; 2015.

Ellsaser G, Trost-Brinkhues G, Albrecht M: **Prävention von Verletzungen bei kleinen Kindern [German]. Injury prevention in young children**. *Bundesgesundheitsblatt, Gesundheitsforschung, Gesundheitsschutz* 2014, **57**(6):681-686.

Foltz JL, May AL, Belay B, Nihiser AJ, Dooyema CA, Blanck HM: **Population-level intervention strategies and examples for obesity prevention in children**. *Annual Review of Nutrition* 2012, **32**:391-415.

Giesbrecht N, Wettlaufer A, Thomas G, Stockwell T, Thompson K, April N, Asbridge M, Cukier S, Mann R, McAllister J *et al*: **Pricing of alcohol in Canada: a comparison of provincial policies and harm-reduction opportunities**. *Drug and Alcohol Review* 2016, **35**(3):289-297.

Glanz K, Hoelscher D: **Increasing fruit and vegetable intake by changing environments, policy and pricing: restaurant-based research, strategies, and recommendations**. *Preventive Medicine* 2004, **39**:S88-S93.

Glanz, K and Yaroch, AL: **Strategies for increasing fruit and vegetable intake in grocery stores and communities: policy, pricing, and environmental change.** *Preventive Medicine* 2004, **39**:S75-S80

Gray JA: **Evidence-based screening in the United Kingdom**. *International Journal of Technology Assessment in Health Care* 2001, **17**(3):400-408.

Gutierrez-Ibarluzea I, Asua J, Latorre K: **Policies of screening for colorectal cancer in European countries**. *International Journal of Technology Assessment in Health Care* 2008, **24**(3):270-276.

Hahn EJ: **Smokefree legislation: a review of health and economic outcomes research**. *American Journal of Preventive Medicine* 2010, **39**(not found):S66-S76.

Hawk ET, Limburg PJ, Viner JL: **Epidemiology and prevention of colorectal cancer**. *Surgical Clinics of North America* 2002, **82**(5):905-941.

Ibrahim JK, Anderson ED, Burris SC, Wagenaar AC: **State laws restricting driver use of mobile communications devices distracted-driving provisions, 1992-2010**. *American Journal of Preventive Medicine* 2011, **40**(6):659-665.

Kearney AJ: **It is time to reconsider policy for population-based mammography screening**. *Journal of Public Health Policy* 2015, **36**(3):259-269.

Kearney M, Waterall J, Lister C: **NHS Health Check: a systematic approach to CVD prevention**. *Primary Care Cardiovascular Journal* 2014, **7**(2):56-59.

Kiszko K, Martinez O, Abrams C, Elbel B: **The influence of calorie labeling on food orders and consumption: a review of the literature**. *Journal of Community Health* 2014, **39**(6):1248-1269.

Kohler S, Minkner P: **Smoke-free laws and direct democracy initiatives on smoking bans in Germany: a systematic review and quantitative assessment**. *International Journal of Environmental Research and Public Health* 2014, **11**(1):685-700.

Lantz PM, Jacobson PD, Warner KE, Wasserman J, Pollack HA, Berson J, Ahlstrom A: **Investing in youth tobacco control: a review of smoking prevention and control strategies**. *Tobacco Control* 2000, **9**(1):47.

Malottki K, Wang D, Andronis L, Barton P, Fry-Smith A, Greenheld W, Pennant M, Moore D: **Providing public health information to prevent skin cancer**. NICE and West Midlands Health Technology Assessment Collaboration, Birmingham, 2009.

Manhart LE, Holmes KK: **Randomized controlled trials of individual-level, population-level, and multilevel interventions for preventing sexually transmitted infections: what has worked?** *Journal of Infectious Diseases* 2005, **191**:S7-24 21.

McArthur DL, Kraus JF: **The specific deterrence of administrative per se laws in reducing drunk driving recidivism**. *American Journal of Preventive Medicine* 1999, **16**(1, Supplement 1):68-75.

McGuffin LE, Wallace JM, McCrorie TA, Price RK, Pourshahidi LK, Livingstone MB: **Family eating out-of-home: a review of nutrition and health policies**. *Proceedings of the Nutrition Society* 2013, **72**(1):126-139.

McKenna ML: **Policy options to support healthy eating in schools**. *Canadian Journal of Public Health* 2010, **101**(SUPPL. 2):S14-S17.

Miech R, Azur M, Dusablon T, Jowers K, Goldstein AB, Stuart EA, Walrath C, Leaf PJ: **The potential to reduce mental health disparities through the comprehensive community mental health services for children and their families program**. *Journal of Behavioral Health Services and Research* 2008, **35**(3):253-264.

Mueller M, Purnell TS, Mensah GA, Cooper LA: **Reducing racial and ethnic disparities in hypertension prevention and control: what will it take to translate research into practice and policy?** *American Journal of Hypertension* 2015, **28**(6):699-716.

Olson KL, Chapman JA, Thurston WE, Milligan CD: **Promotion of breast cancer screening in communities: a research agenda**. *Cancer Prevention & Control* 1997, **1**(3):213-221.

Purcell KR, O'Rourke K, Rivis M: **Tobacco control approaches and inequity--how far have we come and where are we going?** *Health Promotion International* 2015, **30**(Suppl 2):ii89-101.

Quinn LA, Thompson SJ, Ott MK: **Application of the social ecological model in folic acid public health initiatives**. *Journal of Obstetric Gynecologic and Neonatal Nursing* 2005, **34**(6):672-681.

Rivara FP, Thompson DC, Patterson MQ, Thompson RS: **Prevention of bicycle-related injuries: helmets, education, and legislation**. *Annual Review of Public Health* 1998, **19**:293-318.

Rowan PJ, Duckett SA, Wang JE: **State mandates regarding postpartum depression**. *Psychiatric Services* 2015, **66**(3):324-328 325p.

Sargeant JM, Rajic A, Read S, Ohlsson A: **The process of systematic review and its application in agri-food public-health**. *Preventive Veterinary Medicine* 2006, **75**(3-4):141-151.

Sarnoff R, Rundall T: **Meta-analysis of effectiveness of interventions to increase influenza immunization rates among high-risk population groups**. *Medical Care Research and Review* 1998, **55**(4):432-456.

Sassi F, Belloni A: **Fiscal incentives, behavior change and health promotion: what place in the health-in-all-policies toolkit?** *Health Promotion International* 2014, **29**:103-112.

Shemilt I, Hollands GJ, Marteau TM, Nakamura R, Jebb SA, Kelly MP, Suhrcke M, Ogilvie D: **Economic instruments for population diet and physical activity behaviour change: a systematic scoping review**. *Plos One* 2013, **8**(9).

Sin JP, St. Leger AS: **Interventions to increase breast screening uptake: do they make any difference?** *Journal of Medical Screening* 1999, **6**(4):170-181 112.

Snyder LB, Hamilton MA, Mitchell EW, Kiwanuka-Tondo J, Fleming-Milici F, Proctor D: **A meta-analysis of the effect of mediated health communication campaigns on behavior change in the United States**. *Journal of Health Communication* 2004, **9**:71-96.

Stables GJ, Young EM, Howerton MW, Yaroch AL, Kuester S, Solera MK, Cobb K, Nebeling L: **Small school-based effectiveness trials increase vegetable and fruit consumption among youth**. *Journal of the American Dietetic Association* 2005, **105**(2):252-256.

Stice E, Shaw H, Marti CN: **A meta-analytic review of obesity prevention programs for children and adolescents: the skinny on interventions that work**. *Psychological Bulletin* 2006, **132**(5):667-691.

Stone D, Muir R, Logan J, Gorman D: **Injury prevention in Scotland - The case for a national strategy**. *Scottish Medical Journal* 2000, **45**(5):147-149.

Struckman-Johnson DL, Lund AK, Williams AF, Osborne DW: **Comparative effects of driver improvement programs on crashes and violations**. *Accident Analysis & Prevention* 1989, **21**(3):203-215.

Sudarsanam TD, Tharyan P: **Are routine general health checks in healthy adults effective in preventing morbidity and mortality due to cardiovascular diseases and cancer? Summary of the evidence and implications for public healt**. *Clinical Epidemiology and Global Health* 2013, **1**(1):19-22.

Talaulikar VS, Arulkumaran S: **Folic acid in obstetric practice: a review**. *Obstetrical and Gynecological Survey* 2011, **66**(4):240-247.

Tao WJ, Agerholm J, Burstrom B: **The impact of reimbursement systems on equity in access and quality of primary care: a systematic literature review**. *BMC Health Services Research* 2016, **16**:10.

Taylor YJ, Nies MA: **Measuring the impact and outcomes of maternal child health federal programs**. *Maternal and child health journal* 2013, **17**(5):886-896.

Tremblay M-C, Pluye P, Gore G, Granikov V, Filion KB, Eisenberg MJ: **Regulation profiles of e-cigarettes in the United States: a critical review with qualitative synthesis**. *BMC Medicine* 2015, **13**(1):130.

van Lenthe FJ, De Bourdeaudhuij I, Klepp KI, Lien N, Moore L, Faggiano F, Kunst AE, MacKenbach JP: **Preventing socioeconomic inequalities in health behaviour in adolescents in Europe: background, design and methods of project TEENAGE**. *BMC Public Health* 2009, **9**: 125.

Vieira Ulinski Aguilera SL, Moyses ST, Moyses SJ: **Intervenções de segurança viária e seus efeitos nas lesões causadas pelo trânsito: uma revisão sistemática [Portuguese]. Road safety measures and their effects on traffic injuries: a systematic review**. *Revista Panamericana De Salud Publica-Pan American Journal of Public Health* 2014, **36**(4):257-265.

Wakefield M, Chaloupka F: **Effectiveness of comprehensive tobacco control programmes in reducing teenage smoking in the USA**. *Tobacco Control* 2000, **9**(2):177-186.

Weare K, Nind M: **Mental health promotion and problem prevention in schools: what does the evidence say?** *Health Promotion International* 2011, **26**(suppl_1):i29-i69.

White MD: **Pros, cons, and ethics of HPV vaccine in teens-Why such controversy?** *Translational Andrology & Urology* 2014, **3**(4):429-434.

Wodak A, Cooney A: **Effectiveness of sterile needle and syringe programmes**. *International Journal of Drug Policy* 2005, **16**:S31-44 31p.

Woolf, SH: **The accuracy and effectiveness of routine population screening with mammography, prostate-specific antigen, and prenatal ultrasound - a review of published scientific evidence.** *International Journal of Technology Assessment in Health Care 2001,* **17**(3):275-304.

Yu Y, Li G, Liu Q: **Different concentration of iodized salt for preventing iodine deficiency disorder: a systematic review. [Chinese]**. *Chinese Journal of Endemiology* 2009, **28**(5):579-582.

Zhang Q, Liu S, Liu R, Xue H, Wang Y: **Food policy approaches to obesity prevention: an international perspective**. *Current Obesity Reports* 2014, **3**(2):171-182.

<u>The paper does not focus on high-income countries.</u>

Afable A, Karingula NS: **Evidence based review of type 2 diabetes prevention and management in low and middle income countries**. *World Journal of Diabetes* 2016, **7**(10):209-229.

Atun R, Jongh Td, Secci F, Ohiri K, Adeyi O: **A systematic review of the evidence on integration of targeted health interventions into health systems**. *Health policy and planning* 2010, **25**(1):1-14.

Bassani D, Arora PWKGMFLLBZA: **Financial incentives and coverage of child health interventions: a systematic review and meta-analysis**. *BMC Public Health* 2013, **13(Supplement 3)**:S30.

Bright T, Felix L, Kuper H, Polack S: **A systematic review of strategies to increase access to health services among children in low and middle income countries**. *BMC Health Services Research* 2017, **17**: 252.

de Jongh TE, Gurol-Urganci I, Allen E, Zhu NJ, Atun R: **Integration of antenatal care services with health programmes in low- and middle-income countries: systematic review**. *Journal of Global Health* 2016, **6**(1): 010403

Girard AW, Self J, McAuliffe C, Olude O: **The effect of agricultural strategies to improve household food production on the health and nutrition outcomes of women and young children: a systematic review**. *FASEB Journal Conference: Experimental Biology* 2012, **26**(no pagination).

Hunter BM, Harrison S, Portela A, Bick D: **The effects of cash transfers and vouchers on the use and quality of maternity care services: a systematic review**. *Plos One* 2017, **12**(3): : e0173068.

Januario SS, Peixoto FSD, Lima NNR, do Nascimento VB, de Sousa DF, Luz D, da Silva CGL, Neto MLR: **Mental health and public policies implemented in the Northeast of Brazil: a systematic review with meta-analysis**. *International Journal of Social Psychiatry* 2017, **63**(1):21-32.

Joseph J, Basu D: **Efficacy of brief interventions in reducing hazardous or harmful alcohol use in middle-income countries: systematic review of randomized controlled trials**. *Alcohol and Alcoholism* 2017, **52**(1):56-64.

Kalamar AM, Bayer AM, Hindin MJ: **Interventions to prevent sexually transmitted infections, including HIV, among young people in low- and middle-income countries: a systematic review of the published and gray literature**. *Journal of Adolescent Health* 2016, **59**(3):S22-S31.

Manley J, Gitter S, Slavchevska V: **How effective are cash transfers at improving nutritional status?** *World Development* 2013, **48**:133-155.

Moreno R, Nababan HY, Ota E, Wariki WM, Ezoe S, Gilmour S, Shibuya K: **Structural and community-level interventions for increasing condom use to prevent the transmission of HIV and other sexually transmitted infections**. *The Cochrane database of systematic reviews* 2014, **7**:CD003363.

O'Reilly KR, d'Aquila E, Fonner V, Kennedy C, Sweat M: **Can policy interventions affect HIV-related behaviors? A systematic review of the evidence from low- and middle-income countries**. *Aids and Behavior* 2017, **21**(3):626-642.

Pachon H, Spohrer R, Mei Z, Serdula MK: **Evidence of the effectiveness of flour fortification programs on iron status and anemia: a systematic review**. *Nutrition Reviews* 2015, **73**(11):780-795.

Rottach E, Pappa S, Dayal R, Das M: **Deconstructing gender: evidence on how programmes address gender inequalities to improve health**. *Development in Practice* 2017, **27**(2):168-180.

No state-led population health intervention is described.

Abdel-Aleem H, El-Gibaly OMH, El-Gazzar A, Al-Attar GST: **Mobile clinics for women's and children's health**. *Cochrane Database of Systematic Reviews* 2016(8):56.

Adams J, Bateman B, Becker F, Cresswell T, Flynn D, McNaughton R, Oluboyede Y, Robalino S, Ternent L, Sood BG *et al*: **Effectiveness and acceptability of parental financial incentives and quasi-mandatory schemes for increasing uptake of vaccinations in preschool children: systematic review, qualitative study and discrete choice experiment**. *Health Technology Assessment* 2015, **19**(94):1-176.

Adi Y, Schrader McMillan A, Kiloran A, Stewart-Brown S: **Systematic review of the effectiveness of interventions to promote mental wellbeing in primary schools. Report 3: Universal Approaches with focus on prevention of violence and bullying**. The University of Warwick, Warwick.

Adler Alma J, Taylor F, Martin N, Gottlieb S, Taylor Rod S, Ebrahim S: **Reduced dietary salt for the prevention of cardiovascular disease**. *Cochrane Database of Systematic Reviews* 2014(12).

Alouki K, Delisle H, Bermudez-Tamayo C, Johri M: **Lifestyle interventions to prevent type 2 diabetes: a systematic review of economic evaluation studies**. *Journal of Diabetes Research* 2016: article ID 2159890.

Alston LV, Peterson KL, Jacobs JP, Allender S, Nichols M: **A systematic review of published interventions for primary and secondary prevention of ischaemic heart disease (IHD) in rural populations of Australia**. *BMC Public Health* 2016, **16**(1): 895.

Alvarez-Bueno C, Cavero-Redondo I, Martinez-Andres M, Arias-Palencia N, Ramos-Blanes R, Salcedo-Aguilar F: **Effectiveness of multifactorial interventions in primary health care settings for primary prevention of cardiovascular disease: a systematic review of systematic reviews**. *Preventive Medicine* 2015, **76**(S):S68-S75.

Ammari JB, Baqain ZH, Ashley PF: **Effects of programs for prevention of early childhood caries. A systematic review**. *Medical Principles and Practice* 2007, **16**(6):437-442.

An, R: **Effectiveness of subsidies in promoting healthy food purchases and consumption: a review of field experiments**. *Public Health Nutrition* 2013, **16**(7):1215-1228.

Anderson L, Adeney K, Shinn C, Safranek S, Buckner-Brown J, Krause LK: **Community coalition-driven interventions to reduce health disparities among racial and ethnic minority populations**. *Cochrane Database of Systematic Reviews* 2015(6).

Anderson LM, Scrimshaw SC, Fullilove MT, Fielding JE, Normand J, Task Force Community Preventive S: **Culturally competent healthcare systems - a systematic review**. *American Journal of Preventive Medicine* 2003, **24**(3):68-79.

Anderson LM, Shinn C, Fullilove MT, Scrimshaw SC, Fielding JE, Normand J, Carande-Kulis VG, Task Force Community Preventive S: **The effectiveness of early childhood development programs - a systematic review**. *American Journal of Preventive Medicine* 2003, **24**(3):32-46.

Antonio MC, Chung-Do JJ: **Systematic review of interventions focusing on Indigenous adolescent mental health and substance use**. *American Indian & Alaska Native Mental Health Research* 2015, **22**(3):36-56.

Araujo M, Moraga C, Chapman E, Barreto J, Illanes E: **Intervenciones para mejorar el acceso a los servicios de salud de los pueblos indígenas en las Américas [In Portuguese]. Interventions to improve access to health services by indigenous peoples in the Americas**. *Revista Panamericana De Salud Publica* 2016, **40**(5):371-381.

Arbesman M, Bazyk S, Nochajski SM: **Systematic review of occupational therapy and mental health promotion, prevention, and intervention for children and youth**. *The American Journal of Occupational Therapy* 2013, **67**(6):e120-e130.

Arblaster L, Lambert M, Entwistle V, Forster M: **A systematic review of the effectiveness of health service interventions aimed at reducing inequalities in health**. *Journal of Health Services Research and Policy* 1996, **1**(2):93-103.

Arnott B, Rehackova L, Errington L, Sniehotta FF, Roberts J, Araujo-Soares V: **Efficacy of behavioural interventions for transport behaviour change: systematic review, meta-analysis and intervention coding**. *International Journal of Behavioral Nutrition & Physical Activity* 2014, **11**:133.

Attree P: **Low-income mothers, nutrition and health: a systematic review of qualitative evidence**. *Maternal and Child Nutrition* 2005, **1**(4):227-240.

Atun R, de Jongh TE, Secci FV, Ohiri K, Adeyi O, Car J: **Integration of priority population, health and nutrition interventions into health systems: systematic review**. *BMC Public Health* 2011, **11**:780.

Austin LT, Ahmad F, McNally MJ, Stewart DE: **Breast and cervical cancer screening in Hispanic women: a literature review using the health belief model**. *Women's Health Issues* 2002, **12**(3):122-128.

Bader P, Boisclair D, Ferrence R: **Effects of tobacco taxation and pricing on smoking behavior in high risk populations: a knowledge synthesis**. *International Journal of Environmental Research and Public Health* 2011, **8**(11):4118-4139.

Bailey TM, Delva J, Gretebeck K, Siefert K, Ismail A: **A systematic review of mammography educational interventions for low- income women**. *American Journal of Health Promotion* 2005, **20**(2):96-107.

Bala M, Strzeszynski L, Topor-Madry R, Cahill K: **Mass media interventions for smoking cessation in adults**. *Cochrane Database of Systematic Reviews* 2013(6).

Balogun OO, O'Sullivan EJ, McFadden A, Ota E, Gavine A, Garner CD, Renfrew MJ, MacGillivray S: **Interventions for promoting the initiation of breastfeeding**. *Cochrane Database of Systematic Reviews* 2016(11).

Baxi R, Sharma M, Roseby R, Polnay A, Priest N, Waters E, Spencer N, Webster P: **Family and carer smoking control programmes for reducing children's exposure to environmental tobacco smoke**. *Cochrane Database of Systematic Reviews* 2014(3).

Beach, MC, Gary, TL, Price, EG, Robinson, K, Gozu, A, Palacio, A, Smarth, C, Jenckes, M, Feuerstein, C, Bass, EB, Powe, NR, Cooper, LA: **Improving health care quality for racial/ethnic minorities: a systematic review of the best evidence regarding provider and organization interventions**. *BMC Public Health* 2006, **6,** 104.

Beelmann A, Pfost M, Schmitt C: **Prävention und gesundheitsförderung bei kindern und jugendlichen: Eine meta-analyse der deutschsprachigen wirksamkeitsforschung. = Prevention and health promotion in children and adolescents: A meta-analysis**. *Zeitschrift für Gesundheitspsychologie* 2014, **22**(1):1-14.

Bellamy, R: **A systematic review of educational interventions for promoting sun protection knowledge, attitudes and behaviour following the QUESTS approach.** *Medical Teacher* 2005, **27**(3), 269-275.

Bennett GG, Steinberg DM, Stoute C, Lanpher M, Lane I, Askew S, Foley PB, Baskin ML: **Electronic health (eHealth) interventions for weight management among racial/ethnic minority adults: a systematic review**. *Obesity Reviews* 2014, **15**:146-158.

Bhui KS, Dinos S, Stansfeld SA, White PD: **A synthesis of the evidence for managing stress at work: A review of the reviews reporting on anxiety, depression, and absenteeism**. *Journal of Environmental and Public Health* 2012:515874

Black ME, Yamada J, Mann V: **A systematic literature review of the effectiveness of community-based strategies to increase cervical cancer screening**. *Canadian Journal of Public Health* 2002, **93**(5):386-393.

Blank L, Baxter SK, Payne N, Guillaume LR, Squires H: **Systematic review and narrative synthesis of the effectiveness of contraceptive service interventions for young people, delivered in health care settings**. *Health Education Research* 2012, **27**(6):1102-1119.

Boelsen-Robinson T, Peeters A, Beauchamp A, Chung A, Gearon E, Backholer K: **A systematic review of the effectiveness of whole-of-community interventions by socioeconomic position**. *Obesity Reviews* 2015, **16**(9):806-816.

Bonell C, Dickson K, Hinds K, Melendez-Torres G, Stansfield C, Fletcher A, Thomas J, Lester K, Oliver E, Murphy S *et al*: **The effects of Positive Youth Development interventions on substance use, violence and inequalities: systematic review of theories of change, processes and outcomes. Southampton (UK)**. *Public Health Research* 2016, **No. 4.5.**

Bonell C, Parry W, Wells H, Jamal F, Fletcher A, Harden A, Thomas J, Campbell R, Petticrew M, Murphy S *et al*: **The effects of the school environment on student health: a systematic review of multi-level studies**. *Health and Place* 2013, **21**:180-191.

Bonfill X, Marzo M, Pladevall M, Marti J, Emparanza JI: **Strategies for increasing women participation in community breast cancer screening**. *Cochrane Database of Systematic Reviews* 2001(1):CD002943.

Borsari B, Hustad JTP, Capone C: **Alcohol use in the Greek system, 1999–2009: a decade of progress**. *Current drug abuse reviews* 2009, **2**(3):216-255.

Bouzid M, Hooper L, Hunter PR: **The effectiveness of public health interventions to reduce the health impact of climate change: a systematic review of systematic reviews**. *PloS one* 2013, **8**(4):e62041.

Bowie JV, Curbow BA, Garza MA, Dreyling EK, Benz Scott LA, McDonnell KA: **A review of breast, cervical, and colorectal cancer screening interventions in older women**. *Cancer Control* 2005, **12 Suppl 2**:58-69.

Branscum P, Sharma M: **A systematic analysis of childhood obesity prevention interventions targeting Hispanic children: Lessons learned from the previous decade**. *Obesity Reviews* 2011, **12**(501):e151-e158.

Brinn MP, Carson KV, Esterman AJ, Chang AB, Smith BJ: **Mass media interventions for preventing smoking in young people**. *Cochrane Database of Systematic Reviews* 2010(11).

Broeders M, Moss S, Nystrom L, Njor S, Jonsson H, Paap E, Massat N, Duffy S, Lynge E, Paci, E: **The impact of mammographic screening on breast cancer mortality in Europe: a review of observational studies**. Journal of Medical Screening: 2012, **19**, S1, 14-21.

Brown T, Summerbell C: **Systematic review of school-based interventions that focus on changing dietary intake and physical activity levels to prevent childhood obesity: an update to the obesity guidance produced by the Natio**. *Obesity reviews* 2009, **10**(1):110-141.

Bryant J, Bonevski B, Paul C, McElduff P , Attia J. **A systematic review and meta-analysis of the effectiveness of behavioural smoking cessation interventions in selected disadvantaged groups**. *Addition*: 2011, **106**(9), 1568-1585.

Burns DK, Jones AP, Suhrcke M: **The relationship between international trade and non-nutritional health outcomes: A systematic review of quantitative studies**. *Social Science & Medicine* 2016, **152**:9-17.

Cagetti MG, Campus G, Milia E, Lingstrom P: **A systematic review on fluoridated food in caries prevention**. *Acta odontologica Scandinavica* 2013, **71**(3-4):381-387.

Cahill K, Lancaster T: **Workplace interventions for smoking cessation**. *Cochrane Database of Systematic Reviews* 2014(2).

Cairns G, Angus K, Hastings G, Caraher M: **Systematic reviews of the evidence on the nature, extent and effects of food marketing to children. A retrospective summary**. *Appetite* 2013, **62**:209-215.

Cairns J-M, Bambra C, Hillier-Brown FC, Moore HJ, Summerbell CD: **Weighing up the evidence: a systematic review of the effectiveness of workplace interventions to tackle socio-economic inequalities in obesity**. *Journal of Public Health* 2015, **37**(4):659-670.

Caldwell MA, Miaskowski C: **Mass media interventions to reduce help-seeking delay in people with symptoms of acute myocardial infarction: time for a new approach?** *Patient Education and Counseling* 2002, **46**(1):1-9.

Campbell K, Waters E, O'Meara S, Summerbell C: **Interventions for preventing obesity in childhood. A systematic review**. *Obes Rev* 2001, **2**(3):149-157.

Campbell KJ, Hesketh KD: **Strategies which aim to positively impact on weight, physical activity, diet and sedentary behaviours in children from zero to five years. A systematic review of the literature**. *Obesity Reviews* 2007, **8**(4):327-338.

Campos S, Doxey J, Hammond D: **Nutrition labels on pre-packaged foods: a systematic review**. *Public Health Nutrition* 2011, **14**(8):1496-1506.

Canadian Task Force on the Periodic Health Examination: **Periodic health examination, 1994 update: 3. Primary and secondary prevention of neural tube defects**. *Canadian Medical Association Journal* 1994, **151**(2):159-166.

Carlin A, Murphy MH, Gallagher AM: **Do interventions to increase walking work? A systematic review of interventions in children and adolescents**. *Sports Medicine* 2016, **46**(4):515-530.

Carnevale TD: **Universal adolescent depression prevention programs: a review**. *Journal of School Nursing* 2013, **29**(3):181-195.

Carson KV, Brinn MP, Labiszewski NA, Peters M, Chang AB, Veale A, Esterman AJ, Smith BJ: **Interventions for tobacco use prevention in Indigenous youth**. *Cochrane Database of Systematic Reviews* 2012(8).

Cavin EL: **Culturally safe oral health care for Aboriginal peoples of Canada**. *Canadian Journal of Dental Hygiene* 2015, **49**(1):21-28 28.

Chapman J, Qureshi N, Kai J: **Effectiveness of physical activity and dietary interventions in South Asian populations: a systematic review**. *British Journal of General Practice* 2013, **63**(607):e104-e114.

Charania, MR, Crepaz N, Guenther-Gray, C, Henny, K, Liau, A, Willis, LA, Lyles, CM: **Efficacy of structural-level condom distribution interventions: a meta-analysis of U.S. and international studies, 1998-2007**. *AIDS and Behaviour* 2011, **15**(7), 1283-97.

Chaudhary N, Kreiger N: **Nutrition and physical activity interventions for low-income populations**. *Canadian Journal of Dietetic Practice & Research* 2007, **68**(4):201-206.

Cheong AT, Liew SM, Khoo EM, Zaidi NFM, Chinna K: **Are interventions to increase the uptake of screening for cardiovascular disease risk factors effective? A systematic review and meta-analysis**. *BMC Family Practice* 2017, **18**(1): 4.

Chi DL: **Reducing Alaska Native paediatric oral health disparities: a systematic review of oral health interventions and a case study on multilevel strategies to reduce sugar-sweetened beverage intake**. *International Journal of Circumpolar Health* 2013, **72**:21066.

Chilton R, Pearson M, Anderson R: **Health promotion in schools: a scoping review of systematic reviews**. *Health Education* 2015, **115**(3-4):357-376.

Chircop A, Bassett R, Taylor E: **Evidence on how to practice intersectoral collaboration for health equity: a scoping review**. *Critical Public Health* 2015, **25**(2):178-191 114p.

Chu AHY, Ng SHX, Tan CS, Win AM, Koh D, Muller-Riemenschneider F: **A systematic review and meta-analysis of workplace intervention strategies to reduce sedentary time in white-collar workers**. *Obesity Reviews* 2016, **17**(5):467-481.

Clelland N, Gould T, Parker E: **Searching for evidence: what works in Indigenous mental health promotion?** *Health Promotion Journal of Australia* 2007, **18**(3):208-216.

Clifford A, Pulver LJ, Richmond R, Shakeshaft A, Ivers R: **Disseminating best-evidence health-care to Indigenous health-care settings and programs in Australia: identifying the gaps**. *Health Promotion International* 2009, **24**(4):404-415.

Clifford A, Pulver LJ, Richmond R, Shakeshaft A, Ivers R: **Smoking, nutrition, alcohol and physical activity interventions targeting Indigenous Australians: rigorous evaluations and new directions needed**. *Australian and New Zealand Journal of Public Health* 2011, **35**(1):38-46.

Colditz GA, Brewer TF, Berkey CS, Wilson ME, Burdick E, Fineberg HV, Mosteller F: **Efficacy of BCG vaccine in the prevention of tuberculosis. Meta-analysis of the published literature**. *JAMA* 1994, **271**(9):698-702.

Colquitt Jill L, Loveman E, O'Malley C, Azevedo Liane B, Mead E, Al-Khudairy L, Ells Louisa J, Metzendorf M-I, Rees K: **Diet, physical activity, and behavioural interventions for the treatment of overweight or obesity in preschool children up to the age of 6 years**. In: *Cochrane Database of Systematic Reviews.* 2016.

Corcoran J, Dattalo PCM: **Cervical cancer screening interventions for US Latinas: a systematic review**. *Health and Social Work* 2012, **37**(4):197-205.

Corrieri S, Heider D, Conrad I, Blume A, Koenig H-H, Riedel-Heller SG: **School-based prevention programs for depression and anxiety in adolescence: a systematic review**. *Health Promotion International* 2014, **29**(3):427-441.

Courtney RJ, Paul CL, Sanson-Fisher RW, Carey ML, Macrae FA, Yoong SL: **Community approaches to increasing colorectal screening uptake: a review of the methodological quality and strength of current evidence**. *Cancer Forum* 2012, **36**(1).

Cox LS, Okuyemi K, Choi WS, Ahluwalia JS: **A review of tobacco use treatments in US ethnic minority populations**. *American Journal of Health Promotion* 2011, **25**(5):S11-S30.

Crawford J, Ahmad F, Beaton D, Bierman AS: **Cancer screening behaviours among South Asian immigrants in the UK, US and Canada: a scoping study**. *Health and Social Care in the Community* 2016, **24**(2):123-153.

Crawford-Williams F, Fielder A, Mikocka-Walus A, Esterman A: **A critical review of public health interventions aimed at reducing alcohol consumption and/or increasing knowledge among pregnant women**. *Drug and Alcohol Review* 2015, **34**(2):154-161.

Crepaz N, Marshall KJ, Aupont LW, Jacobs ED, Mizuno Y, Kay LS, Jones P, McCree DH, O'Leary A: **The efficacy of HIV/STI behavioral interventions for African American females in the United States: a meta-analysis**. *American Journal of Public Health* 2009, **99**(11):2069-2078 2010p.

da Silva LS, Cotta RMM, Rosa CdOB: **Estratégias de promoção da saúde e prevenção primária para enfrentamento das doenças crônicas: revisão sistemática [Portuguese]. Health promotion and primary prevention strategies to fight chronic disease: a systematic review**. *Revista Panamericana de Salud Publica* 2013, **34**(5):343-350 348.

Daniel-Ulloa J, Ulibarri M, Baquero B, Sleeth C, Harig H, Rhodes SD: **Behavioral HIV prevention interventions among Latinas in the US: a systematic review of the evidence**. *Journal of Immigrant and Minority Health* 2016, **18**(6):1498-1521.

Davis AM, Vinci LM, Okwuosa TM, Chase AR, Huang ES: **Cardiovascular health disparities - a systematic review of health care interventions**. *Medical Care Research and Review* 2007, **64**(5):S29S-100.

de Jong T, Wiezer N, de Weerd M, Nielsen K, Mattila-Holappa P, Mockallo Z: **The impact of restructuring on employee well-being: a systematic review of longitudinal studies**. *Work and Stress* 2016, **30**(1):91-114.

DiGiacomo M, Davidson PM, Abbott PA, Davison J, Moore L, Thompson SC: **Smoking cessation in indigenous populations of Australia, New Zealand, Canada, and the United States: elements of effective interventions**. *International Journal of Environmental Research and Public Health* 2011, **8**(2):388-410.

Ditter SM, Elder RW, Shults RA, Sleet DA, Compton R, Nichols JL: **Effectiveness of designated driver programs for reducing alcohol-impaired driving: a systematic review**. *American Journal of Preventive Medicine* 2005, **28**(5,Suppl):280-287.

Dobbins M, DeCorby K, Manske S, Goldblatt E: **Effective practices for school-based tobacco use prevention**. *Preventive Medicine* 2008, **46**(4):289-297.

Dowswell T, Towner E: **Social deprivation and the prevention of unintentional injury in childhood: a systematic review**. *Health Education Research* 2002, **17**(2), 221-237.

Durand MA, Carpenter L, Dolan H, Bravo P, Mann M, Bunn F, Elwyn G: **Do interventions designed to support shared decision-making reduce health inequalities? A systematic review and meta-analysis**. *PloS one* 2014, **9**(4):e94670.

Durlak JA, Wells AM: **Primary prevention mental health programs for children and adolescents: a meta-analytic review**. *American Journal of Community Psychology* 1997, **25**(2):115-152.

Ebrahim S, Davey Smith G, McCabe C, Payne N, Pickin M, Sheldon TA, Lampe F, Sampson F, Ward S, Wannamethee G: **What role for statins? A review and economic model**. *Health Technology Assessment* 1999, **3**(19):iii-82.

Eddy JM, Fitzhugh EC, Wojtowicz GG, Wang MQ: **The impact of worksite-based safety belt programs: a review of the literature**. American Journal of Health Promotion, **11**(4), 281-289.

Kanner EFS, Dickinson HO, Beyer FR, Campbell F, Schlesinger C, Healther N, Saunders J, Burnand B, Pienaar ED: **Effectiveness of brief alcohol interventions in primary care populations**. *Cochrane Database of Systematic Reviews* 2007.

Ehiri JE, Ejere HOD, Magnussen L, Emusu D, King W, Osberg JS. **Interventions for promoting booster seat use in four to eight year olds traveling in motor vehicles**. *Cochrane Database of Systematic Reviews* 2006.

Einarson TR, Vicente C, Machado M, Covert D, Trope GE, Iskedjian M: **Screening for glaucoma in Canada: A systematic review of the literature**. *Canadian Journal of Ophthalmology* 2006, **41**(6):709-721.

Elder RW, Nichols JL, Shults RA, Sleet DA, Barrios LC, Compton R: **Effectiveness of school-based programs for reducing drinking and driving and riding with drinking drivers - a systematic review**. *American Journal of Preventive Medicine* 2005, **28**(5):288-304.

el-Guebaly N, Cathcart J, Currie S, Brown D, Gloster S: **Public health and therapeutic aspects of smoking bans in mental health and addiction settings**. *Psychiatric Services* 2002, **53**(12):1617-1622.

Engbers LH, van Poppel MNM, Paw M, van Mechelen W: **Worksite health promotion programs with environmental changes - A systematic review**. *American Journal of Preventive Medicine* 2005, **29**(1):61-70.

Eriksen MP, Gottlieb NH: **A review of the health impact of smoking control at the workplace**. *American Journal of Health Promotion* 1998, **13**(2):83-104.

Escaron AL, Meinen AM, Nitzke SA, Martinez-Donate AP: **Supermarket and grocery store-based interventions to promote healthful food choices and eating practices: a systematic review**. *Prev Chronic Dis* 2013, **10**:E50.

European Centre for Disease P, Control: **Evidence for the effectiveness of interventions to prevent infections among people who inject drugs. Part 1: needle and syringe programmes and**. 2011.

Everett T, Bryant A, Griffin MF, Martin-Hirsch PPL, Forbes CA, Jepson RG: **Interventions targeted at women to encourage the uptake of cervical screening**. *Cochrane Database of Systematic Reviews* 2011(5).

Farahmand FK, Grant KE, Polo AJ, Duffy SN: **School-based mental health and behavioral programs for low-income, urban youth: a systematic and meta-analytic review**. *Clinical Psychology: Science and Practice* 2011, **18**(4):372-390.

Farahmand FK, Duffy SN, Tailor MA, DuBois DL, Lyon AL, K.E. G, Zarlinski JC, Masini O, K.J. Z, Nathanson AM: **Community-based mental health and behavioral programs for low-income urban youth: a meta-analytic review**. *Clinical Psychology: Science and Practice* 2012, **19**(2):195-215.

Fayter D, Nixon J, Hartley S, Rithalia A, Butler G, Rudolf M, Glasziou P, Bland M, Stirk L, Westwood M: **A systematic review of the routine monitoring of growth in children of primary school age to identify growth-related conditions**. *Health Technology Assessment* 2007, **11**(40):1-182 182p.

Ferrer HB, Trotter C, Hickman M, Audrey S: **Barriers and facilitators to HPV vaccination of young women in high-income countries: a qualitative systematic review and evidence synthesis**. *BMC Public Health* 2014, **14**:700.

Ferroni E, Camilloni L, Jimenez B, Furnari G, Borgia P, Guasticchi G, Giorgi Rossi P: **How to increase uptake in oncologic screening: a systematic review of studies comparing population-based screening programs and spontaneous access**. *Preventive Medicine* 2012, **55**(6):587-596 510.

Flament MF, Nguyen H, Furino C, et al.: **Evidence-based primary prevention programmes for the promotion of mental health in children and adolescents: a systematic worldwide review**. *The Mental Health of Children and Adolescents: An Area of Global Neglect* 2007:65-136.

Fleischhacker S, Roberts E, Camplain R, Evenson KR, Gittelsohn J: **Promoting physical activity among Native American youth: a systematic review of the methodology and current evidence of physical activity interventions and community-wide initiatives**. *Journal of Racial and Ethnic Health Disparities* 2016, **3**(4):608-624.

Flodmark CE, Marcus C, Britton M: **Interventions to prevent obesity in children and adolescents: a systematic literature review**. *International Journal of Obesity* 2006, **30**(4):579-589.

Forsetlund L, Eike MC, Vist GE: **Effect of interventions to improve health care services for ethnic minority populations**. *Norsk Epidemiologi* 2010, **20**(1):41-52.

Foxcroft DR, Tsertsvadze A: **Universal family-based prevention programs for alcohol misuse in young people**. *Cochrane Database of Systematic Reviews* 2011(9).

Foxcroft DR, Tsertsvadze A: **Universal multi-component prevention programs for alcohol misuse in young people**. *Cochrane Database of Systematic Reviews* 2011(9).

Fraser SD, Lock K: **Cycling for transport and public health: a systematic review of the effect of the environment on cycling**. *European Journal of Public Health* 2011, **21**(6):738-743.

French RS, Bonell C, Wellings K, Weatherburn P: **An exploratory review of HIV prevention mass media campaigns targeting men who have sex with men**. *BMC Public Health* 2014, **14**: 616.

French SA, Stables G: **Environmental interventions to promote vegetable and fruit consumption among youth in school settings**. *Preventive Medicine* 2003, **37**(6):593-610.

Gardois P, Booth A, Goyder E, Ryan T: **Health promotion interventions for increasing stroke awareness in ethnic minorities: a systematic review of the literature**. *BMC Public Health* 2014, **14**:409.

Gelormino E, Melis G, Marietta C, Costa G: **From built environment to health inequalities: an explanatory framework based on evidence**. *Preventive Medicine Reports* 2015, **2**:737-745.

Ginige S, Fairley CK, Hocking JS, Bowden FJ, Chen MY: **Interventions for increasing chlamydia screening in primary care: a review**. *BMC Public Health* 2007, **7**(147):95.

Giorgi Rossi P, Camilloni L, Cogo C, Federici A, Ferroni E, Furnari G, Giordano L, Grazzini G, Iossa A, Jimenez B *et al*: **[Methods to increase participation in cancer screening programmes]**. *Epidemiologia e Prevenzione* 2012, **36**(1 Suppl 1):1-104.

Glanz K, Sorensen G, Farmer A: **The health impact of worksite nutrition and cholesterol intervention programs**. *American Journal of Health Promotion* 1996, **10**(6):453-470.

Glick SB, Clarke AR, Blanchard A, Whitaker AK: **Cervical cancer screening, diagnosis and treatment interventions for racial and ethnic minorities: a systematic review**. *Journal of General Internal Medicine* 2012, **27**(8):1016-1032.

Gonzalez S, TH Z, Wang J, Noor A, Springer D: **Interventions promoting colorectal cancer screening in the Hispanic population: a review of the literature**. *Journal of Nursing Scholarship* 2012, **44**(4):332-40.

Gordon R, McDermott L, Stead M, Angus K: **The effectiveness of social marketing interventions for health improvement: what's the evidence?** *Public Health* 2006, **120**(12):1133-1139.

Gorin S, Westhoff C: **Ethnic/racial barriers to cervical cancer prevention with the HPV vaccine**. *Journal of Clinical Oncology* 2009, **1**:1546.

Gorin SS, Badr H, Krebs P, Das IP: **Multilevel interventions and racial/ethnic health disparities**. *Journal of the National Cancer Institute - Monographs* 2012, **2012**(44):100-111.

Green J, Howes F, Waters E, Maher E, Oberklaid F: **Promoting the Social and Emotional Health of Primary School-Aged Children: Reviewing the Evidence Base for School-Based Interventions**. *International Journal of Mental Health Promotion* 2005, **7**(3):30-36.

Griner D, Smith TB: **Culturally adapted mental health intervention: a meta-analytic review. Special issue: Culture, race, and ethnicity in psychotherapy**. *Psychotherapy: Theory, Research, Practice, Training* 2006, **43**(4):531-548.

Grossman DC, Garcia CC: **Effectiveness of health promotion programs to increase motor vehicle occupant restraint use among young children**. *American Journal of Preventive Medicine* 1999, **16**(1S1):12-22.

Gudzune K, Hutfless S, Maruthur N, Wilson R, Segal J: **Strategies to prevent weight gain in workplace and college settings: a systematic review**. *Preventive Medicine* 2013, **57**(4):268-277.

Guy RJ, Ali H, Liu B, Poznanski S, Ward J, Donovan B, Kaldor J, Hocking J: **Efficacy of interventions to increase the uptake of chlamydia screening in primary care: a systematic review**. *BMC Infectious Diseases* 2011, **11:** 211.

Guyer B, Ma S, Grason H, Frick KD, Perry DF, Sharkey A, McIntosh J: **Early childhood health promotion and its life course health consequences**. *Academic Pediatrics* 2009, **9**(3):142-149.e171.

Hagenzieker MP, Bijleveld FD, Davidse RJ: **Effects of incentive programs to stimulate safety belt use: a meta-analysis**. *Accident Analysis & Prevention* 1997, **29**(6):759-777.

Hale DR, Fitzgerald-Yau N, Mark Viner R: **A systematic review of effective interventions for reducing multiple health risk behaviors in adolescence**. *American Journal of Public Health* 2014, **104**(5):e19-41 11p.

Hamberg-van Reenen HH, Proper KI, van den Berg M: **Worksite mental health interventions: a systematic review of economic evaluations**. *Occupational & Environmental Medicine* 2012, **69**(11):837-845.

Hamilton F, Greaves F, Majeed A, Millett C: **Effectiveness of providing financial incentives to healthcare professionals for smoking cessation activities: systematic review**. *Tobacco Control* 2013, **22**(1):3-8.

Hamm MP, Shulhan J, Williams G, Milne A, Scott SD, Hartling L: **A systematic review of the use and effectiveness of social media in child health**. *BMC Pediatrics* 2014, **14**:138.

Han HR, Kim JY, Lee JE, Hedlin HK, Song HJ, Song YS, Kim MT: **Interventions that increase use of Pap tests among ethnic minority women: a meta-analysis**. *Psycho-Oncology* 2011, **20**(4):341-351.

Hawksley B, Carnwell R, Callwood I: **A literature review of the public health roles of health visitors and school nurses**. *British Journal of Community Nursing* 2003, **8**(10):447-454.

Healey C, Rahman A, Faizal M, Kinderman P: **Underage drinking in the UK: Changing trends, impact and interventions. A rapid evidence synthesis**. *International Journal of Drug Policy* 2014, **25**(1):124-132 129.

Hebden L, Chey T, Allman-Farinelli M: **Lifestyle intervention for preventing weight gain in young adults: a systematic review and meta-analysis of RCTs**. *Obesity Reviews* 2012, **13**(8):692-710.

Henderson S, Kendall E, See L: **The effectiveness of culturally appropriate interventions to manage or prevent chronic disease in culturally and linguistically diverse communities: a systematic literature review**. *Health & Social Care in the Community* 2011, **19**(3):225-249 225p.

Henschel S, Atkinson R, Zeka A, Le Tertre A, Analitis A, Katsouyanni K, Chanel O, Pascal M, Forsberg B, Medina S *et al*: **Air pollution interventions and their impact on public health**. *International Journal of Public Health* 2012, **57**(5):757-768.

Heo HH, Braun K: **Culturally tailored interventions of chronic disease targeting Korean Americans: a systematic review**. *Ethnicity & Health* 2014, **19**:64-85.

Hewitson P, Glasziou P, Irwig L, Towler B, Watson E: **Screening for colorectal cancer using the faecal occult blood test, Hemoccult**. *Cochrane Database of Systematic Reviews* 2007, **1:** CD001216.

Hieke S, Taylor CR: **A critical review of the literature on nutritional labelling**. *Journal of Consumer Affairs* 2012, **46**(1):120-156.

Hijar G, Aramburu A, Hurtado Y, Suárez V: **Fortificación del arroz para corregir la deficiencia de micronutrientes en niños de 6 a 59 meses de edad [In Spanish]. Rice fortification to correct micronutrient deficiency in children 6–59 months old**. *Revista Panamericana de Salud Publica* 2015, **37**(1):52-58.

Hillier-Brown FC, Bambra CL, Cairns JM, Kasim A, Moore HJ, Summerbell CD: **A systematic review of the effectiveness of individual, community and societal level interventions at reducing socioeconomic inequalities in obesity amongst children**. *BMC Public Health* 2014, **14**:834.

Hoffman-Goetz L, Friedman DB: **A systematic review of culturally sensitive cancer prevention resources for ethnic minorities**. *Ethnicity and Disease* 2006: 16(4), 971-7.

Holden DJ, Jonas DE, Porterfield DS, Reuland D, Harris R: **Systematic review: Enhancing the use and quality of colorectal cancer screening**. *Annals of Internal Medicine* 2010, **152**(10):668-76.

Hooper L, Bartlett C, Davey Smith, G, Ebrahim S: **Systematic review of long term effects of advice to reduce dietary salt in adults**. 2002*:* **325**: 628.

Hosking J, Macmillan A, Connor J, Bullen C, Ameratunga S: **Organisational travel plans for improving health**. *Cochrane Database of Systematic Reviews* **2010** (3).

Hou SI, Sealy DA, Kabiru CW: **Closing the disparity gap: cancer screening interventions among Asians - a systematic literature review**. *Asian Pacific Journal of Cancer Prevention* 2011, **12**(11):3133-3139.

Huey SJ, Jr., Polo AJ: **Evidence-based psychosocial treatments for ethnic minority youth**. *Journal of Clinical Child and Adolescent Psychology* 2008, **37**(1):262-301.

Huffman MD, Bhatnagar D: **Novel treatments for cardiovascular disease prevention**. *Cardiovascular Therapeutics* 2012, **30**(5):257-263.

Hutcheson AK, Piazza AJ, Knowlden AP: **Work site–based environmental interventions to reduce sedentary behavior**. *American Journal of Health Promotion* 2016:0890117116674681.

Hutcheson A, Wilson C: **Improving nutrition and physical activity in the workplace: a meta-analysis of intervention studies**. *Health Promotion International* 2010, **27**(2), 328-249.

Hynynen ST, van Stralen MM, Sniehotta FF, Araujo-Soares V, Hardeman W, Chinapaw MJM, Vasankari T, Hankonen N: **A systematic review of school-based interventions targeting physical activity and sedentary behaviour among older adolescents**. *International Review of Sport and Exercise Psychology* 2016, **9**(1):22-44.

Ickes MJ, McMullen J, Haider T, Sharma M: **Global school-based childhood obesity interventions: a review**. *International Journal of Environmental Research and Public Health* 2014, **11**(9):8940-8961.

Jackson C, Geddes R, Haw S, Frank J: **Interventions to prevent substance use and risky sexual behaviour in young people: a systematic review**. *Addiction* 2012, **107**(4):733-747.

Jackson KF, Hodge DR: **Native American youth and culturally sensitive interventions: a systematic review**. *Research on Social Work Practice* 2010, **20**(3):260-270.

Jackson KF, Hodge DR, Vaughn MG: **A meta-analysis of culturally sensitive interventions designed to reduce high-risk behaviors among African American youth**. *Journal of Social Service Research* 2010, **36**(3): 163-173.

Janer G, Sala M, Kogevinas M: **Health promotion trials at worksites and risk factors for cancer**. *Scandinavian Journal of Work Environment & Health* 2002, **28**(3):141-157.

Janssen MM, Mathijssen JJP, van Bon-Martens MJH, van Oers HAM, Garretsen HFL: **Effectiveness of alcohol prevention interventions based on the principles of social marketing: a systematic review**. *Substance Abuse Treatment Prevention and Policy* 2013, **8**: 18.

Johnson M, Everson-Hock E, Jones R, Woods HB, Payne N, Goyder E: **What are the barriers to primary prevention of type 2 diabetes in black and minority ethnic groups in the UK? A qualitative evidence synthesis**. *Diabetes Research and Clinical Practice* 2011, **93**(2):150-158.

Kahn-Marshall JL, Gallant MP: **Making Healthy Behaviors the Easy Choice for Employees: a review of the literature on environmental and policy changes in worksite health promotion**. *Health Education & Behavior* 2012, **39**(6):752-776.

Kardamanidis K, Martiniuk A, Ivers RQ, Stevenson MR, Thistlethwaite K: **Motorcycle rider training for the prevention of road traffic crashes**. *Cochrane Database of Systematic Reviews* 2010(10):CD005240.

Kavanagh J, Trouton A, Oakley A, Powell C: **A systematic review of the evidence for incentive schemes to encourage positive health and other social behaviours in young people**. In. London: EPPI-Centre, Social Science Research Unit, Institute of Education, University of London; 2006.

Kendrick D, Young B, Mason-Jones AJ, Ilyas N, Achana FA, Cooper NJ, Hubbard SJ, Sutton AJ, Smith S, Wynn P *et al*: **Home safety education and provision of safety equipment for injury prevention**. *Cochrane Database of Systematic Reviews* **2012**(9).

Kendrick D, Young B, Mason-Jones AJ, Ilyas N, Achana FA, Cooper NJ, Hubbard SJ, Sutton AJ, Smith S, Wynn P *et al*: **Home safety education and provision of safety equipment for injury prevention**. In: *Cochrane Database of Systematic Reviews.* **2012**.

Kidney, Winter, H.R, Khan, K.S, Gülmezoglu, A.M, Meads, C.A, Deeks *et al*: **Systematic review of effect of community-level interventions to reduce maternal mortality**. *BMC Pregnancy and Childbirth* 2009, **9:** 2.

Klassen TP, MacKay JM, Moher D, Walker A, Jones AL: **Community-based injury prevention interventions**. *Future of Children* 2000, **10**(1):83-110.

Knai C, Pomerleau J, Lock K, McKee M: **Getting children to eat more fruit and vegetables: a systematic review**. *Preventive Medicine* 2006, **42**(2):85-95.

Knowlden AP, Ickes MJ, Sharma M: **Systematic analysis of tobacco treatment interventions implemented in worksite settings**. *Journal of Substance Use* 2014, **19**(4):283-294.

Kong A, Tussing-Humphreys LM, Odoms-Young AM, Stolley MR, Fitzgibbon ML: **Systematic review of behavioural interventions with culturally adapted strategies to improve diet and weight outcomes in African American women**. *Obesity Reviews* 2014, **15**:62-92.

Korenbrot CC, Steinberg A, Bender C, Newberry S: **Preconception care: a systematic review**. *Maternal and Child Health Journal* 2002, **6**(2):75-88.

Korner-Bitensky N, Kua A, von Zweck C, Van Benthem K: **Older driver retraining: an updated systematic review of evidence of effectiveness**. *Journal of Safety Research* 2009, **40**(2):105-111.

Kristjansson E, Francis Damian K, Liberato S, Benkhalti Jandu M, Welch V, Batal M, Greenhalgh T, Rader T, Noonan E, Shea B *et al*: **Food supplementation for improving the physical and psychosocial health of socio-economically disadvantaged children aged three months to five years**. *Cochrane Database of Systematic Reviews* **2015**.

Krogsboll LT, PC KlLJrKGnjLGt: **General health checks in adults for reducing morbidity and mortality from disease**. *Cochrane Database of Systematic Reviews* **2012**.

Kua A, Korner-Bitensky N, Desrosiers J, Man-Son-Hing M, Marshall S: **Older driver retraining: a systematic review of evidence of effectiveness**. *Journal of Safety Research* 2007, **38**(1):81-90.

LaCroix JM, Snyder LB, Huedo-Medina TB, Johnson BT: **Effectiveness of mass media interventions for HIV prevention, 1986-2013: a meta-analysis**. *Journal of Acquired Immune Deficiency Syndromes* 2014, **66**(3):S329-S340.

Lang PO, Mendes A, Socquet J, Assir N, Govind S, Aspinall R: **Effectiveness of influenza vaccine in aging and older adults: comprehensive analysis of the evidence**. *Clinical Interventions in Aging* 2012, **7**:55-64.

Lawrence D, Graber JE, Mills SL, Meissner HI, Warnecke R: **Smoking cessation interventions in U.S. racial/ethnic minority populations: an assessment of the literature**. *Preventive Medicine* 2003, **36**(2):204-216.

Laws RA, St George AB, Rychetnik L, Bauman AE: **Diabetes prevention research: a systematic review of external validity in lifestyle interventions**. *American Journal of Preventive Medicine* 2012, **43**(2):205-214.

Leavy JE, Crawford G, Portsmouth L, Jancey J, Leaversuch F, Nimmo L, Hunt K: **Recreational Drowning Prevention Interventions for Adults, 1990-2012: a Review**. *Journal of Community Health* 2015, **40**(4):725-735.

Lehne G, Bolte G: **Impact of universal interventions on social inequalities in physical activity among older adults: an equity-focused systematic review**. *International Journal of Behavioral Nutrition and Physical Activity* 2017, **14**: 20.

Lemacks J, Wells BA, Ilich JZ, Ralston PA: **Interventions for improving nutrition and physical activity behaviors in adult African American populations: a systematic review, January 2000 through December 2011**. *Preventing Chronic Disease* 2013, **10**:E99.

Lemstra M, Bennett N, Nannapaneni U, Neudorf C, Warren L, Kershaw T, Scott C: **A systematic review of school-based marijuana and alcohol prevention programs targeting adolescents aged 10-15**. *Addiction Research & Theory* 2010, **18**(1):84-96.

Li R, Zhang P, Lawrence B, Chowdhury FM, Zhang X: **Cost-effectiveness of interventions for preventing and controlling diabetes mellitus: a systematic review**. *Diabetes Conference: 69th Annual Meeting of the American Diabetes Association New Orleans, LA United States Conference Start* 2009, **58**.

Liberato SC, Bailie R, Brimblecombe J: **Nutrition interventions at point-of-sale to encourage healthier food purchasing: a systematic review**. *BMC Public Health* 2014, **14**:919.

Ligthart K, Paulis WD, Djasmo D, Van Middelkoop M: **Multidisciplinary interventions in childhood obesity: A systematic review on quality of life and costeffectiveness**. *Obesity Facts* 2013, **6**:26-27.

Lindberg NM, Stevens VJ: **Review: weight-loss interventions with Hispanic populations**. *Ethnicity and Disease* 2007, **17**(2):397-402.

Lipstein EA, Vorono S, Browning MF, Green NS, Kemper AR, Knapp AA, Prosser LA, Perrin JM: **Systematic evidence review of newborn screening and treatment of severe combined immunodeficiency**. *Pediatrics* 2010, **125**(5):e1226-1235.

Lister-Sharp D, Chapman S, Stewart-Brown S, Sowden A: **Health promoting schools and health promotion in schools: two systematic reviews**. *Health Technology Assessment* 1999, **3**(22):iii-193.

Liu JJ, Wabnitz C, Davidson E, Bhopal RS, White M, Johnson MRD, Netto G, Sheikh A: **Smoking cessation interventions for ethnic minority groups - a systematic review of adapted interventions**. *Preventive Medicine* 2013, **57**(6):765-775.

Lopez L, Audisio Y, Berra S: **Efectividad de las intervenciones de base poblacional dirigidas a la prevención del sobrepeso en la población infantil y adolescente [In Spanish]. Effectiveness of population-based interventions on the prevention of overweight in children and adolescents**. *Medicina Clinica* 2010, **135**(10):462-469.

Lopez LM, Grey TW, Tolley EE, Chen M: **Brief educational strategies for improving contraception use in young people**. *Cochrane Database of Systematic Reviews* **2016**(3).

Lu M, Moritz S, Lorenzetti D, Sykes L, Straus S, Quan H: **A systematic review of interventions to increase breast and cervical cancer screening uptake among Asian women**. *BMC Public Health* 2012, **12**:413.

Ludolph R, Schulz PJ: **Does regulatory fit lead to more effective health communication? A systematic review**. *Social Science & Medicine* 2015, **128**:142-150.

Lyon-Callo SK, Boss LP, Lara M: **A review of potential state and local policies to reduce asthma disparities**. *CHEST* 2007, **132**:840S-852S.

Maglione MA, Stone EG, Shekelle PG: **Mass mailings have little effect on utilization of influenza vaccine among Medicare beneficiaries**. *American Journal of Preventive Medicine* 2002, **23**(1):43-46.

Magwood GS, Zapka J, Jenkins C: **A review of systematic reviews evaluating diabetes interventions: focus on quality of life and disparities**. *Diabetes Educator* 2008, **34**(2):242-265.

Maidment CD, Jones CR, Webb TL, Hathway EA, Gilbertson JM: **The impact of household energy efficiency measures on health: a meta-analysis**. *Energy Policy* 2014, **65**:583-593.

Mann L, Foley KL, Tanner AE, Sun CJ, Rhodes SD: **Increasing cervical cancer screening among US Hispanics/Latinas: a qualitative systematic review**. *Journal of Cancer Education* 2015, **30**(2):374-387.

Marcus AC, Crane LA: **A review of cervical cancer screening intervention research: Implications for public health programs and future research**. *Preventive Medicine* 1998, **27**:13-31.

Martens MP, Dams-O'Connor K, Beck NC: **A systematic review of college student-athlete drinking: prevalence rates, sport-related factors, and interventions**. *Journal of Substance Abuse Treatment* 2006, **31**(3), 305-316.

Martin J, Chater A, Lorencatto F: **Effective behaviour change techniques in the prevention and management of childhood obesity**. *International Journal of Obesity* 2013, **37**(10):1287-1294.

Masi CM, Blackman DJ, Peek ME: **Interventions to enhance breast cancer screening, a diagnosis, and treatment among racial and ethnic minority women**. *Medical Care Research and Review* 2007, **64**(5):S195S-242.

Mbuagbaw L, Medley N, Darzi A, Richardson M, Habiba G, Kesso, Ongolo-Zogo P: **Health system and community level interventions for improving antenatal care coverage and health outcomes**. *Cochrane Database of Systematic Reviews* **2015**(12).

McCalman J, Tsey K, Bainbridge R, Rowley K, Percival N, O'Donoghue L, Brands J, Whiteside M, Judd J: **The characteristics, implementation and effects of Aboriginal and Torres Strait Islander health promotion tools: a systematic literature search**. *BMC Public Health* 2014, **14**:712.

McCollum R, Gomez W, Theobald S, Taegtmeyer M: **How equitable are community health worker programmes and which programme features influence equity of community health worker services? A systematic review**. *BMC Public Health* 2016, **16**:419.

McDonald E, Bailie R, Brewster D, Morris P: **Are hygiene and public health interventions likely to improve outcomes for Australian Aboriginal children living in remote communities? A systematic review of the literature**. *BMC Public Health* 2008, **8**:153.

Melnyk BM: **The latest evidence to guide obesity prevention, policy, and clinical practice with overweight children and adolescents**. *Worldviews on Evidence-Based Nursing* 2009, **6**(1):44-48.

Meyer MRU, Perry CK, Sumrall JC, Patterson MS, Walsh SM, Clendennen SC, Hooker SP, Evenson KR, Goins KV, Heinrich KM *et al*: **Physical activity-related policy and environmental strategies to prevent obesity in rural communities: a systematic review of the literature, 2002-2013**. *Preventing Chronic Disease* 2016, **13**:150406

Mhurchu CN, Gorton D: **Nutrition labels and claims in New Zealand and Australia: a review of use and understanding**. *Australian and New Zealand Journal of Public Health* 2007, **31**(2):105-112.

Michie S, Jochelson K, Markham WA, Bridle C: **Low-income groups and behaviour change interventions: a review of intervention content, effectiveness and theoretical frameworks**. *Journal of Epidemiology & Community Health* 2009, **63**(8):610-622.

Mier N, Ory MG, Medina AA: **Anatomy of culturally sensitive interventions promoting nutrition and exercise in Hispanics: a critical examination of existing literature**. *Health Promotion Practice* 2010, **11**(4):541-554.

Monarrez-Espino J, Liu B, Greiner F, Bremberg S, Galanti R: **Systematic review of the effect of pictorial warnings on cigarette packages in smoking behavior**. *American Journal of Public Health* 2014, **104**(10):E11-E30.

Montano D, Hoven H, Siegrist J: **Effects of organisational-level interventions at work on employees' health: a systematic review**. *BMC Public Health* 2014, **14**:135.

Montano D, Hoven H, Siegrist J: **A meta-analysis of health effects of randomized controlled worksite interventions: Does social stratification matter?** *Scandinavian Journal of Work, Environment and Health* 2014, **40**(3):230-234.

Moodie C, Stead M, Bauld L, McNeill A, Angus K, Hinds K, Kwan I, Thomas J, Hastings G, O'Mara-Eves A: **Plain Tobacco Packaging: A Systematic Review**. 2012. Open University/University of Stirling.

Moore GF, Littlecott HJ, Turley R, Waters E, Murphy S: **Socioeconomic gradients in the effects of universal school-based health behaviour interventions: a systematic review of intervention studies**. *BMC Public Health* 2015, **15**:907.

Moreira MT, Smith LA, Foxcroft D: **Social norms interventions to reduce alcohol misuse in University or College students**. *Cochrane Database of Systematic Reviews* **2009**(3).

Morrison J, Pikhart H, Ruiz M, Goldblatt P: **Systematic review of parenting interventions in European countries aiming to reduce social inequalities in children's health and development**. *BMC Public Health* 2014, **14**(1):1040.

Morrow JB, Dallo FJ, Julka M: **Community-based colorectal cancer screening trials with multi-ethnic groups: a systematic review**. *Journal of Community Health* 2010, **35**(6):592-601.

Nagata JM, Hernandez-Ramos I, Kurup AS, Albrecht D, Vivas-Torrealba C, Franco-Paredes C: **Social determinants of health and seasonal influenza vaccination in adults >65 years: a systematic review of qualitative and quantitative data**. *BMC public health* 2013, **13**:388.

Naylor K, Ward J, Polite BN: **Interventions to improve care related to colorectal cancer among racial and ethnic minorities: a systematic review**. *Journal of General Internal Medicine* 2012, **27**(8):1033-1046.

Ndumbe-Eyoh S, Moffatt H: **Intersectoral action for health equity: a rapid systematic review**. *BMC Public Health* 2013, **13**:1056.

Niccolai LM, Hansen CE: **Practice- and community-based interventions to increase human papillomavirus vaccine coverage: a systematic review**. *JAMA Pediatrics* 2015, **169**(7):686-692.

Nierkens V, Hartman MA, Nicolaou M, Vissenberg C, Beune EJAJ, Hosper K, van Valkengoed IG, Stronks K: **Effectiveness of cultural adaptations of interventions aimed at smoking cessation, diet, and/or physical activity in ethnic minorities. A systematic review**. *Plos One* 2013, **8**(10):e73373.

Norris SL, Kansagara D, Bougatsos C, Fu R: **Screening adults for type 2 diabetes: a review of the evidence for the U.S. Preventive Services Task Force**. *Annals of Internal Medicine* 2008, **148**(11):855-868.

Odone A, Ferrari A, Spagnoli F, Visciarelli S, Shefer A, Pasquarella C: **Effectiveness of interventions that apply new media to improve vaccine uptake and vaccine coverage: a systematic review**. *Human Vaccines and Immunotherapeutics* 2015, **11**(1):72-82.

Oldroyd J, Burns C, Lucas P, Haikerwal A, Waters E: **The effectiveness of nutrition interventions on dietary outcomes by relative social disadvantage: a systematic review**. *Journal of Epidemiology & Community Health* 2008, **62**(7):573-579.

O'Malley AS, Gonzalez RM, Sheppard VB, Huerta E, Mandelblatt J: **Primary care cancer control interventions including Latinos - a review**. *American Journal of Preventive Medicine* 2003, **25**(3):264-271.

Palencia L, De Moortel D, Artazcoz L, Salvador-Piedrafita M, Puig-Barrachina V, Hagqvist E, Perez G, Ruiz ME, Trujillo-Aleman S, Vanroelen C *et al*: **Gender policies and gender inequalities in health in Europe: results of the SOPHIE project**. *International Journal of Health Services* 2017, **47**(1):61-82.

Pearson M, Garside R, Moxham T, Anderson R: **Preventing unintentional injuries to children in the home: a systematic review of the effectiveness of programmes supplying and/or installing home safety equipment**. *Health Promotion International* 2011, **26**(3):376-392.

Pearson M, Hunt H, Garside R, Moxham T, Peters J, Anderson R: **Preventing unintentional injuries to children under 15 years in the outdoors: a systematic review of the effectiveness of educational programs**. *Injury Prevention* 2012, **18**(2):113-123.

Peek ME, Cargill A, Huang ES: **Diabetes health disparities: a systematic review of health care interventions**. *Medical Care Research and Review* 2007, **64**(5 suppl):S101S-156.

Pennant M, Davenport C, Bayliss S, Greenheld W, Marshall T, Hyde C: **Community programs for the prevention of cardiovascular disease: a systematic review**. *American Journal of Epidemiology* 2010, **172**(5):501-516.

Perez LG, Arredondo EM, Elder JP, Barquera S, Nagle B, Holub CK: **Evidence-based obesity treatment interventions for Latino adults in the U.S.: a systematic review**. *American Journal of Preventive Medicine* 2013, **44**(5):550-560.

Petrie J, Bunn F, Byrne G: **Parenting programmes for preventing tobacco, alcohol or drugs misuse in children < 18: a systematic review**. *Health Education Research* 2007, **22**(2):177-191.

Platt L, Melendez-Torres GJ, O'Donnell A, Bradley J, Newbury-Birch D, Kaner E, Ashton C: **How effective are brief interventions in reducing alcohol consumption: do the setting, practitioner group and content matter? Findings from a systematic review and metaregression analysis**. *BMJ Open* 2016, **6**(8):e011473.

Pollack KM, Frattaroli S, Young JL, Dana-Sacco G, Gielen AC: **Motor vehicle deaths among American Indian and Alaska Native populations**. *Epidemiologic Reviews* 2012, **34**(1):73-88.

Pomerleau J, Lock K, Knai C, McKee M: **Interventions designed to increase adult fruit and vegetable intake can be effective: a systematic review of the literature**. *Journal of Nutrition* 2005, **135**(10):2486-2495.

Pontoppidan M, Klest SK, Patras J, Rayce SB: **Effects of universally offered parenting interventions for parents with infants: a systematic review**. *BMJ Open* 2016, **6**(9): e011706

Popova S, Giesbrecht N, Bekmuradov D, Patra J: **Hours and days of sale and density of alcohol outlets: impacts on alcohol consumption and damage: a systematic review**. *Alcohol and Alcoholism* 2009, **44**(5):500-516.

Powell LM, Chriqui JF, Khan T, Wada R, Chaloupka FJ: **Assessing the potential effectiveness of food and beverage taxes and subsidies for improving public health: a systematic review of prices, demand and body weight outcomes**. *Obesity Reviews* 2013, **14**(2):110-128.

Rawl SM, Menon U, Burness A, Breslau ES: **Interventions to promote colorectal cancer screening: an integrative review**. *Nursing Outlook* 2012, **60**(4), 172-181.

Roe L, Hunt P, Bradshaw H, Rayner M: **Health promotion interventions to promote healthy eating in the general population: a review**. London: Health Education Authority; 1997.

Sabatino SA, Lawrence B, Elder R, Mercer SL, Wilson KM, DeVinney B, Melillo S, Carvalho M, Taplin S, Bastani R *et al*: **Effectiveness of interventions to increase screening for breast, cervical, and colorectal cancers nine updated systematic reviews for the guide to community preventive services**. *American Journal of Preventive Medicine* 2012, **43**(1):97-118.

Sajid S, Kotwal AA, Dale W: **Interventions to improve decision making and reduce racial and ethnic disparities in the management of prostate cancer: a systematic review**. *Journal of General Internal Medicine* 2012, **27**(8):1068-1078.

Saunders KL: **Preventing obesity in pre-school children: a literature review**. *Journal of Public Health* 2007, **29**(4):368-375.

Sebastian-Ponce MI, Sanz-Valero J, Wanden-Berghe C: **Food labeling and the prevention of overweight and obesity: a systematic review [In Spanish]. Etiquetado y rotulacion de los alimentos en la prevencion del sobrepeso y la obesidad: Una revision sistematica**. *Cadernos de Saude Publica* 2011, **27**(11):2083-2094.

Secker-Walker RH, Gnich W, Platt S, Lancaster T: **Community interventions for reducing smoking among adults**. *The Cochrane Library* 2004(2).

Senore C, Giordano L, Bellisario C, Di Stefano F, Segnan N: **Population based cancer screening programmes as a teachable moment for primary prevention interventions. A review of the literature**. *Frontiers in Oncology* 2012, **2:** Article 45.

Seo D, Sa J: **A meta-analysis of psycho-behavioral obesity interventions among US multiethnic and minority adults**. *Preventive Medicine* 2008, **47**(6):573-82.

Serra C, Bonfill X, Vila MP, Pena CC: **Interventions for preventing tobacco smoking in public places** *Cochrane Database of Systematic Reviews* 2000(3).

Short MM, Mushquash CJ, Bédard M: **Interventions for motor vehicle crashes among Indigenous communities: Strategies to inform Canadian initiatives**. *Canadian Journal of Public Health* 2014, **105**(4):e296-305.

Si S, Moss JR, Sullivan TR, Newton SS, Stocks NP: **Effectiveness of general practice-based health checks: a systematic review and meta-analysis**. *British Journal of General Practice* 2014, **64**(618):E47-E53.

Smithson J, Garside R, Pearson M: **Barriers to, and facilitators of, the prevention of unintentional injury in children in the home: a systematic review and synthesis of qualitative research**. *Injury Prevention (1353-8047)* 2011, **17**(2):1-1.

Snethen JA, Broome ME, Treisman P, Castro E, Kelber ST: **Effective weight loss for children: a meta-analysis of intervention studies 2002-2015**. *Worldviews on Evidence-Based Nursing* 2016, **13**(4):294-302.

Teesson M, Newton NC, Barrett EL: **Australian school-based prevention programs for alcohol and other drugs: a systematic review**. *Drug and alcohol review* 2012, **31**:731-736.

Thomson CA, Ravia J: **A systematic review of behavioral interventions to promote intake of fruit and vegetables**. *Journal of the American Dietetic Association* 2011, **111**(10):1523-1535.

Thomson G, Wilson N, Howden-Chapman P: **Population level policy options for increasing the prevalence of smokefree homes**. *Journal of Epidemiology & Community Health* 2006, **60**(4):298-304.

Thomson H, Atkinson R, Petticrew M, Kearns A: **Do national urban regeneration programmes impact on health and the socio-economic determinants of health? A systematic review of UK regeneration programmes (1980-2004)**. *European Journal of Public Health* 2005, **15**:119.

Thomson H, Atkinson R, Petticrew M, Kearns A: **Do urban regeneration programmes improve public health and reduce health inequalities? A synthesis of the evidence from UK policy and practice (1980-2004)**. *Journal of Epidemiology & Community Health* 2006, **60**(2):108-115.

Tovar A, Renzaho AM, Guerrero AD, Mena N, Ayala GX: **A systematic review of obesity prevention intervention studies among immigrant populations in the US**. *Current Obesity Reports* 2014, **3**(2):206-222.

Towns C, Cooke M, Rysdale L, Wilk P: **Healthy weights interventions in Aboriginal children and youth: a review of the literature**. *Canadian Journal of Dietetic Practice & Research* 2014, **75**(3), 125-131.

Turner C, Spinks A, McClure RJ, Nixon J: **Community-based interventions for the prevention of burns and scalds in children**. Cochrane Database of Systematic Reviews 2004.

Valdivia Espino JN, Guerrero N, Rhoads N, Simon NJ, Escaron AL, Meinen A, Nieto FJ, Martinez-Donate AP: **Community-based restaurant interventions to promote healthy eating: a systematic review**. *Preventing Chronic Disease* 2015, **12**:E78.

van 't Riet J: **Sales effects of product health information at points of purchase: a systematic review**. *Public Health Nutrition* 2013, **16(3)**:418-429.

van Voorhees BW, Walters AE, Prochaska M, Quinn MT: **Reducing health disparities in depressive disorders outcomes between non-hispanic whites and ethnic minorities: a call for pragmatic strategies over the life course**. *Medical Care Research and Review* 2007, **64**(5 suppl):S157S-194.

Vernon SW, McQueen A, Tiro JA, del Junco DJ: **Interventions to promote repeat breast cancer screening with mammography: a systematic review and meta-analysis**. *Journal of the National Cancer Institute* 2010, **102**(14):1023-1039.

Verweij LM, Coffeng J, van Mechelen W, Proper KI: **Meta-analyses of workplace physical activity and dietary behaviour interventions on weight outcomes**. *Obesity Reviews* 2011, **12**(6):406-429.

Vidourek RA, King KA: **Effectiveness of nutrition programs in increasing healthy eating behaviors among low income women**. *Californian Journal of Health Promotion* 2008, **6**(1):57-72.

Viswesvaran C, Schmidt FL: **A metaanalytic comparison of the effectiveness of smoking cessation methods**. *Journal of Applied Psychology* 1992, **77**(4):554-561.

Walker RE, Gordon M: **The use of lifestyle and behavioral modification approaches in obesity interventions for Black women: a literature review**. *Health Education & Behavior* 2014, **41**(3):242-258.

Wang D, Stewart D: **The implementation and effectiveness of school-based nutrition promotion programmes using a health-promoting schools approach: a systematic review**. *Public Health Nutrition* 2013, **16**(6):1082-1100.

Ward K, Chow MYK, King C, Leask J: **Strategies to improve vaccination uptake in Australia, a systematic review of types and effectiveness**. *Australian and New Zealand Journal of Public Health* 2012, **36**(4):369-377.

Ward SH, Lin K, Meyer B, Bass SB, Parameswaran L, Gordon TF, Ruzek SB: **Increasing colorectal cancer screening among African Americans, linking risk perception to interventions targeting patients, communities and clinicians**. *Journal of the National Medical Association* 2008, **100**(6):748-758.

Waters E, de Silva-Sanigorski A, Hall BJ, Brown T, Campbell KJ, Gao Y, Armstrong R, Prosser L, Summerbell CD: **Interventions for preventing obesity in children**. *Cochrane Database of Systematic Reviews* 2011(12).

Webb MS: **Treating tobacco dependence among African Americans: a meta-analytic review**. *Health Psychology* 2008, **27(3 Supplement)**:S271-S282.

Webb MS, Rodriguez-Esquivel D, Baker EA: **Smoking cessation interventions among Hispanics in the United States: a systematic review and mini meta-analysis**. *American Journal of Health Promotion* 2010, **25**(2):109-118.

Wells J, Barlow J, Stewart-Brown S: **A systematic review of universal approaches to mental health promotion in schools**. *Health Education* 2003, **103**(4):197-220.

Wilson N, Thomson G: **Tobacco tax as a health protecting policy: a brief review of the New Zealand evidence**. *New Zealand Medical Journal* 2005, **118**(1213):U1403.

Windle G, Russell I, Linck P, Woods R, Hughes D: **A systematic review of the effectiveness of public health interventions aimed at promoting mental well-being in older people**. *Gerontologist* 2008, **48**:740.

Wolfenden L, Wyse R, Nichols M, Allender S, Millar L, McElduff P: **A systematic review and meta-analysis of whole of community interventions to prevent excessive population weight gain**. *Preventive Medicine* 2014, **62**:193-200.

Wolfenden L, Wyse Rebecca J, Britton Ben I, Campbell Karen J, Hodder Rebecca K, Stacey Fiona G, McElduff P, James Erica L: **Interventions for increasing fruit and vegetable consumption in children aged 5 years and under**. *Cochrane Database of Systematic Reviews* 2012(11).

Yabroff KR, Mangan P, Mandelblatt J: **Effectiveness of interventions to increase Papanicolaou smear use**. *The Journal of the American Board of Family Practice* 2003, **16**(3):188-203.

Yeoh B, Woolfenden S, Lanphear B, Ridley Greta F, Livingstone N, Jorgensen E: **Household interventions for preventing domestic lead exposure in children**. *Cochrane Database of Systematic Reviews* 2014.

Yeung CA: **A systematic review of the efficacy and safety of fluoridation**. *Evidence-based dentistry* 2008, **9**(2):39-43.

Yeung CA, Chong Lee Y, Glenny A-M: **Fluoridated milk for preventing dental caries**. *Cochrane Database of Systematic Reviews* 2015(9).

Young I, Waddell L, Harding S, Greig J, Mascarenhas M, Sivaramalingam B, Pham MT, Papadopoulos A: **A systematic review and meta-analysis of the effectiveness of food safety education interventions for consumers in developed countries**. *BMC Public Health* 2015, **15**:822.

Zeh P, Sandhu H, Cannaby A, Sturt J: **The impact of culturally competent diabetes care interventions for improving diabetes-related outcomes in ethnic minority groups: A systematic review**. *Diabetic Medicine* 2012, **29**(10):1237-1252.

<u>**Inappropriate study design used**</u>

Aldridge RW, Yates TA, Zenner D, White PJ, Abubakar I, Hayward AC: **Pre-entry screening programmes for tuberculosis in migrants to low-incidence countries: A systematic review and meta-analysis**. *The Lancet Infectious Diseases* 2014, **14**(12):1240-1249.

Bell K, McCullough L, DeVries K, Greaves L, Jategaonkar N: **NICE Rapid review. Workplace policies and interventions for Smoking Cessation**. 2007:1-122.

Christou A, Katzenellenbogen JM, Thompson SC: **Australia's national bowel cancer screening program: does it work for indigenous Australians?** *BMC Public Health* 2010, **10**:373.

Dyda A, Karki S, Hayen A, MacIntyre CR, Menzies R, Banks E, Kaldor JM, Liu B: **Influenza and pneumococcal vaccination in Australian adults: a systematic review of coverage and factors associated with uptake**. *BMC Infectious Diseases* 2016, **16**(1):515.

Guillaumier A, Bonevski B, Paul C: **Anti-tobacco mass media and socially disadvantaged groups: a systematic and methodological review**. *Drug and Alcohol Review* 2012, **31**(5):698-708.

Hughes N, Arora M, Grills N: **Perceptions and impact of plain packaging of tobacco products in low and middle income countries, middle to upper income countries and low-income settings in high-income countries: a systematic review of the literature**. *BMJ Open* 2016, **6**(3):e010391

Klinkenberg E, Manissero D, Semenza JC, Verver S: **Migrant tuberculosis screening in the EU/EEA: yield, coverage and limitations**. *European Respiratory Journal* 2009, **34**(5):1180-1189.

Levy DT, Friend KB: **The effects of clean indoor air laws: what do we know and what do we need to know?** *Health Education Research* 2003, **18**(5):592-609.

Richardson L, Hemsing N, Greaves L, Assanand S, Allen P, McCullough L, Bauld L, Humphries K, Amos A: **Preventing smoking in young people: a systematic review of the impact of access interventions**. *International Journal of Environmental Research and Public Health* 2009, **6**(4):1485.

Rowe RE, Garcia J, Davidson LL: **Social and ethnic inequalities in the offer and uptake of prenatal screening and diagnosis in the UK: a systematic review**. *Public Health* 2004, **118**(3):177-189.

Thow A, Downs SJS: **A systematic review of the effectiveness of food taxes and subsidies to improve diets: understanding the recent evidence**. *Nutrition Reviews* 2014, **72(9)**:551-565.

Wagenaar AC, Tobler AL, Komro KA: **Effects of alcohol tax and price policies on morbidity and mortality: a systematic review**. *American Journal of Public Health* 2010, **100**(11):2270-2278.

Wang L, Zhong BQ, Vardoulakis S, Zhang FY, Pilot E, Li YH, Yang LS, Wang WY, Krafft T: **Air quality strategies on public health and health equity in Europe-a systematic review**. *International Journal of Environmental Research and Public Health* 2016, **13**(12).

<u>No health outcomes of relevance are described.</u>

Anderson R: **The role of community-based programs in addressing health disparities as it relates to breast and cervical cancer in African American women: a systematic review of studies**. *PhD Thesis The University of Alabama at Birmingham Thesis* 2009, **69**:4646.

Angell BJ, Muhunthan J, Irving M, Eades S, Jan S: **Global systematic review of the cost-effectiveness of indigenous health interventions**. *PloS one* 2014, **9**(11):e111249.

Attree P: **A critical analysis of UK public health policies in relation to diet and nutrition in low-income households**. *Maternal and Child Nutrition* 2006, **2**(2):67-78.

Bauermeister JA, Tross S, Ehrhardt AA: **A Review of HIV/AIDS system-level interventions**. *Aids and Behavior* 2009, **13**(3):430-448.

Beauchamp A, Peeters A, Tonkin A, Turrell G: **Best practice for prevention and treatment of cardiovascular disease through an equity lens: a review**. *European Journal of Cardiovascular Prevention & Rehabilitation* 2010, **17**(5):599-606.

Blankenship KM, Bray SJ, Merson MH: **Structural interventions in public health**. *Aids* 2000, **14**(11 SUPPL.):S11-S21.

Campbell ME, Gardner CE, Dwyer JJ, Isaacs SM, Krueger PD, Ying JY: **Effectiveness of public health interventions in food safety: a systematic review**. *Canadian Journal of Public Health* 1998, **89**(3):197-202.

Cobiac L, Vos T, Doran C, Wallace A: **Cost-effectiveness of interventions to prevent alcohol-related disease and injury in Australia**. *Addiction* 2009, **104**(10):1646-1655.

Collins PA, Hayes MV: **The role of urban municipal governments in reducing health inequities: a meta-narrative mapping analysis**. *International Journal for Equity in Health* 2010, **9:**13.

Cowley S, Whittaker K, Malone M, Donetto S, Grigulis A, Maben J: **Why health visiting? Examining the potential public health benefits from health visiting practice within a universal service: a narrative review of the literature**. *International Journal of Nursing Studies* 2015, **52**(1):465-480.

Everson-Hock ES, Johnson M, Jones R, Woods HB, Goyder E, Payne N, Chilcott J: **Community-based dietary and physical activity interventions in low socioeconomic groups in the UK: A mixed methods systematic review**. *Preventive Medicine* 2013, **56**(5):265-272 268p.

Ganann R, Fitzpatrick-Lewis D, Ciliska D, Peirson LJ, Warren RL, Fieldhouse P, Delgado-Noguera MF, Tort S, Hams SP, Martinez-Zapata MJ *et al*: **Enhancing nutritional environments through access to fruit and vegetables in schools and homes among children and youth: a systematic review**. *BMC Research Notes* 2014, **7**(1):422.

Gordon LG, Rowell D: **Health system costs of skin cancer and cost-effectiveness of skin cancer prevention and screening: A systematic review**. *European Journal of Cancer Prevention* 2015, **24**(2):141-149.

Greaves L, Johnson J, Bottorff J, Kirkland S, Jategaonkar N, McGowan M, McCullough L, Battersby L: **What are the effects of tobacco policies on vulnerable populations?** *Canadian Journal of Public Health* 2006, **97**(4):310-315.

Hahn RA, Barnett WS, Knopf JA, Truman BI, Johnson RL, Fielding JE, Muntaner C, Jones CP, Fullilove MT, Hunt PC: **Early childhood education to promote health equity: a community guide systematic review**. *Journal of Public Health Management and Practice* 2016, **22**(5):E1-E8.

Huffman MD, Galloway JM: **Cardiovascular health in Indigenous communities: successful programs**. *Heart Lung and Circulation* 2010, **19**(5-6):351-360.

Ivers RG: **A review of tobacco interventions for indigenous Australians**. *Australian and New Zealand Journal of Public Health* 2003, **27**(3):294-299.

Kristjansson EA, Robinson V, Petticrew M, MacDonald B, Krasevec J, Janzen L, Greenhalgh T, Wells G, MacGowan J, Farmer A *et al*: **School feeding for improving the physical and psychosocial health of disadvantaged students**. *Cochrane Database of Systematic Reviews* 2007(1).

Lachenmeier DW, Taylor BJ, Rehm J: **Alcohol under the radar: Do we have policy options regarding unrecorded alcohol?** *International Journal of Drug Policy* 2011, **22**(2):153-160.

Leao LL, De Aguiar MJB: **Newborn screening: What pediatricians should know**. *Jornal de Pediatria* 2008, **84**(4 SUPPL.):S80-S90.

Martin-Moreno JM, Harris ME, Breda J, Moller L, Alfonso-Sanchez JL, Gorgojo L: **Enhanced labelling on alcoholic drinks: reviewing the evidence to guide alcohol policy**. *European Journal of Public Health* 2013, **23**(6):1082-1087.

Mosdol A, Lidal IB, Straumann GH, Vist GE: **Targeted mass media interventions promoting healthy behaviours to reduce risk of non-communicable diseases in adult, ethnic minorities**. *Cochrane Database of Systematic Reviews* 2017(2).

Niebylski ML, Lu T, Campbell NRC, Arcand J, Schermel A, Hua D, Yeates KE, Tobe SW, Twohig PA, L'Abbe MR *et al*: **Healthy food procurement policies and their impact**. *International Journal of Environmental Research and Public Health* 2014, **11**(3):2608-2627.

O'Dwyer LA, Baum F, Kavanagh A, Macdougall C: **Do area-based interventions to reduce health inequalities work: a systematic review of evidence**. *Critical Public Health* 2007, **17**(4):317-335.

Pizzo G, Piscopo MR, Pizzo I, Giuliana G: **Community water fluoridation and caries prevention: A critical review**. *Clinical Oral Investigations* 2007, **11**(3):189-193.

Pons-Vigues M, Diez E, Morrison J, Salas-Nicas S, Hoffmann R, Burstrom B, van Dijk JP, Borrell C: **Social and health policies or interventions to tackle health inequalities in European cities: a scoping review**. *BMC Public Health* 2014, **14**:198.

Rosian-Schikuta I, Froschl B, Habl C, Sturzlinger H: **The Measels-Mumps-Rubella Vaccination from a health political and economical point of view**. *GMS Health Technology Assessment* 2007, **3**:Doc12.

Smith SC, Stephen AM, Dombrow C, MacQuarrie D: **Food information programs: a review of the literature**. *Canadian Journal of Dietetic Practice & Research* 2002, **63**(2):55-60.

van den Berg M, de Wit GA, Vijgen SM, Busch MC, Schuit AJ: **Cost-effectiveness of prevention: opportunities for public health policy in the Netherlands. [Dutch] Kosteneffectiviteit van preventie: kansen voor het Nederlandse volksgezondheidsbeleid**. *Nederlands Tijdschrift voor geneeskunde* 2008, **152**(23):1329-1334.

Walter U, Krauth C, Wienold M, Dreier M, Bantel S, Droste S: **Interventions for increasing uptake in screening programmes**. *GMS Health Technology Assessment* 2006, **2**:Doc16.

Welsh J, Strazdins L, Ford L, Friel S, O'Rourke K, Carbone S, Carlon L: **Promoting equity in the mental wellbeing of children and young people: a scoping review**. *Health Promotion International* 2015, **30**:36-76.

Welte R, Jager H, Postma MJ: **Cost-effectiveness of screening for genital Chlamydia trachomatis**. *Expert Review of Pharmacoeconomics and Outcomes Research* 2001, **1**(2):145-156.

Wigham S, Ternent L, Bryant A, Robalino S, Sniehotta FF, Adams J: **Parental financial incentives for increasing preschool vaccination uptake: systematic review**. *Pediatrics* 2014, **134**(4):E1117-E1128.

Yancey AK, Kumanyika SK, Ponce NA, McCarthy WJ, Fielding JE, Leslie JP, Akbar J: **Population-based interventions engaging communities of color in healthy eating and active living: a review**. *Preventing Chronic Disease* 2004, **1**(1):A09.

Yang L, Sahlqvist S, McMinn A, Griffin SJ, Ogilvie D: **Interventions to promote cycling: systematic review**. *BMJ* 2010, **341**:c5293.

No health inequality data is presented.

Abdul-Quader AS, Feelemyer J, Modi S, Stein ES, Briceno A, Semaan S, Horvath T, Kennedy GE, Des Jarlais DC: **Effectiveness of structural-level needle/syringe programs to reduce HCV and HIV Infection among people who inject drugs: a systematic review**. *AIDS and Behavior* 2013, **17**(9):2878-2892.

Aguiar M, Andronis L, Pallan M, Hogler W, Frew E: **Preventing vitamin D deficiency (VDD): a systematic review of economic evaluations**. *European Journal of Public Health* 2017, **27**(2):292-301.

Apollonio DE, Wolfe N, Bero LA: **Realist review of policy intervention studies aimed at reducing exposures to environmental hazards in the United States**. *BMC Public Health* 2016, **16**(1):822.

Atusingwize E, Lewis S, Langley T: **Economic evaluations of tobacco control mass media campaigns: a systematic review**. *Tobacco Control* 2015, **24**(4):320-327.

Audrey S, Batista-Ferrer H: **Healthy urban environments for children and young people: a systematic review of intervention studies**. *Health & Place* 2015, **36**:97-117.

Babb S, McNeil C, Kruger J, Tynan MA: **Secondhand smoke and smoking restrictions in casinos: a review of the evidence**. *Tobacco Control* 2015, **24**(1):11-17.

Babigumira JB, Morgan I, Levin A: **Health economics of rubella: a systematic review to assess the value of rubella vaccination**. *BMC Public Health* 2013, **13**:406.

Barlow P, McKee M, Basu S, Stuckler D: **The health impact of trade and investment agreements: a quantitative systematic review and network co-citation analysis**. *Globalization and Health* 2017, **13**:13.

Been J, Nurmatov UB, Cox B, Nawrot TS, van Schayck CP, Sheikh A: **Effect of smoke-free legislation on perinatal and child health: a systematic review and meta-analysis**. *Lancet* 2014, **383**(9928):1549-1560.

Bergen G, Pitan A, Qu S, Shults RA, Chattopadhyay SK, Elder RW, Sleet DA, Coleman HL, Compton RP, Nichols JL *et al*: **Publicized sobriety checkpoint programs: a community guide systematic review**. *American Journal of Preventive Medicine* 2014, **46**(5):529-539.

Berger I, Mooney-Somers J: **Smoking cessation programs for Lesbian, Gay, Bisexual, Transgender, and Intersex People: a content-based systematic review**. *Nicotine & Tobacco Research* 2017, **19**(12), 1408-1417.

Bolier L, Voorham L, Monshouwer K, van Hasselt N, Bellis M: **Alcohol and drug prevention in nightlife settings: a review of experimental studies**. *Substance Use & Misuse* 2011, **46**(13):1569-1591.

Booth A, Meier P, Stockwell T, Sutton A, Wilkinson A, Wong R: **Independent review of the effects of alcohol pricing and promotion. Part A: systematic reviews**. 2008. The University of Sheffield.

Brainard JS, Ford JA, Steel N, Jones AP: **A systematic review of health service interventions to reduce use of unplanned health care in rural areas**. *Journal of Evaluation in Clinical Practice* 2016, **22**(2):145-155.

Brennan I, Moore SC, Byrne E, Murphy S: **Interventions for disorder and severe intoxication in and around licensed premises, 1989-2009**. *Addiction* 2011, **106**(4):706-713.

Bryden A, Roberts B, McKee M, Petticrew M: **A systematic review of the influence on alcohol use of community level availability and marketing of alcohol**. *Health & Place* 2012, **18**(2):349-357.

Bunn F, Collier T, Frost C, Ker K, Roberts I, Wentz R: **Area-wide traffic calming for preventing traffic related injuries**. *Cochrane Database of Systematic Reviews* 2003(1):CD003110.

Cabrera E, M A, Veerman JL, Tollman SM, Bertram MY, Hofman KJ: **Evidence that a tax on sugar sweetened beverages reduces the obesity rate: a meta-analysis**. *BMC Public Health* 2013, **13**:1072.

Campbell CA, Hahn RA, Elder R, Brewer RD, Chattopadhyay S, Fielding J, Naimi TS, Toomey TL, Lawrence B, Middleton JC: **The effectiveness of limiting alcohol outlet density as a means of reducing excessive alcohol consumption and alcohol-related harms**. *American Journal of Preventive Medicine* 2009, **37**(6):556-569.

Capella ML, Taylor CR, Webster C: **The effect of cigarette advertising bans on consumption - a meta-analysis**. *Journal of Advertising* 2008, **37**(2):7-18.

Carson KV, Brinn MP, Labiszewski NA, Esterman AJ, Chang AB, Smith BJ: **Community interventions for preventing smoking in young people**. *Cochrane Database of Systematic Reviews* 2011(7):140.

Cashman Clodagh M, Ruotsalainen Jani H, Greiner Birgit A, Beirne Paul V, Verbeek Jos H: **Alcohol and drug screening of occupational drivers for preventing injury**. *Cochrane Database of Systematic Reviews* 2009 (2): *CD006566.*

Cavill N, Kahlmeier S, Rutter H, Racioppi F, Oja P: **Economic analyses of transport infrastructure and policies including health effects related to cycling and walking: a systematic review (vol 15, pg 291, 2008)**. *Transport Policy* 2009, **16**(1):46.

Christoforou A, Trieu K, Land MA, Bolam B, Webster J: **State-level and community-level salt reduction initiatives: a systematic review of global programmes and their impact**. *Journal of Epidemiology and Community Health* 2016, **70**(11):1140-1150.

Coffman S: **Promotion of safety helmets for child bicyclists: 2002 update**. *Online Journal of Knowledge Synthesis for Nursing* 2002, **9**(1):11.

Cullerton K, Donnet T, Lee A, Gallegos D: **Using political science to progress public health nutrition: a systematic review**. *Public Health Nutrition* 2016, **19**(11):2070-2078.

Dangour AD, Hawkesworth S, Shankar B, Watson L, Srinivasan CS, Morgan EH, Haddad L, Waage J: **Can nutrition be promoted through agriculture-led food price policies? A systematic review**. *BMJ Open* 2013, **3** (6):e002937.

Das JK, Salam RA, Arshad A, Lassi ZS, Bhutta ZA: **Systematic review and meta-analysis of interventions to improve access and coverage of adolescent immunizations**. *Journal of Adolescent Health* 2016, **59**(4):S40-S48.

Delgado-Noguera M, Tort S, Martinez-Zapata MJ, Bonfill X: **Primary school interventions to promote fruit and vegetable consumption: A systematic review and meta-analysis**. *Preventive Medicine* 2011, **53**(1-2):3-9.

DiFranza JR: **Which interventions against the sale of tobacco to minors can be expected to reduce smoking?** *Tobacco Control* 2012, **21**(4):436.

Dowswell T, Towner EM, Simpson G, Jarvis SN: **Preventing childhood unintentional injuries-what works? A literature review**. *Injury Prevention* 1996, **2**(2):140-149.

Drolet M, Benard E, Boily MC, Ali H, Baandrup L, Bauer H, Beddows S, Brisson J, Brotherton JM, Cummings T *et al*: **Population-level impact and herd effects following human papillomavirus vaccination programmes: a systematic review and meta-analysis**. *The Lancet Infectious Diseases* 2015, **15**(5):565-580.

Duailibi S, Laranjeira R: **Alcohol-related public policies**. *Revista De Saude Publica* 2007, **41**(5):839-848.

Egan M, Petticrew M, Ogilvie D, Hamilton V, Drever F: **'Profits before people'? A systematic review of the health and safety impacts of privatising public utilities and industries in developed countries**. *Journal of Epidemiology & Community Health* 2007, **61**(10):862-870 869p.

Elder RW, Lawrence B, Ferguson A, Naimi TS, Brewer RD, Chattopadhyay SK, Toomey TL, Fielding JE: **The effectiveness of tax policy interventions for reducing excessive alcohol consumption and related harms**. *American Journal of Preventive Medicine* 2010, **38**(2):217-229.

Elder RW, Shults RA, Sleet DA, Nichols JL, Thompson RS, Rajab W: **Effectiveness of mass media campaigns for reducing drinking and driving and alcohol-involved crashes: a systematic review**. *American Journal of Preventive Medicine* 2004, **27**(1):57-65.

Elder RW, Shults RA, Sleet DA, Nichols JL, Zaza S, Thompson RS: **Effectiveness of sobriety checkpoints for reducing alcohol-involved crashes**. *Traffic Injury Prevention* 2002, **3**(4):266-274.

Elliott B: **Road safety mass media campaigns: a meta analysis**. In. Federal Office of Road Safety (Canberra); 1993.

Erke A, Goldenbeld C, Vaa T: **The effects of drink-driving checkpoints on crashes-a meta-analysis**. *Accident Analysis and Prevention* 2009, **41**(5):914-923.

Fayter D, Nixon J, Hartley S, Rithalia A, Butler G, Rudolf M, Glasziou P, Bland M, Stirk L, Westwood M: **A systematic review of the routine monitoring of growth in children of primary school age to identify growth-related conditions**. *Health Technology Assessment* 2007, **11**(40):1-182 182p.

Fichtenberg CM, Glantz SA: **Effect of smoke-free workplaces on smoking behaviour: systematic review**. *British Medical Journal* 2002, **325**(7357):188-191.

Fichtenberg CM, Glantz SA: **Youth access interventions do not affect youth smoking**. *Pediatrics* 2002, **109**(6):1088-1092.

Foss RD, Evenson KR: **Effectiveness of graduated driver licensing in reducing motor vehicle crashes**. *American Journal of Preventive Medicine* 1999, **16**(1):47-56.

Frazer K, McHugh J, Callinan JE, Kelleher C: **Impact of institutional smoking bans on reducing harms and secondhand smoke exposure**. *Cochrane Database of Systematic Reviews* 2016(5).

Friedman AL, Kachur RE, Noar SM, McFarlane M: **Health communication and social marketing campaigns for sexually transmitted disease prevention and control: what is the evidence of their effectiveness?** *Sexually Transmitted Diseases* 2016, **43**:S83-S101.

Friend K, Levy DT: **Reductions in smoking prevalence and cigarette consumption associated with mass-media campaigns**. *Health Education Research* 2002, **17**(1):85-98.

Golechha M: **Health promotion methods for smoking prevention and cessation: a comprehensive review of effectiveness and the way forward**. *International Journal of Preventive Medicine* 2016, **7**:7.

Goss CW, Van Bramer LD, Gliner JA, Porter TR, Roberts IG, DiGuiseppi C: **Increased police patrols for preventing alcohol-impaired driving**. *Cochrane Database of Systematic Reviews* 2008(4).

Gøtzsche PC JK: **Screening for breast cancer with mammography (Review)**. *Cochrane Database of Systematic Review* 2013.

Grilli R, Ramsay C, Minozzi S: **Mass media interventions: effects on health services utilisation**. *Cochrane Database of Systematic Reviews* 2002.

Gyllensvard H: **Cost-effectiveness of injury prevention - a systematic review of municipality based interventions**. *Cost Effectiveness and Resource Allocation* 2010, **8:**17.

Hahn RA, Kuzara JL, Elder R, Brewer RD, Chattopadhyay S, Fielding J, Naimi TS, Toomey TL, Middleton JC, Lawrence B *et al*: **Effectiveness of policies restricting hours of alcohol sales in preventing excessive alcohol consumption and related harms**. *American Journal of Preventive Medicine* 2010, **39**(6):590-604.

Hahn RA, Middleton JC, Elder R, Brewer R, Fielding J, Naimi TS, Toomey TL, Chattopadhyay S, Lawrence B, Campbell CA *et al*: **Effects of Alcohol Retail Privatization on Excessive Alcohol Consumption and Related Harms A Community Guide Systematic Review**. *American Journal of Preventive Medicine* 2012, **42**(4):418-427.

Hanney S, Greenhalgh T, Blatch-Jones A, Glover M, Raftery J: **The impact on healthcare, policy and practice from 36 multi-project research programmes: findings from two reviews**. *Health Research Policy and Systems* 2017, **15**:26.

Hart KM, Demarco RF: **Primary prevention of skin cancer in children and adolescents: a review of the literature**. *Journal of Pediatric Oncology Nursing* 2008, **25**(2):67-78.

Health Quality O: **Screening mammography for women aged 40 to 49 years at average risk for breast cancer: an evidence-based analysis**. *Ontario Health Technology Assessment Series* 2007, **7**(1):1-32.

Heath GW, Brownson RC, Kruger J, Miles R, Powell KE, Ramsey LT: **The effectiveness of urban design and land use and transport policies and practices to increase physical activity: a systematic review**. *Journal of Physical Activity & Health* 2006, **3**(Suppl1):S55-S76.

Hersey JC, Wohlgenant KC, Arsenault JE, Kosa KM, Muth MK: **Effects of front-of-package and shelf nutrition labeling systems on consumers**. *Nutrition Reviews* 2013, **71**(1):1-14.

Holub CK, Lobelo F, Mehta SM, Sanchez Romero LM, Arredondo EM, Elder JP: **School-wide programs aimed at obesity among latino youth in the United States: a review of the evidence**. *Journal of School Health* 2014, **84**(4):239-246.

Hope SF, Webster J, Trieu K, Pillay A, Ieremia M, Bell C, Snowdon W, Neal B, Moodie M: **A systematic review of economic evaluations of population-based sodium reduction interventions**. *Plos One* 2017, **12**(3): e0173600.

Hopkins DP, Razi S, Leeks KD, Priya Kalra G, Chattopadhyay SK, Soler RE: **Smokefree policies to reduce tobacco use. A systematic review**. *American Journal of Preventive Medicine* 2010, **38**(2 Suppl):S275-289.

Hyde TB, Dentz H, Wang SA, Burchett HE, Mounier-Jack S, Mantel CF, New Vaccine Introduction Impact Published Literature Working Group: **The impact of new vaccine introduction on immunization and health systems: a review of the published literature**. *Vaccine* 2012, **30**(45):6347-6358.

Jaakkola MS, Jaakkola JJ: **Impact of smoke-free workplace legislation on exposures and health: possibilities for prevention**. *European Respiratory Journal* 2006, **28**(2):397-408.

Jacob V, Chattopadhyay SK, Elder RW, Robinson MN, Tansil KA, Soler RE, Labre MP, Mercer SL: **Economics of mass media health campaigns with health-related product distribution: a community guide systematic review**. *American Journal of Preventive Medicine* 2014, **47**(3):348-359.

Jaime PC, Lock K: **Do school based food and nutrition policies improve diet and reduce obesity?** *Preventive Medicine* 2009, **48**(1):45-53.

Jefferson T, Smith S, Demicheli V, Harnden A, Rivetti A, Di Pietrantonj C: **Assessment of the efficacy and effectiveness of influenza vaccines in healthy children: systematic review**. *Lancet* 2005, **365**(9461):773-780.

Jepson R, Clegg A, Forbes C, Lewis R, Sowden A, Kleijnen J: **The determinants of screening uptake and interventions for increasing uptake: a systematic review**. *Health Technology Assessment* 2000, 4(14): i-vii, 1-133.

Jones L, Hughes K, Atkinson AM, Bellis MA: **Reducing harm in drinking environments: a systematic review of effective approaches**. *Health & Place* 2011, **17**(2):508-518.

Kabir Z, Alpert HR, Goodman PG, Haw S, Behm I, Connolly GN, Gupta PC, Clancy L: **Effect of smoke-free home and workplace policies on second-hand smoke exposure levels in children: an evidence summary**. *Paediatric Health* 2010, **4**(4):391-403.

Karkhaneh M, Kalenga JC, Hagel BE, Rowe BH: **Effectiveness of bicycle helmet legislation to increase helmet use: a systematic review**. *Injury Prevention* 2006, **12**(2):76-82.

Kennedy SM, Davis SP, Thorne SL: **Smoke-free policies in U.S. prisons and jails: a review of the literature**. *Nicotine and Tobacco Research* 2015, **17**(6):629-635.

Ker K, Chinnock P: **Interventions in the alcohol server setting for preventing injuries**. *Cochrane Database of Systematic Reviews* 2008(3).

Killoran A, Canning U, Doyle N, Sheppard L: **Review of effectiveness of laws limiting blood alcohol concentration levels to reduce alcohol-related road injuries and deaths**. 2010. Centre for Public Health Excellance (NICE).

Klassen TP, MacKay JM, Moher D, Walker A, Jones AL: **Community-based injury prevention interventions**. *Future Child* 2000, **10**(1): 83-110.

Krogsboll LT, PC KlLJrKGnjLGt: **General health checks in adults for reducing morbidity and mortality from disease**. *Cochrane Database of Systematic Reviews* 2012.

Langford R, Bonell CP, Jones HE, Pouliou T, Murphy SM, Waters E, Komro KA, Gibbs LF, Magnus D, Campbell R: **The WHO Health Promoting School framework for improving the health and well-being of students and their academic achievement**. *Cochrane Database of Systematic Reviews* 2014(4).

Lehnert T, Sonntag D, Konnopka A, Riedel-Heller S, Koenig HH: **The long-term cost-effectiveness of obesity prevention interventions: systematic literature review**. *Obesity Reviews* 2012, **13**(6):537-553.

Levy DT, Friend KB: **Strategies for reducing youth access to tobacco: a framework for understanding empirical findings on youth access policies**. *Drugs-Education Prevention and Policy* 2002, **9**(3):285-303.

Macmillan AK, Hosking J, Connor JL, Bullen C, Ameratunga S: **A Cochrane systematic review of the effectiveness of organisational travel plans: improving the evidence base for transport decisions**. *Transport Policy* 2013, **29**:249-256.

Macpherson A, Spinks A: **Bicycle helmet legislation for the uptake of helmet use and prevention of head injuries**. Cochrane Database of Systematic Reviews 2010.

Maniadakis N, Kapaki V, Damianidi L, Kourlaba G: **A systematic review of the effectiveness of taxes on nonalcoholic beverages and high-in-fat foods as a means to prevent obesity trends**. *ClinicoEconomics and Outcomes Research* 2013, **5**(1):519-543.

Manske J: **Efficacy and effectiveness of maternal influenza vaccination during pregnancy: a review of the evidence**. *Maternal & Child Health Journal* 2014, **18**(7):1599-1609.

Matson-Koffman DM, Brownstein JN, Neiner JA, Greaney ML: **A site-specific literature review of policy and environmental interventions that promote physical activity and nutrition for cardiovascular health: what works?** *American Journal of Health Promotion* 2005, **19**(3):167-193.

Mayer K: **Childhood obesity prevention: focusing on the community food environment**. *Family & Community Health* 2009, **32**(3):257-270.

McKinnon RA, Siddiqi SM, Chaloupka FJ, Mancino L, Prasad K: **Obesity-related policy/environmental interventions: a systematic review of economic analyses**. *American Journal of Preventive Medicine* 2016, **50**(4):543-549.

Mema SC, McIntyre L, Musto R: **Childhood vision screening in Canada: public health evidence and practice**. *Canadian Journal of Public Health* 2012, **103**(1):40-45 46p.

Middleton JC, Hahn RA, Kuzara JL, Elder R, Brewer R, Chattopadhyay S, Fielding J, Naimi TS, Toomey T, Lawrence B *et al*: **Effectiveness of Policies Maintaining or Restricting Days of Alcohol Sales on Excessive Alcohol Consumption and Related Harms**. *American Journal of Preventive Medicine* 2010, **39**(6):575-589.

Mischke C, Verbeek J, Job J, Morata T, Alvesalo-Kuusi A, Neuvonen K, Clarke S, Pedlow R: **Occupational safety and health enforcement tools for preventing occupational diseases and injuries**. *Cochrane Database of Systematic Reviews* 2013.

Mozaffarian D, Afshin A, Benowitz NL, Bittner V, Daniels SR, Franch HA, Jacobs DR, Kraus WE, Kris-Etherton PM, Krummel DA *et al*: **Population approaches to improve diet, physical activity, and smoking habits**. *A Scientific Statement From the American Heart Association* 2012, **126**(12):1514-1563.

Munro J, Coleman P, Nicholl J, Harper R, Kent G, Wild D: **Can we prevent accidental injury to adolescents? A systematic review of the evidence**. *Injury Prevention* 1995, **1**(4):249-255.

Murphy-Hoefer R, Griffith R, Pederson LL, Crossett L, Iyer SR, Hiller MD: **A review of interventions to reduce tobacco use in colleges and universities**. *American Journal of Preventive Medicine* 2005, **28**(2):188-200.

Ndiaye S, Hopkins D, Shefer A, Hinman A, Briss P, Rodewald L, Willis B: **Interventions to improve influenza, pneumococcal polysaccharide, and hepatitis B vaccination coverage among high-risk adults: a systematic review**. *American Journal of Preventive Medicine* 2005, **28**(5).

Niebylski ML, Redburn KA, Duhaney T, Campbell NR: **Healthy food subsidies and unhealthy food taxation: a systematic review of the evidence**. *Nutrition* 2015, **31**(6):787-795.

Nuffield Institute for Health (University of Leeds) and NHS Centre for Reviews and Dissemination (University of York): **Preventing unintentional injuries in children and young adolescents**. *Effective Health Care* 1996, **2**(5).

Nuvolone D, Barchielli A, Forastiere F: **Assessing the effectiveness of local transport policies for improvements in urban air quality and public health: a review of scientific literature. [Italian] Valutare l'efficacia degli interventi sulla mobilità urbana ai fini del miglioramento della qualità dell'aria e della salute dei cittadini: una revisione della letteratura scientifica**. *Epidemiologia e prevenzione* 2009, **33**(3):79-87.

Ogilvie D, Egan M, Hamilton V, Petticrew M: **Promoting walking and cycling as an alternative to using cars: systematic review**. *BMJ* 2004, **329**(7469):763-766.

Peek-Asa C: **The effect of random alcohol screening in reducing motor vehicle crash injuries**. *American Journal of Preventive Medicine* 1999, **16**(1, Supplement 1):57-67.

Phillips RO, Ulleberg P, Vaa T: **Meta-analysis of the effect of road safety campaigns on accidents**. *Accident Analysis and Prevention* 2011, **43**(3):1204-1218.

Pilkington P, Kinra S: **Effectiveness of speed cameras in preventing road traffic collisions and related casualties: systematic review**. *BMJ* 2005, **330**:331.

Pucher J, Dill J, Handy S: **Infrastructure, programs, and policies to increase bicycling: an international review**. *Preventive Medicine* 2010, **50**:S106-S125.

Quentin W, Neubauer S, Leidl R, Koenig H-H: **Advertising bans as a means of tobacco control policy: a systematic literature review of time-series analyses**. *International Journal of Public Health* 2007, **52**(5):295-307.

Rammohan V, Hahn RA, Elder R, Brewer R, Fielding J, Naimi TS, Toomey TL, Chattopadhyay SK, Zometa C, Task Force Community Preventive S: **Effects of dram shop liability and enhanced overservice law enforcement initiatives on excessive alcohol consumption and related harms: two community guide systematic reviews**. *American Journal of Preventive Medicine* 2011, **41**(3):334-343.

Rasura M, Baldereschi M, Di C, Di L, Patella R, Piccardi B, Polizzi B, Inzitari D: **Effectiveness of public stroke educational interventions: A review**. *European Journal of Neurology* 2014, **21**(1):11-20.

Reavley N, Jorm AF: **Prevention and early intervention to improve mental health in higher education students: a review** *Early Intervention in Psychiatry* 2010, **4**(2):132-142.

Rivara FP, Thompson DC: **Prevention of falls in the construction industry - evidence for program effectiveness**. *American Journal of Preventive Medicine* 2000, **18**(4):23-26.

Rivara FP, Thompson DC, Cummings P: **Effectiveness of primary and secondary enforced seat belt laws**. *American Journal of Preventive Medicine* 1999, **16**(1):30-39.

Russell Kelly F, Vandermeer B, Hartling L: **Graduated driver licensing for reducing motor vehicle crashes among young drivers**. *Cochrane Database of Systematic Reviews* 2011(10).

Sandhu PK, Elder R, Patel M, Saraiya M, Holman DM, Perna F, Smith RA, Buller D, Sinclair C, Reeder A *et al*: **Community-wide interventions to prevent skin cancer: two community guide systematic reviews**. *American Journal of Preventive Medicine* 2016, **51**(4):531-539.

Scholes-Balog KE, Heerde JA, Hemphill SA: **Alcohol warning labels: unlikely to affect alcohol-related beliefs and behaviours in adolescents**. *Australian and New Zealand Journal of Public Health* 2012, **36**(6):524-529.

Schoueri-Mychasiw N, Campbell S, Mai V: **Increasing screening mammography among immigrant and minority women in Canada: a review of past interventions**. *Journal of Immigrant and Minority Health* 2013, **15**(1):149-158.

Segui-Gomez M: **Evaluating interventions that promote the use of rear seats for children**. *American Journal of Preventive Medicine* 1999, **16**(1):23-29.

Shults RA, Elder RW, Nichols JL, Sleet DA, Compton R, Chattopadhyay SK, Task Force Community P: **Effectiveness of multicomponent programs with community mobilization for reducing alcohol-impaired driving**. *American Journal of Preventive Medicine* 2009, **37**(4):360-371.

Shults RA, Elder RW, Sleet DA, Nichols JL, Alao MO, Carande-Kulis VG, Zaza S, Sosin DM, Thompson RS, Task Force on Community Preventive S: **Reviews of evidence regarding interventions to reduce alcohol-impaired driving**. *American Journal of Preventive Medicine* 2001, **21**(4 Suppl):66-88.

Siegfried N, Pienaar D, Ataguba J, Volmink J, Kredo T, Jere M, Parry C, D H: **Restricting or banning alcohol advertising to reduce alcohol consumption in adults and adolescents**. *Cochrane Database of Systematic Reviews* 2014.

Silver MZ: **Efficacy of anti-tobacco mass media campaigns on adolescent tobacco use**. *Journal of Paediatric Nursing* 2001, **27**(3):293-296.

Singh A, Bassi S, Nazar GP, Saluja K, Park M, Kinra S, Arora M: **Impact of school policies on non-communicable disease risk factors – a systematic review**. *BMC Public Health* 2017, **17**(1):292.

Sipe TA, Finnie RK, Knopf JA, Qu S, Reynolds JA, Thota AB, Hahn RA, Goetzel RZ, Hennessy KD, McKnight-Eily LR *et al*: **Effects of mental health benefits legislation: a community guide systematic review**. *American Journal of Preventive Medicine* 2015, **48**:755-766.

Smulian EA, Mitchell KR, Stokley S: **Interventions to increase HPV vaccination coverage: a systematic review**. *Human Vaccines & Immunotherapeutics* 2016, **12**(6):1566-1588.

Spinks A, Turner C, McClure R, Acton C, Nixon J: **Community-based programmes to promote use of bicycle helmets in children aged 0-14 years: a systematic review**. *International journal of injury control and safety promotion* 2005, **12**(3):131-142.

Spinks A, Turner C, McClure R, Nixon J: **Community based prevention programs targeting all injuries for children**. *Injury Prevention* 2004, **10**(3):180-185.

Stead LF, Lancaster T: **Interventions for preventing tobacco sales to minors**. *Cochrane Database of Systematic Reviews* 2005(1).

Stead M, Gordon R, Angus K, McDermott L: **A systematic review of social marketing effectiveness**. *Health Education* 2007, **107**(2):126-191.

Stead M, Hastings G: **Developing options for a programme on adolescent smoking in Wales**. Health Promotion Wales Technical Report 1995, 1-32.

Stewart G, Anokye NK, Pokhrel S: **What interventions increase commuter cycling? A systematic review**. *BMJ Open* 2015, **5**(8):e007945.

Tan CE, Glantz SA: **Association between smoke-free legislation and hospitalizations for cardiac, cerebrovascular, and respiratory diseases. A meta-analysis**. *Circulation* 2012, **126**(18):2177-2183.

Task Force on Community Preventive Services: **Strategies for reducing exposure to environmental tobacco smoke, increasing tobacco-use cessation, and reducing initiation in communities and health-care systems**. *Morbidity & Mortality Weekly Report* 2000, **49**(44):1-11.

Thorogood M, Simera I, Dowler E, Summerbell C, Brunner E: **A systematic review of population and community dietary interventions to prevent cancer**. *Nutrition Research Reviews* 2007, **20**(1):74-88.

Thow AM, Jan S, Leeder S, Swinburn B: **The effect of fiscal policy on diet, obesity and chronic disease: a systematic review**. *Bulletin of the World Health Organization* 2010, **88**(8):609-614.

Tompa E, Kalcevich C, Foley M, McLeod C, Hogg-Johnson S, Cullen K, MacEachen E, Mahood Q, Irvin E: **A systematic literature review of the effectiveness of occupational health and safety regulatory enforcement**. *American Journal of Industrial Medicine* 2016, **59**(11):919-933.

Towner E, Dowswell T: **Community-based childhood injury prevention interventions: what works?** *Health Promotion International* 2002, **17**(3):273-284.

Trieu K, McMahon E, Santos JA, Bauman A, Jolly KA, Bolam B, Webster J: **Review of behaviour change interventions to reduce population salt intake**. *International Journal of Behavioral Nutrition and Physical Activity* 2017, **14**.

Turner C, McClure R, Nixon J, Spinks A: **Community-based programmes to prevent pedestrian injuries in children 0-14 years: a systematic review**. *Injury Control and Safety Promotion* 2004, **11**(4):231-237.

Turner C, McClure R, Nixon J, Spinks A: **Community-based programs to promote car seat restraints in children 0-16 years: a systematic review**. *Accident Analysis and Prevention* 2005, **37**(1):77-83.

van der Molen HF, Lehtola MM, Lappalainen J, Hoonakker PL, Hsiao H, Haslam R, Hale AR, Frings-Dresen MH, Verbeek JH: **Interventions to prevent injuries in construction workers**. *Cochrane Database of Systematic Reviews* 2012(12).

Vasquez-Morales A, Sanz-Valero J: **Health promotions interventions designed and implemented in aged people over 65 years: a systematic review**. *Revista de Enfermeria* 2011, **34**(11):16-24.

Vernick JS, Li G, Ogaitis S, MacKenzie EJ, Baker SP, Gielen AC: **Effects of high school driver education on motor vehicle crashes, violations, and licensure**. *American Journal of Preventive Medicine* 1999, **16**(1, Supplement 1):40-46.

Vidanapathirana J, Abramson MJ, Forbes A, Fairley C: **Mass media interventions for promoting HIV testing**. *Cochrane Database of Systematic Reviews* 2005.

Wagenaar AC, Toomey TL: **Effects of minimum drinking age laws: review and analyses of the literature from 1960 to 2000**. *Journal of Studies on Alcohol* 2002, **s14**:206-225.

Wang G, Labarthe D: **The cost-effectiveness of interventions designed to reduce sodium intake**. *Journal of Hypertension* 2011, **29**(9):A144.

Warda L, Tenenbein M, Moffatt MEK: **House fire injury prevention update. Part II. A review of the effectiveness of preventive interventions**. *Injury Prevention* 1999, **5**(3):217-225.

Wharton CM, Long M, Schwartz MB: **Changing nutrition standards in schools: the emerging impact on school revenue**. *Journal of School Health* 2008, **78**(5):245-251.

Willemsen MC, De Zwart WM: **The effectiveness of policy and health education strategies for reducing adolescent smoking: a review of the evidence**. *Journal of Adolescence* 1999, **22**(5):587-599.

Williams AJ, Henley WE, Williams CA, Hurst AJ, Logan S, Wyatt KM: **Systematic review and meta-analysis of the association between childhood overweight and obesity and primary school diet and physical activity policies**. *International Journal of Behavioral Nutrition and Physical Activity* 2013, **10**:101.

Wilson C, Willis C, Hendrikz JK, Le Brocque R, Bellamy N: **Speed cameras for the prevention of road traffic injuries and deaths**. *Cochrane Database of Systematic Reviews* 2010(11).

Wilson LM, Avila T, Chander G, Hutton HE, Odelola OA, Elf JL, Heckman-Stoddard BM, Bass EB, Little EA, Haberl EB *et al*: **Impact of tobacco control interventions on smoking initiation, cessation, and prevalence: a systematic review**. *Journal of Environmental Public Health* 2012, **2012**:961724.

Wong VWY, Lok KYW, Tarrant M: **Interventions to increase the uptake of seasonal influenza vaccination among pregnant women: a systematic review**. *Vaccine* 2016, **34**(1):20-32.

Yengopal V, Chikte UM, Mickenautsch S, Oliveira LB, Bhayat A: **Salt fluoridation: a meta-analysis of its efficacy for caries prevention**. *Journal of the South African Dental Association* 2010, **65**(2):60-64, 66-67.

Zaza S, Sleet DA, Thompson RS, Sosin DM, Bolen JC, Task Force Community Preventive S: **Reviews of evidence regarding interventions to increase use of child safety seats**. *American Journal of Preventive Medicine* 2001, **21**(4):31-47.

Zwerling C, Jones MP: **Evaluation of the effectiveness of low blood alcohol concentration laws for younger drivers**. *American Journal of Preventive Medicine* 1999, **16**(1, Supplement 1):76-80.

Insufficient detail is given regarding interventions/outcomes/health inequality data.

Boyce T, Holmes A: **Addressing health inequalities in the delivery of the human papillomavirus vaccination programme: examining the role of the school nurse**. *PLoS ONE* 2012, **7** (9):e43416.

Briss PA, Rodewald LE, Hinman AR, Shefer AM, Strikas RA, Bernier RR, Carande-Kulis VG, Yusuf HR, Ndiaye SM, Williams SM: **Reviews of evidence regarding interventions to improve vaccination coverage in children, adolescents, and adults**. *American Journal of Preventive Medicine* 2000, **18**(1 SUPPL. 1):97-140.

Cawley J, Hull HF, Rousculp MD: **Strategies for implementing school-located influenza vaccination of children: a systematic literature review**. *Journal of School Health* 2010, **80**(4):167-175.

Courtney RJ, Naicker S, Shakeshaft A, Clare P, Martire KA, Mattick RP: **Smoking cessation among low-socioeconomic status and disadvantaged population groups: a systematic review of research output**. *International Journal of Environmental Research and Public Health* 2015, **12**(6):6403-6422.

Delhomme P, Truls V, Thierry M, Harland G, Stina J, Christie N: **Evaluated road safety media campaigns: an overview of 265 evaluated campaigns and some meta-analysis on accidents**. In., vol. Report WP4. Paris: INRETS; 1999.

Derzon JH, Lipsey MW: **A meta-analysis of the effectiveness of mass-communication for changing substance-use knowledge, attitudes, and behavior**. *Mass Media and Drug Prevention: Classic and Contemporary Theories and Research* 2002:231-258.

Elder RW, Lawrence B, Ferguson A, Naimi Timothy S, Brewer Robert D, Chattopadhyay Sajal K, Toomey Traci L, Fielding Jonathan E, Task Force on Community Preventive Services: **Guide to community preventive services: the effectiveness of tax policy interventions for reducing excessive alcohol consumption and related harms**. *American Journal of Preventive Medicine* 2010, **38**(2):217-229.

Faulkner GEJ, Grootendorst P, Van Hai N, Andreyeva T, Arbour-Nicitopoulos K, Auld MC, Cash SB, Cawley J, Donnelly P, Drewnowski A *et al*: **Economic instruments for obesity prevention: results of a scoping review and modified delphi survey**. *International Journal of Behavioral Nutrition and Physical Activity* 2011, **8**:109.

Ganann R, Fitzpatrick-Lewis D, Ciliska D, Peirson L: **Community-based interventions for enhancing access to or consumption of fruit and vegetables among five to 18-year olds: a scoping review**. *BMC Public Health* 2012, **12**:711.

Giskes K, Kunst AE, Ariza C, Benach J, Borrell C, Helmert U, Judge K, Lahelma E, Moussa K, Ostergren PO, Patja K, Platt S, Prattala R, Willemsen MC, Mackenbach JP: **Applying an equity lens to tobacco-control policies and their uptake in six western-European countries**, *Journal of Public Health Policy* 2007, **28**(2): 261-280.

Gouin DM, Gervais C: **Food taxes: too easy a solution**. *Canadian Journal of Diabetes* 2011, **35**(2):216-217.

Gould GS, McEwen A,Watters T, Clough AR, van der Zwan R: **Should anti-tobacco media messages be culturally targeted for Indigenous populations? A systematic review and narrative synthesis**. *Tobacco Control* 2013, **22**(4):1-10.

Hammond D: **Health warning messages on tobacco products: a review**. *Tobacco Control* 2011, **20**(5):327.

Han H-R, Lee J-E, Kim J, Hedlin HK, Song H, Kim MT: **A meta-analysis of interventions to promote mammography among ethnic minority women**. *Nursing Research* 2009, **58**(4):246-254.

Hill S, Amos A, Clifford D, Platt S: **Impact of tobacco control interventions on socioeconomic inequalities in smoking: review of the evidence**. *Tobacco Control* 2014, **23**(S1):e89-97.

Hillier F, Pedley C, Summerbell C: **Evidence base for primary prevention of obesity in children and adolescents**. *Bundesgesundheitsblatt - Gesundheitsforschung - Gesundheitsschutz* 2011, **54**(3):259-264.

Krummel DA, Koffman DM, Bronner Y, Davis J, Greenlund K, Tessaro I, Upson D, Wilbur J: **Cardiovascular health interventions in women: what works?** *Journal of Women's Health & Gender-Based Medicine* 2001, **10**(2):117-136.

Kubacki K, Rundle-Thiele S, Pang B, Buyucek N: **Minimizing alcohol harm: a systematic social marketing review (2000-2014)**. *Journal of Business Research* 2015, **68**(10):2214-2222.

Lin H, Wang H, Wu W, Lang L, Wang Q, Tian L: **The effects of smoke-free legislation on acute myocardial infarction: a systematic review and meta-analysis**. *BMC Public Health* 2013, **13**(1):529.

Mackay DF, Irfan MO, Haw S, Pell JP: **Meta-analysis of the effect of comprehensive smoke-free legislation on acute coronary events**. *Heart* 2010, **96**(19):1525.

Oakley L, Gray R, Kurinczuk JJ, Brocklehurst P, Hollowell J: **Interventions to increase the early initiation of antenatal care in socially disadvantaged and vulnerable women: a systematic review**. *Journal of Epidemiology and Community Health* 2010, **64**:A39-A39.

O'Mara-Eves A, Brunton G, Oliver S, Kavanagh J, Jamal F, Thomas J: **The effectiveness of community engagement in public health interventions for disadvantaged groups: a meta-analysis**. *BMC Public Health* 2015, **15**:129.

Roche A, Kostadinov V, Fischer J, Nicholas R, O'Rourke K, Pidd K, Trifonoff A: **Addressing inequities in alcohol consumption and related harms**. *Health Promotion International* 2015, **30**:20-35.

Salmi L-R, Barsanti S, Bourgueil Y, Daponte A, Piznal E, Ménival S: **Interventions addressing health inequalities in European regions: the AIR project**. *Health Promotion International* 2017, **32**(3):430-441.

Sinha B, Chowdhury R, Sankar MJ, Martines J, Taneja S, Mazumder S, Rollins N, Bahl R, Bhandari N: **Interventions to improve breastfeeding outcomes: a systematic review and meta-analysis**. *Acta Paediatrica, International Journal of Paediatrics* 2015, **104**:114-135.

Task Force on Community Preventive Services: **Vaccine-preventable diseases: improving vaccination coverage in children, adolescents, and adults. A report on recommendations from the Task Force on Community Preventive Services**. *MMRW* 1999, **48**(RR-8):1-15.

Tseng DS, Cox E, Plane MB, Hla KM: **Efficacy of patient letter reminders on cervical cancer screening: a meta-analysis**. *Journal of General Internal Medicine* 2001, **16**(8):563-568.

Verma M, Sarfaty M, Brooks D, Wender RC: **Population-based programs for increasing colorectal cancer screening in the United States**. *CA Cancer* 2015, **65**(6):496-510.

Ward K, Chow MYK, King C, Leask J: **Strategies to improve vaccination uptake in Australia, a systematic review of types and effectiveness**. *Australian and New Zealand Journal of Public Health* 2012, **36**(4):369-377.

<u>**Not a systematic review of primary studies (umbrella review)**</u>

Baird J, Cooper C, Margetts BM, Barker M, Inskip HM: **Changing health behaviour of young women from disadvantaged backgrounds: evidence from systematic reviews**. *Proceedings of the Nutrition Society* 2009, **68**(2):195-204.

Bambra C, Gibson M, Sowden A, Wright K, Whitehead M, Petticrew M: **Tackling the wider social determinants of health and health inequalities: evidence from systematic reviews**. *Journal of Epidemiology and Community Health* 2010, **64**(4):284-291.

Cauchi D, Glonti K, Petticrew M, Knai C: **Environmental components of childhood obesity prevention interventions: an overview of systematic reviews**. *Obesity Reviews* 2016, **17**(11):1116-1130.

Enns J, Holmqvist M, Wener P, Halas G, Rothney J, Schultz A, Goertzen L, Katz A: **Mapping interventions that promote mental health in the general population: a scoping review of reviews**. *Preventive Medicine* 2016, **87**:70-80.

Errington G, Athey K, Towner E, Dickinson H, Brussoni M, Hayes M, Millward L, Taske N: **Interventions to prevent accidental injury to young people aged 15–24: evidence briefing**. In. London: National Institute for Health and Clinical Excellence; 2006: 61.

Fitzgerald N, Angus K, Emslie C, Shipton D, Bauld L: **Gender differences in the impact of population-level alcohol policy interventions: evidence synthesis of systematic reviews**. *Addiction* 2016, **111**(10):1735-1747.

Haby MM, Chapman E, Clark R, Galvao LAC: **Energy interventions that facilitate sustainable development and impact health: an overview of systematic reviews**. *Revista Panamericana De Salud Publica-Pan American Journal of Public Health* 2016, **39**(4):200-207.

Haby MM, Chapman E, Clark R, Galvao LAC: **Interventions that facilitate sustainable jobs and have a positive impact on workers' health: an overview of systematic reviews**. *Revista Panamericana De Salud Publica* 2016, **40**(5):332-340.

Haby MM, Soares A, Chapman E, Clark R, Korc M, Galvao LAC: **Interventions that facilitate sustainable development by preventing toxic exposure to chemicals: an overview of systematic reviews**. *Revista Panamericana De Salud Publica* 2016, **39**(6):378-386.

Hoffman SJ, Tan C: **Overview of systematic reviews on the health-related effects of government tobacco control policies**. *BMC Public Health* 2015, **15**:744.

Hopkins DP, Briss PA, Ricard CJ, Husten CG, Carande-Kulis VG, Fielding JE, Alao MO, McKenna JW, Sharp DJ, Harris JR *et al*: **Reviews of evidence regarding interventions to reduce tobacco use and exposure to environmental tobacco smoke**. *American journal of Preventive Medicine* 2001, **20**(2 Suppl):16-66.

Lorenc T, Petticrew M, Welch V, Tugwell P: **What types of interventions generate inequalities? Evidence from systematic reviews**. *Journal of Epidemiology and Community Health* 2013, **67**(2):190-193.

Main C, Thomas S, Ogilvie D, Stirk L, Petticrew M, Whitehead M, Sowden A: **Population tobacco control interventions and their effects on social inequalities in smoking: placing an equity lens on existing systematic reviews**. *BMC Public Health* 2008, **8:** 178.

Martineau F, Tyner E, Lorenc T, Petticrew M, Lock K: **Population-level interventions to reduce alcohol-related harm: an overview of systematic reviews**. *Preventive Medicine* 2013, **57**(4):278-296.

McCalman J, Bainbridge R, Percival N, Tsey K: **The effectiveness of implementation in Indigenous Australian healthcare: an overview of literature reviews**. *International Journal for Equity in Health* 2016, **15**(1):47.

Morrison DS, Petticrew M, Thomson H: **What are the most effective ways of improving population health through transport interventions? Evidence from systematic reviews**. *Journal of Epidemiology & Community Health* 2003, **57**(5):327-333.

Ogilvie D, Petticrew M: **Reducing social inequalities in smoking: can evidence inform policy? A pilot study**. *Tobacco Control* 2004, **13**(2):129-131.

Health Quality Ontario: **Screening mammography for women aged 40 to 49 years at average risk for breast cancer: an evidence-based analysis**. *Ontario Health Technology Assessment Series* 2007, **7**(1):1-32.

Roche A, Kostadinov V, Fischer J, Nicholas R, O'Rourke K, Pidd K, Trifonoff A: **Addressing inequities in alcohol consumption and related harms**. **Health Promotion International** 2015, **30**, 20-35.

Saraiya M, Glanz K, Briss PA, Nichols P, White C, Das D, Smith SJ, Tannor B, Hutchinson AB, Wilson KM *et al*: **Interventions to prevent skin cancer by reducing exposure to ultraviolet radiation: A systematic review**. *American Journal of Preventive Medicine* 2004, **27**(5):422-466.

Stephens SK, Cobiac LJ, Veerman JL: **Improving diet and physical activity to reduce population prevalence of overweight and obesity: an overview of current evidence**. *Preventive Medicine* 2014, **62**:167-178.

Thomson H, Jepson R, Hurley F, Douglas M: **Assessing the unintended health impacts of road transport policies and interventions: translating research evidence for use in policy and practice**. *BMC Public Health* 2008, **8**(339).

Welch V, Petkovic J, Pardo JP, Rader T, Tugwell P: **Interactive social media interventions to promote health equity: an overview of reviews**. *Health Promotion and Chronic Disease Prevention in Canada-Research Policy and Practice* 2016, **36**(4):63-75.

Wilson MG, Holman PB, Hammock A: **A comprehensive review of the effects of worksite health promotion on health-related outcomes**. *American Journal of Health Promotion* 1996, **10**(6):429-435.

<u>Unable to locate</u>

Baldasseroni A, Olimpi N, Bonaccorsi G: **A systematic review of the effectiveness of workplace safety interventions. [Italian] Revisione sistematica dell'efficacia degli interventi per la prevenzione degli infortuni sul lavoro**. *Medicina del Lavoro* 2009, **100**(4):268-271.

Black M, Yamada J, Bakker R, Brunton G, Cava M, Camiletti Y, Colton P, Harmer M, Mann V, Michel I *et al*: **Community-based strategies to promote cervical cancer screening**. In: *Effective Public Health Practice Project.* City of Hamilton: Social and Public Health Services Division; 2000: 78.

Grilli R, Ramsay C, Minozzi S: **Mass media interventions: effects on health services utilisation**. *Cochrane Database of Systematic Reviews* 2002.

Guerra-Romero L, Noguer I, Bolea A, Suarez M, Parras F: **Preventing HIV infection: an exercise in evaluating scientific evidence in public health. [Spanish] La prevencion de la infeccion por VIH: un ejercicio de evaluacion de las evidencias cientificas en s**. *Enfermedades infecciosas y microbiologia clinica* 1999, **17** (Suppl 2):67-75.

Guy R, Ward JS, Smith KS, Su JY, Huang RL, Tangey A, Skov S, Rumbold A, Silver B, Donovan B *et al*: **The impact of sexually transmissible infection programs in remote Aboriginal communities in Australia: a systematic review**. *Sexual Health* 2012, **9**(3):205-212.

Loveland-Cherry CJ: **Alcohol, children and adolescents**. *Annual Review of Nursing Research* 2005, **23**(1):135-177.

Lund KE, Scheffels J, Sanner T: **How to reduce illegal sales of tobacco to minors?** *Tidsskrift for den Norske lageforening : tidsskrift for praktisk medicin, ny rakke* 1999, **119**(25):3756-3760.

Towner E: **Health promotion in childhood and young adolescence for the prevention of unintentional injuries**. Health Education Authority 1996, London.

Wakefield MA, Wilson D, Owen N, Esterman A, Roberts L: **Workplace smoking restrictions, occupational status, and reduced cigarette consumption**. *Journal of Occupational and Environmental Medicine* 1992, **34**(7):693-697.
